# Supplementary material for: Mega Meta-QTLs: A Strategy for the Production of Golden Barley (Hordeum vulgare L.) Tolerant to Abiotic Stresses
Source: Genes (Basel). 2022 Nov 10;13(11):2087. doi: 10.3390/genes13112087 (PMC9690463; doi:10.3390/genes13112087)
Supplement: Supplementary file 1 [file genes-13-02087-s001.zip › Table S1.pdf]

Table S1. Identifications of QTLs used in Meta QTL analysis

QTL names	Traits	Stress	Chr.	MQTL	LOD score	Phenotypic variance	Peak position	From	To	Reference	Parents
QRsr.S42.1H.a	Root–shoot ratio	Drought	1H	MQTL1.2	3	6.3	39	31.43263197	46.56736803	Arifuzzaman-2014	Scarlett× ISR42-8
QRsr.S42.1H.b	Root–shoot ratio	Drought	1H	MQTL1.2	3	8.3	123.09	117.3460941	128.8339059	Arifuzzaman-2014	Scarlett× ISR42-8
QRdw.S42.1H.a	Root dry weight	Drought	1H	MQTL1.2	3	6.5	39	31.66547406	46.33452594	Arifuzzaman-2014	Scarlett× ISR42-8
QRdw.S42.1H.b	Root dry weight	Drought	1H	MQTL1.2	3	7.9	123.09	117.0552635	129.1247365	Arifuzzaman-2014	Scarlett× ISR42-8
QPh.S42.1H	Plant height	Drought	1H	MQTL1.3	3	0.2	62.23	59.84627907	64.61372093	Arifuzzaman-2014	Scarlett× ISR42-8
QHI.S42.1H	Harvest Index	Drought	1H	MQTL1.3	3	3.11	62.5	1.7875275	123.2124725	Mohammed-Aziz	ISR42-8× Scarlett
QPH.S42.1H	Plant height	Drought	1H	MQTL1.3	3	0.17	62.23	0	173.2981115	Mohammed-Aziz	ISR42-8× Scarlett
qLWD-1	Leaf weight	Drought	1H	MQTL1.3	2.643	11.2	32	25.13510782	38.86489218	Makhtoum-etal-2021	Kavir× Badia
qPm.SEVAD-1H	Severity to powdery Medlow in drought stress	Drought	1H	MQTL1.4	2.603	11	102	95.0102916	108.9897084	Makhtoum-etal-2021	Badia× Kavir
qSND-1	Stomata number	Drought	1H	MQTL1.4	3.874	7.4	58	53.3401944	62.6598056	Makhtoum-etal-2021	Kavir × Badia
QTkw.S42.1H	Thousand kernel weight	Drought	1H	MQTL1.6	3	6.27	115	108.557511	121.442489	Arifuzzaman-2014	Scarlett× ISR42-8
QWS.S42.1H	leaf wilting score	Drought	1H	MQTL1.6	3	7.4	109.4	100.2653509	131.7346491	Mohammed-2012	ISR42-8× Scarlett
QTGW.S42.1H	Thousand grain weight	Drought	1H	MQTL1.6	3	6.27	115	89.48435277	140.5156472	Mohammed-Aziz	ISR42-8× Scarlett
QRSR.S42.1H.a	Root shoot ratio	Drought	1H	MQTL1.6	3	7.4	39	8.885838999	69.114161	Mohammed-Aziz	ISR42-8× Scarlett
QRSR.S42.1H.b	Root shoot ratio	Drought	1H	MQTL1.6	3	8.26	123.09	100.2309456	145.9490544	Mohammed-Aziz	ISR42-8× Scarlett

Continued Table S1.

QTL names	Traits	Stress	Chr.	MQTL	LOD score	Phenotypic variance	Peak position	From	To	Reference	Parents
QRDW.S42.1H.a	Root Dry Weight	Drought	1H	MQTL1.6	3	6.5	39	9.951417004	68.048583	Mohammed-Aziz	ISR42-8× Scarlett
QRDW.S42.1H.b	Root Dry Weight	Drought	1H	MQTL1.6	3	7.85	123.09	99.03703319	147.1429668	Mohammed-Aziz	ISR42-8× Scarlett
QWS.S42.1H	Wilting Score	Drought	1H	MQTL1.6	3	11.96	116	100.2127266	131.7872734	Mohammed-Aziz	ISR42-8× Scarlett
QWS.S42.2H	leaf wilting score	Drought	2H	MQTL2.2	3	5.6	41.8	9.082894737	76.51710526	Mohammed-2012	ISR42-8× Scarlett
qOP$_{i}$-2	leaf osmotic potential	Drought	2H	MQTL2.2	3.2	0.04	9.7	0	49.37065868	Ayman-2004	Tadmor× Er/Apm
qOP$_{i}$-2	leaf osmotic potential	Drought	2H	MQTL2.2	7.9	0.16	31.5	21.58233533	41.41766467	Ayman-2004	Tadmor× Er/Apm
qOP$_{100i}$ -2	leaf osmotic potential	Drought	2H	MQTL2.2	3.6	0.08	37.5	17.66467066	57.33532934	Ayman-2004	Tadmor× Er/Apm
QdPro.2H.1	Free proline content	Drought	2H	MQTL2.2	6.36	12.2	23.805	12.31967213	25.68032787	Kornelia-Gudys-2018	Maresi× Cam/B1/CI08887//CI05761
QdPro.2H.2	Free proline content	Drought	2H	MQTL2.2	10.62	75.2	118.535	117.2162233	119.3837766	Kornelia-Gudys-2018	Maresi× Cam/B1/CI08887//CI05761
QdEth.2H	Ethylene content	Drought	2H	MQTL2.2	3.66	18.5	22.305	13.59359359	23.3053531	Kornelia-Gudys-2018	Maresi× Cam/B1/CI08887//CI05761
QdABS/RC.2H.1	ABS/RC	Drought	2H	MQTL2.2	3.97	17.3	6.865	1.789017331	11.21098266	Kornelia-Gudys-2018	Maresi× Cam/B1/CI08887//CI05761
QdABS/RC.2H.2	ABS/RC	Drought	2H	MQTL2.2	4.09	17.7	120.085	116.3953802	125.6035198	Kornelia-Gudys-2018	Maresi× Cam/B1/CI08887//CI05761
QdWC.2H	Water content	Drought	2H	MQTL2.2	3.82	17.2	14.13	10.36162791	19.83837209	Kornelia-Gudys-2018	Maresi× Cam/B1/CI08887//CI05761
QdWL.2H	Water content	Drought	2H	MQTL2.2	6.84	21.9	14.13	11.37853881	18.82136119	Kornelia-Gudys-2018	Maresi× Cam/B1/CI08887//CI05761
QdTRo/RC.2H.1	Trapped energy flux per PSII reaction center (RC)	Drought	2H	MQTL2.2	3.55	13.4	12.475	8.917910338	21.08208955	Kornelia-Gudys-2018	Maresi× Cam/B1/CI08887//CI05761

Continued Table S1.

QTL names	Traits	Stress	Chr.	MQTL	LOD score	Phenotypic variance	Peak position	From	To	Reference	Parents
QdTRo/RC.2H.2	Trapped energy flux per PSII reaction center (RC)	Drought	2H	MQTL2.2	4.69	16.9	67.245	62.177513379	71.82238521	Kornelia-Gudys-2018	Maresi× Cam/B1/CI08887//CI05761
QRL.S42.2H	Root Length	Drought	2H	MQTL2.2	3	6.13	31.1	10.29807676	71.90192324	Mohammed-Aziz	ISR42-8× Scarlett
QHI.S42.2H	Harvest Index	Drought	2H	MQTL2.2	3	3.81	41.1	0	90.65794999	Mohammed-Aziz	ISR42-8× Scarlett
QWS.S42.2H	Wilting Score	Drought	2H	MQTL2.2	3	5.63	33.79	11.2525596	78.3274404	Mohammed-Aziz	ISR42-8× Scarlett
QDT.TxFr.2H	Drought score	Drought	2H	MQTL2.2	8.56	42.2	24. 2	19.47711954	28.92288046	Yun-Fan-2015	TX9425× Franklin
QRMO.TxFr.2H	relative water content	Drought	2H	MQTL2.2	9.45	45.4	25.2	20.81000979	29.58999021	Yun-Fan-2015	TX9425× Franklin
QRl.S42.2H	root length	Drought	2H	MQTL2.3	3	6.1	41.1	33.28452154	48.91547846	Arifuzzaman-2014	Scarlett× ISR42-8
QSdw.S42.2H.a	Shoot dry weigh	Drought	2H	MQTL2.3	3	14.9	41.1	37.90037459	44.29962541	Arifuzzaman-2014	Scarlett× ISR42-8
QSdw.S42.2H.b	Shoot dry weigh	Drought	2H	MQTL2.3	3	5.8	81	72.78027265	89.21972735	Arifuzzaman-2014	Scarlett× ISR42-8
QSdw.S42.2H.c	Shoot dry weigh	Drought	2H	MQTL2.3	3	4.7	98.21	88.06650668	108.3534933	Arifuzzaman-2014	Scarlett× ISR42-8
QRdw.S42.2H	Root dry weight	Drought	2H	MQTL2.3	3	6.5	44.79	37.45547406	52.12452594	Arifuzzaman-2014	Scarlett× ISR42-8
QRDW.S42.2H	Root Dry Weight	Drought	2H	MQTL2.3	3	6.53	33.79	15.91908418	73.66091582	Mohammed-Aziz	Scarlett× ISR42-8
qPm.AUDPCAD-2H	AUDPC for powdery Medlow in drought stress	Drought	2H	MQTL2.3	3.054	7.58	12.36	2.216623687	22.50337631	Makhtoum-etal-2021	Badia× Kavir
QPH.S42.2H	Plant Height	Drought	2H	MQTL2.3	3	12.96	81	54.59088044	83.72911956	Mohammed-Aziz	Scarlett× ISR42-8
qPLN-2	Plant number	Drought	2H	MQTL2.4	2.511	10.6	24	16.74652901	31.25347099	Makhtoum-etal-2021	Kavir× Badia

Continued Table S1.

QTL names	Traits	Stress	Chr.	MQTL	LOD score	Phenotypic variance	Peak position	From	To	Reference	Parents
QPh.S42.2H	Plant height	Drought	2H	MQTL2.5	3	13	81	77.33273703	84.66726297	Arifuzzaman-2014	Scarlett× ISR42-8
QKER.S42.2H.a	No. of Kernels/spike	Drought	2H	MQTL2.5	3	11.28	41.1	24.36101157	57.83898843	Mohammed-Aziz	Scarlett× ISR42-8
QKER.S42.2H.b	No. of Kernels/spike	Drought	2H	MQTL2.5	3	48.99	77.4	73.54582998	81.25417002	Mohammed-Aziz	Scarlett× ISR42-8
QKER.S42.2H.c	No. of Kernels/spike	Drought	2H	MQTL2.5	3	37.96	90	85.02592757	94.97407243	Mohammed-Aziz	Scarlett× ISR42-8
QSPS.S42.2H.a	Number of spikes/plant	Drought	2H	MQTL2.5	3	40.95	81	76.38911381	85.61088619	Mohammed-Aziz	Scarlett× ISR42-8
QSPS.S42.2H.b	Number of spikes/plant	Drought	2H	MQTL2.5	3	34.8	90	84.57425892	95.42574108	Mohammed-Aziz	Scarlett× ISR42-8
QTILS.S42.2H.a	Number of tillers/plant	Drought	2H	MQTL2.5	3	39.86	81	76.26302585	85.73697415	Mohammed-Aziz	ISR42-8× Scarlett
QTILS.S42.2H.b	Number of tillers/plant	Drought	2H	MQTL2.5	3	35.99	90	84.75365964	95.24634036	Mohammed-Aziz	ISR42-8× Scarlett
QRWC.S42.2H.a	Root dry weight	Drought	2H	MQTL2.5	3	11.5	42	25.5812357	58.4187643	Mohammed-Aziz	ISR42-8× Scarlett
QRWC.S42.2H.b	Root dry weight	Drought	2H	MQTL2.5	3	5.7	80	46.8744229	113.1255771	Mohammed-Aziz	ISR42-8× Scarlett
QRWC.S42.2H.c	Root dry weight	Drought	2H	MQTL2.5	3	14.96	90	77.3786237	102.6213763	Mohammed-Aziz	ISR42-8× Scarlett
QSDW.S42.2H.a	Shoot dry weight/plant	Drought	2H	MQTL2.5	3	14.88	41.1	28.41076684	53.78923316	Mohammed-Aziz	ISR42-8× Scarlett
QSDW.S42.2H.b	Shoot dry weight/plant	Drought	2H	MQTL2.5	3	5.82	81	48.55742449	113.4425755	Mohammed-Aziz	ISR42-8× Scarlett
QSDW.S42.2H.c	Shoot dry weight/plant	Drought	2H	MQTL2.5	3	4.73	98.21	58.29122844	138.1287716	Mohammed-Aziz	ISR42-8× Scarlett
qLWD-2	Leaf weight	Drought	2H	MQTL2.7	3.653	15.2	108	102.9416584	113.0583416	Makhtoum-etal-2021	Kavir× Badia

Continued Table S1.

QTL names	Traits	Stress	Chr.	MQTL	LOD score	Phenotypic variance	Peak position	From	To	Reference	Parents
qRWD-2	Root weight	Drought	2H	MQTL2.7	3.482	14.5	90	84.69746259	95.30253741	Makhtoum-etal-2021	Kavir× Badia
qWSC$_{s4}$-2	water-soluble carbohydrate concentration	Drought	2H	MQTL2.7	4.8	0.15	175.2	164.6211577	185.7788423	Ayman-2004	Tadmor× Er/Apm
qWSC$_{i4}$-2	water-soluble carbohydrate concentration	Drought	2H	MQTL2.7	3.7	0.07	20.9	0	43.56894782	Ayman-2004	Tadmor× Er/Apm
qWSC$_{i4}$-2	water-soluble carbohydrate concentration	Drought	2H	MQTL2.7	4.2	0.08	46.5	26.66467066	66.33532934	Ayman-2004	Tadmor× Er/Apm
qWSC$_{100s4}$-2	water-soluble carbohydrate concentration	Drought	2H	MQTL2.7	2.9	0.05	24.9	0	56.63652695	Ayman-2004	Tadmor× Er/Apm
qWSC$_{100s4}$-2	water-soluble carbohydrate concentration	Drought	2H	MQTL2.7	3.5	0.05	102.7	70.96347305	134.4365269	Ayman-2004	Tadmor× Er/Apm
qWSC$_{100s4}$-2	water-soluble carbohydrate concentration	Drought	2H	MQTL2.7	4.8	0.11	175.2	160.7743059	189.6256941	Ayman-2004	Tadmor× Er/Apm
qDWSC$_{100.4}$-2	water-soluble carbohydrate concentration	Drought	2H	MQTL2.7	2.8	0.05	27.5	0	59.23652695	Ayman-2004	Tadmor× Er/Apm
qDWSC$_{100.4}$-2	water-soluble carbohydrate concentration	Drought	2H	MQTL2.7	2.6	0.08	181.2	161.3646707	201.0353293	Ayman-2004	Tadmor× Er/Apm
QGY.S42.2H.a	Grain Yield/plant	Drought	2H	MQTL2.7	3	5.87	44.79	12.6237667	76.9562333	Mohammed-Aziz	ISR42-8× Scarlett
QGY.S42.2H.a	Grain Yield/plant	Drought	2H	MQTL2.7	3	7.6	98.21	73.36581717	123.0541828	Mohammed-Aziz	ISR42-8× Scarlett
QdETo/RC.2H	Electron transport flux per PSII reaction center (RC)	Drought	2H	MQTL2.8	3.85	20.5	143.9	139.9243902	147.8756098	Kornelia-Gudys-2018	Maresi× Cam/B1/CI08887//CI05761
QPC-D.TxFr.3H	Proline content	Drought	3H	MQTL3.1	6.65	34.7	70	64.25632405	75.74367595	Yun-Fan-2015	TX9425× Franklin
qSCD-3	Drought score	Drought	3H	MQTL3.1	2.594	11.1	16	9.073261941	22.92673806	Makhtoum-etal-2021	Kavir× Badia
qOA-3	Osmotic adjustment	Drought	3H	MQTL3.1	3.7	0.07	0	0	22.66894782	Ayman-2004	Tadmor× Er/Apm

Continued Table S1.

QTL names	Traits	Stress	Chr.	MQTL	LOD score	Phenotypic variance	Peak position	From	To	Reference	Parents
qRWC$_{s}$-3	Relative water content	Drought	3H	MQTL3.1	3	0.05	257.7	225.9634731	289.4365269	Ayman-2004	Tadmor× Er/Apm
qRWC$_{i}$-3	Relative water content	Drought	3H	MQTL3.1	2.6	0.09	80.8	63.16859614	98.43140386	Ayman-2004	Tadmor× Er/Apm
QPH.S42.3H.a	Plant height	Drought	3H	MQTL3.2	3	7.85	65.86	41.80703319	89.91296681	Mohammed-Aziz	ISR42-8× Scarlett
QPH.S42.3H.b	Plant height	Drought	3H	MQTL3.2	3	59.16	63.65	60.4583876	66.8416124	Mohammed-Aziz	ISR42-8× Scarlett
QSPS.S42.3H	No. of spikes/plant	Drought	3H	MQTL3.2	3	9.93	66	47.00444774	84.99555226	Mohammed-Aziz	ISR42-8× Scarlett
QKER.S42.3H	No. of Kernels/spike	Drought	3H	MQTL3.2	3	0.6	51.4	0	366.0929825	Mohammed-Aziz	ISR42-8× Scarlett
qPm.AUDPCAD-3H	AUDPC for powdery Medlow in drought stress	Drought	3H	MQTL3.2	85.817	41.04	84.55	82.67654014	86.42345986	Makhtoum-etal-2021	Badia× Kavir
qDWSC$_{100.4}$-3	water-soluble carbohydrate concentration	Drought	3H	MQTL3.3	4.5	0.10	4.0	0	19.86826347	Ayman-2004	Tadmor× Er/Apm
qDWSC$_{100.4}$-3	water-soluble carbohydrate concentration	Drought	3H	MQTL3.3	3.6	0.08	38.7	18.86467066	58.53532934	Ayman-2004	Tadmor× Er/Apm
qDWSC$_{100.4}$-3	water-soluble carbohydrate concentration	Drought	3H	MQTL3.3	3.2	0.11	76.8	62.37430593	91.22569407	Ayman-2004	Tadmor× Er/Apm
qDWSC$_{100.4}$-3	water-soluble carbohydrate concentration	Drought	3H	MQTL3.3	3.7	0.08	191.2	171.3646707	211.0353293	Ayman-2004	Tadmor× Er/Apm
qWSC$_{100s4}$-3	water-soluble carbohydrate concentration	Drought	3H	MQTL3.3	3.9	0.07	43.6	20.93105218	66.26894782	Ayman-2004	Tadmor× Er/Apm
qWSC$_{100s4}$-3	water-soluble carbohydrate concentration	Drought	3H	MQTL3.3	5.9	0.26	84.8	78.69682174	90.90317826	Ayman-2004	Tadmor× Er/Apm
qWSC$_{100s4}$-3	water-soluble carbohydrate concentration	Drought	3H	MQTL3.3	5.7	0.09	191.2	173.5685961	208.8314039	Ayman-2004	Tadmor× Er/Apm
qWSC$_{100i4}$-3	water-soluble carbohydrate concentration	Drought	3H	MQTL3.3	3.5	0.11	203.2	188.7743059	217.6256941	Ayman-2004	Tadmor× Er/Apm

Continued Table S1.

QTL names	Traits	Stress	Chr.	MQTL	LOD score	Phenotypic variance	Peak position	From	To	Reference	Parents
QWS.S42.3H	Wilting score	Drought	3H	MQTL3.4	3	33.92	118.72	113.1534968	124.2865032	Mohammed-Aziz	ISR42-8× Scarlett
QHI.S42.3H.a	Harvest index	Drought	3H	MQTL3.4	3	14.71	50.43	37.59412036	63.26587964	Mohammed-Aziz	ISR42-8× Scarlett
QHI.S42.3H.b	Harvest index	Drought	3H	MQTL3.4	3	22.98	63	54.78347304	71.21652696	Mohammed-Aziz	ISR42-8× Scarlett
QHI.S42.3H.c	Harvest index	Drought	3H	MQTL3.4	3	23.15	118.72	110.5638104	126.8761896	Mohammed-Aziz	ISR42-8× Scarlett
QGY.S42.3H.a	Grain yield/plant	Drought	3H	MQTL3.4	3	7.22	50.43	24.2782286	76.5817714	Mohammed-Aziz	ISR42-8× Scarlett
QGY.S42.3H.b	Grain yield/plant	Drought	3H	MQTL3.4	3	12.57	66	50.97885525	81.02114475	Mohammed-Aziz	ISR42-8× Scarlett
QGY.S42.3H.c	Grain yield/plant	Drought	3H	MQTL3.4	3	14.34	118.72	105.5529296	131.8870704	Mohammed-Aziz	ISR42-8× Scarlett
QWS.S42.3H	leaf wilting score	Drought	3H	MQTL3.4	3	34	118.7	113.1465944	124.2534056	Mohammed-2012	ISR42-8× Scarlett
QTkw.S42.3H	Thousand kernel weight	Drought	3H	MQTL3.4	3	5.9	111.7	103.6195901	119.7804099	Arifuzzaman-2014	Scarlett× ISR42-8
QPh.S42.3H.a	Plant height	Drought	3H	MQTL3.4	3	7.9	100.7	94.66526347	106.7347365	Arifuzzaman-2014	Scarlett× ISR42-8
QPh.S42.3H.b	Plant height	Drought	3H	MQTL3.4	3	59.2	118.72	117.9146889	119.5253111	Arifuzzaman-2014	Scarlett× ISR42-8
QRdw.S42.3H	Root dry weight	Drought	3H	MQTL3.4	3	7.9	118.72	112.6852635	124.7547365	Arifuzzaman-2014	Scarlett× ISR42-8
QRsr.S42.3H	Root–shoot ratio	Drought	3H	MQTL3.4	3	7.4	118.72	112.277511	125.162489	Arifuzzaman-2014	Scarlett× ISR42-8
QRl.S42.3H	Root length	Drought	3H	MQTL3.4	3	5.5	118.72	110.0519239	127.3880761	Arifuzzaman-2014	Scarlett× ISR42-8
QRL.S42.3H	Root length	Drought	3H	MQTL3.5	3	5.52	118.72	84.51424104	152.925759	Mohammed-Aziz	Scarlett× ISR42-8

Continued Table S1.

QTL names	Traits	Stress	Chr.	MQTL	LOD score	Phenotypic variance	Peak position	From	To	Reference	Parents
QTGW.S42.3H	Thousand grain weight	Drought	3H	MQTL3.5	3	5.85	111.7	79.42379667	143.9762033	Mohammed-Aziz	ISR42-8× Scarlett
QRWC.S42.3H	Relative water content	Drought	3H	MQTL3.5	3	5.6	118.72	85.00289474	152.4371053	Mohammed-Aziz	ISR42-8× Scarlett
QRSR.S42.3H	Root–shoot ratio	Drought	3H	MQTL3.5	3	7.35	118.72	93.03077694	144.4092231	Mohammed-Aziz	ISR42-8× Scarlett
QRDW.S42.3H	Root dry weight	Drought	3H	MQTL3.5	3	7.88	118.72	94.7586054	142.6813946	Mohammed-Aziz	ISR42-8× Scarlett
QPC.S42.3H	Proline content	Drought	3H	MQTL3.6	3	6.1	175.2	144.2465919	206.1534081	Mohammed-2012	ISR42-8× Scarlett
qOP$_{i}$-3	Osmotic potential	Drought	3H	MQTL3.6	2.8	0.04	0	0	39.67065868	Ayman-2004	Tadmor× Er/Apm
qOP$_{i}$-3	Osmotic potential	Drought	3H	MQTL3.6	3.8	0.08	74.8	54.96467066	94.63532934	Ayman-2004	Tadmor× Er/Apm
qOP$_{1000i}$-3	Osmotic potential	Drought	3H	MQTL3.6	6.8	0.15	70.5	59.92115768	81.07884232	Ayman-2004	Tadmor× Er/Apm
qOP$_{1000i}$-3	Osmotic potential	Drought	3H	MQTL3.6	4.3	0.07	302.9	280.2310522	325.5689478	Ayman-2004	Tadmor× Er/Apm
QPC.S42.3H	Proline content	Drought	3H	MQTL3.6	3	6.13	175.24	144.4380768	206.0419232	Mohammed-Aziz	ISR42-8× Scarlett
qFLW-4a	Flag leaf weight	Drought	4H	MQTL4.2	3.652	15.1	68	62.90815944	73.09184056	Makhtoum-etal-2021	Kavir× Badia
qFLW-4b	Flag leaf weight	Drought	4H	MQTL4.2	3.581	14.8	150	144.8049465	155.1950535	Makhtoum-etal-2021	Kavir× Badia
qLND-4	Leaf number	Drought	4H	MQTL4.2	2.949	12.5	136	129.8490566	142.1509434	Makhtoum-etal-2021	Kavir× Badia
qRLD-4a	Root length	Drought	4H	MQTL4.2	2.701	11.4	2	0	8.744455478	Makhtoum-etal-2021	Badia× Kavir
qRLD-4b	Root length	Drought	4H	MQTL4.2	5.51	21.8	70	66.47308292	73.52691708	Makhtoum-etal-2021	Badia× Kavir

Continued Table S1.

QTL names	Traits	Stress	Chr.	MQTL	LOD score	Phenotypic variance	Peak position	From	To	Reference	Parents
qRLD-4c	Root length	Drought	4H	MQTL4.2	2.941	12.3	136	129.7490413	142.2509587	Makhtoum-etal-2021	Badia× Kavir
qRWD-4a	Root length	Drought	4H	MQTL4.5	2.955	12.4	12	5.799452222	18.20054778	Makhtoum-etal-2021	Badia× Kavir
qRWD-4b	Root length	Drought	4H	MQTL4.5	3.539	14.6	70	64.73378134	75.26621866	Makhtoum-etal-2021	Badia× Kavir
qPWD-4	Plumule weight	Drought	4H	MQTL4.5	3.849	15.8	68	63.13374731	72.86625269	Makhtoum-etal-2021	Badia× Kavir
qPm.SEVAD-4Ha	Severity for powdery Medlow in drought stress	Drought	4H	MQTL4.5	4.094	16.7	70	65.39600045	74.60399955	Makhtoum-etal-2021	Badia× Kavir
qPm.SEVAD-4Hb	Severity for powdery Medlow in drought stress	Drought	4H	MQTL4.5	3.111	13	136	130.0856313	141.9143687	Makhtoum-etal-2021	Badia× Kavir
qPm.AUDPCAD-4	AUDPC for powdery Medlow in drought stress	Drought	4H	MQTL4.6	2.598	11	68	61.0102916	74.9897084	Makhtoum-etal-2021	Badia× Kavir
QPH.S42.4H.a	Plant height	Drought	4H	MQTL4.6	3	5.93	68.1	36.25922606	99.94077394	Makhtoum-etal-2021	ISR42-8× Scarlett
QPH.S42.4H.b	Plant height	Drought	4H	MQTL4.6	3	7.03	68.44	41.58142397	95.29857603	Mohammed-Aziz	ISR42-8× Scarlett
QPC.S42.4H	Proline content	Drought	4H	MQTL4.8	3	4.2	134	86.04385965	175.9561404	Mohammed-Aziz	ISR42-8× Scarlett
QTkw.S42.4H.a	Thousand kernel weight	Drought	4H	MQTL4.8	3	1.4	131	96.94684385	165.0531561	Mohammed-2012	Scarlett× ISR42-8
QTkw.S42.4H.b	Thousand kernel weight	Drought	4H	MQTL4.8	3	9.1	141.1	135.8610529	146.3389471	Arifuzzaman-2014	Scarlett× ISR42-8
QRdw.S42.4H	Root dry weight	Drought	4H	MQTL4.8	3	3.4	131	116.9781122	145.0218878	Arifuzzaman-2014	Scarlett× ISR42-8
QPh.S42.4H.a	Plant height	Drought	4H	MQTL4.8	3	5.9	131	122.9195901	139.0804099	Arifuzzaman-2014	Scarlett× ISR42-8
QPh.S42.4H.b	Plant height	Drought	4H	MQTL4.8	3	7	146	139.1893688	152.8106312	Arifuzzaman-2014	Scarlett× ISR42-8

Continued Table S1.

Original QTL	Original QTL name	Trait	location	MQTL	LOD score	Phenotypic variance	position	from	to	reference	parents
QWS.S42.4H	Leaf wilting score	Drought	4H	MQTL4.8	3	9.4	143.1	120.1132139	160.2867861	Mohammed-2012	Scarlett× ISR42-8
QPC.S42.4H	Proline Content	Drought	4H	MQTL4.8	3	4.19	131.5	85.93656576	176.0634342	Mohammed-Aziz	Scarlett× ISR42-8
QKER.S42.4H	No. of Kernels/spike	Drought	4H	MQTL4.8	3	6.96	127.5	100.3712946	154.6287054	Mohammed-Aziz	Scarlett× ISR42-8
QHI.S42.4H	Harvest index	Drought	4H	MQTL4.8	3	7.78	130	105.7306183	154.2693817	Mohammed-Aziz	Scarlett× ISR42-8
QOP.S42.4H	Osmotic Potential	Drought	4H	MQTL4.8	3	6.75	141.1	113.1272904	169.0727096	Mohammed-Aziz	Scarlett× ISR42-8
QWS.S42.4H	Wilting Score	Drought	4H	MQTL4.8	3	9.41	140.2	120.1345601	160.2654399	Mohammed-Aziz	Scarlett× ISR42-8
QTGW.S42.4H.a	Thousand grain weight	Drought	4H	MQTL4.8	3	1.35	131	0	270.8635478	Mohammed-Aziz	Scarlett× ISR42-8
QTGW.S42.4H.b	Thousand grain weight	Drought	4H	MQTL4.8	3	9.13	141.1	120.4191906	161.7808094	Mohammed-Aziz	Scarlett× ISR42-8
QSPS.S42.4H.a	No. of Spikes/plant	Drought	4H	MQTL4.8	3	17.53	127.5	116.7289909	138.2710091	Mohammed-Aziz	Scarlett× ISR42-8
QSPS.S42.4H.b	No. of Spikes/plant	Drought	4H	MQTL4.8	3	18.75	140	129.9298246	150.0701754	Mohammed-Aziz	Scarlett× ISR42-8
QTILS.S42.4H.a	No. of Tillers/plant	Drought	4H	MQTL4.8	3	15.18	127.5	115.0615422	139.9384578	Mohammed-Aziz	Scarlett× ISR42-8
QTILS.S42.4H.b	No. of Tillers/plant	Drought	4H	MQTL4.8	3	14.27	140.2	126.9683399	153.4316601	Mohammed-Aziz	Scarlett× ISR42-8
qPDL-5	Peduncle length	Drought	5H	MQTL5.4	2.593	10.9	42	34.94616583	49.05383417	Makhtoum-etal-2021	Kavir× Badia
qRWD-5	Root weight	Drought	5H	MQTL5.4	3.636	15	112	106.8742138	117.1257862	Makhtoum-etal-2021	Badia× Kavir
qRLD-5	Root length	Drought	5H	MQTL5.5	4.595	18.6	108	103.8663015	112.1336985	Makhtoum-etal-2021	Badia× Kavir

Continued Table S1.

QTL names	Traits	Stress	Chr.	MQTL	LOD score	Phenotypic variance	Peak position	From	To	Reference	Parents
QSdw.S42.5H	Shoot dry weight	Drought	5H	MQTL5.8	3	3.6	126.77	113.5271059	140.0128941	Arifuzzaman-2014	Scarlett× ISR42-8
QRsr.S42.5H	Root–shoot ratio	Drought	5H	MQTL5.8	3	3.2	126.77	111.8717442	141.6682558	Arifuzzaman-2014	Scarlett× ISR42-8
QPC.S42.5H	Proline content	Drought	5H	MQTL5.8	3	4.1	95	48.94736842	141.0526316	Mohammed-2012	Scarlett× ISR42-8
QPC.S42.5H	Proline content	Drought	5H	MQTL5.8	3	74	95	48.60791413	141.3920859	Mohammed-Aziz	Scarlett× ISR42-8
QRL.S42.5H	Root length	Drought	5H	MQTL5.8	3	1.73	125.1	15.95792516	234.2420748	Mohammed-Aziz	Scarlett× ISR42-8
QRSR.S42.5H	Root shoot ratio	Drought	5H	MQTL5.8	3	3.16	126.77	67.01816789	186.5218321	Mohammed-Aziz	Scarlett× ISR42-8
QSDW.S42.5H	Soot dry weight/plant	Drought	5H	MQTL5.8	3	3.64	126.77	74.89753036	178.6424696	Mohammed-Aziz	Scarlett× ISR42-8
QRl.S42.5H	Root length	Drought	5H	MQTL5.8	3	1.7	125.1	97.05622	153.1437756	Mohammed-Aziz	Scarlett× ISR42-8
QHI.S42.5H.a	Harvest index	Drought	5H	MQTL5.8	3	6.97	33	5.91021671	60.08978328	Mohammed-Aziz	Scarlett× ISR42-8
QHI.S42.5H.b	Harvest index	Drought	5H	MQTL5.8	3	5.82	126	91.79424104	160.205759	Mohammed-Aziz	Scarlett× ISR42-8
QRDW.S42.5H	Root dry weight	Drought	5H	MQTL5.8	3	4.21	126.77	81.92064383	171.6193562	Mohammed-Aziz	Scarlett× ISR42-8
qPm.AUDPCAD-5	AUDPC for powdery Medlow in drought stress	Drought	5H	MQTL5.9	3.689	15.2	138	132.9416584	143.0583416	Makhtoum-etal-2021	Badia× Kavir
qPm.SEVAD-5H	Severity for powdery Medlow in drought stress	Drought	5H	MQTL5.9	6.626	25.6	138	134.9966097	141.0033903	Makhtoum-etal-2021	Badia× Kavir
QPC.S42.6H	Proline content	Drought	6H	MQTL6.3	3	4	76.3	27.79605263	122.2039474	Mohammed-2012	Scarlett× ISR42-8
QTkw.S42.6H.a	Thousand kernel weight	Drought	6H	MQTL6.3	3	1.6	63	33.20348837	92.79651163	Arifuzzaman-2014	Scarlett× ISR42-8

Continued Table S1.

QTL names	Traits	Stress	Chr.	MQTL	LOD score	Phenotypic variance	Peak position	From	To	Reference	Parents
QTkw.S42.6H.b	Thousand kernel weight	Drought	6H	MQTL6.3	3	1.9	72.7	47.60820073	97.79179927	Arifuzzaman-2014	Scarlett× ISR42-8
QSdw.S42.6H	Shoot dry weight	Drought	6H	MQTL6.3	3	6.5	68.1	60.76547406	75.43452594	Arifuzzaman-2014	Scarlett× ISR42-8
QGY.S42.6H	Grain Yield/plant	Drought	6H	MQTL6.3	3	13.17	68	53.66318987	82.33681013	Mohammed-Aziz	Scarlett× ISR42-8
QTGW.S42.6H.a	Thousand grain weight	Drought	6H	MQTL6.3	3	1.64	63	0	178.1315789	Mohammed-Aziz	Scarlett× ISR42-8
QTGW.S42.6H.b	Thousand grain weight	Drought	6H	MQTL6.3	3	1.89	72.7	0	172.6025341	Mohammed-Aziz	Scarlett× ISR42-8
QPC.S42.6H	Proline content	Drought	6H	MQTL6.3	3	3.94	71.5	27.07721079	122.9227892	Mohammed-Aziz	Scarlett× ISR42-8
QSDW.S42.6H	Shoot dry Weight/plant	Drought	6H	MQTL6.3	3	6.5	68.1	39.051417	97.148583	Mohammed-Aziz	Scarlett× ISR42-8
QTILS.S42.6H	No. of tillers/plant	Drought	6H	MQTL6.3	3	8.83	84.64	63.25655838	106.0234416	Mohammed-Aziz	Scarlett× ISR42-8
QKER.S42.6H	No. of kernels/spike	Drought	6H	MQTL6.3	3	3.5	120	66.05263158	173.9473684	Mohammed-Aziz	Scarlett× ISR42-8
QHI.S42.6H	Harvest index	Drought	6H	MQTL6.3	3	8.36	90	67.41437925	112.5856208	Mohammed-Aziz	Scarlett× ISR42-8
QSPS.S42.6H.a	No. of spikes/plant	Drought	6H	MQTL6.3	3	11.71	75	58.87567981	91.12432019	Mohammed-Aziz	Scarlett× ISR42-8
QSPS.S42.6H.b	No. of spikes/plant	Drought	6H	MQTL6.3	3	7.26	91.99	65.9823155	117.9976845	Mohammed-Aziz	Scarlett× ISR42-8
qFLW-6	Flag leaf width	Drought	6H	MQTL6.3	2.558	10.8	96	88.88085255	103.1191474	Makhtoum-etal-2021	Badia× Kavir
qFLW-6a	Flag leaf width	Drought	6H	MQTL6.3	2.949	12.4	56	49.79945222	62.20054778	Makhtoum-etal-2021	Badia× Kavir
qFLW-6b	Flag leaf width	Drought	6H	MQTL6.3	3.238	13.5	98	92.30468204	103.695318	Makhtoum-etal-2021	Badia× Kavir

Continued Table S1.

QTL names	Traits	Stress	Chr.	MQTL	LOD score	Phenotypic variance	Peak position	From	To	Reference	Parents
qPm.SEVAD-6Hb	Severity for powdery Medlow in drought stress	Drought	6H	MQTL6.3	2.563	10.8	56	48.88085255	63.11914745	Makhtoum-etal-2021	Badia× Kavir
qPm.SEVAD-6Hc	Severity for powdery Medlow in drought stress	Drought	6H	MQTL6.3	8.29	3.1	98	73.19780889	122.8021911	Makhtoum-etal-2021	Badia× Kavir
qPm.AUDPCAD-6	AUDPC for powdery Medlow in drought stress	Drought	6H	MQTL6.4	4.271	17.4	98	93.58121882	102.4187812	Makhtoum-etal-2021	Badia× Kavir
qSPW-6	Spike weight	Drought	6H	MQTL6.4	2.583	10.9	50	42.94616583	57.05383417	Makhtoum-etal-2021	Kavir× Badia
QTkw.S42.7H	Thousand kernel weight	Drought	7H	MQTL7.1	3	9.6	100.3	95.33391473	105.2660853	Arifuzzaman-2014	Scarlett× ISR42-8
QTGW.S42.7H	Thousand grain weight	Drought	7H	MQTL7.1	3	9.61	100.3	80.6521551	119.9478449	Mohammed-Aziz	Scarlett× ISR42-8
QRDW.S42.7H	Root Dry Weight	Drought	7H	MQTL7.2	3	6.91	42.5	15.17499429	69.82500571	Mohammed-Aziz	Scarlett× ISR42-8
QRSR.S42.7H	Root Shoot Ratio	Drought	7H	MQTL7.2	3	6.6	42.5	13.89154705	71.10845295	Mohammed-Aziz	Scarlett× ISR42-8
QRdw.S42.7H	Root dry weight	Drought	7H	MQTL7.2	3	6.9	42.5	35.59066397	49.40933603	Arifuzzaman-2014	Scarlett× ISR42-8
QRsr.S42.7H	Root–shoot ratio	Drought	7H	MQTL7.2	3	6.6	42.5	35.27660324	49.72339676	Arifuzzaman-2014	Scarlett× ISR42-8
QdWL.7H_2	Water loss rate	Drought	7H	MQTL7.2	3.15	10.6	105	97.31132075	112.6886792	Kornelia-Gudys-2018	Maresi× Cam/B1/CI08887//CI05761
Qd(1-B)av.7H_2	The average fraction of open RC during the time needed to complete the closure of all RCs	Drought	7H	MQTL7.2	3.33	18.7	117.8	113.4417112	122.1582888	Kornelia-Gudys-2018	Maresi× Cam/B1/CI08887//CI05761
qPm.AUDPCAD-7	AUDPC for powdery Medlow in drought stress	Drought	7H	MQTL7.6	9.916	12.2	154	147.6978039	160.3021961	Makhtoum-etal-2021	Badia× Kavir
qFLL-7	Flag leaf length	Drought	7H	MQTL7.6	3.299	13.7	122	116.3878254	127.6121746	Makhtoum-etal-2021	Kavir× Badia
qPT-7	Peduncle diameter	Drought	7H	MQTL7.6	3.213	13.4	122	116.2621797	127.7378203	Makhtoum-etal-2021	Kavir× Badia

Continued Table S1.

QTL names	Traits	Stress	Chr.	MQTL	LOD score	Phenotypic variance	Peak position	From	To	Reference	Parents
qINL-7	Internode length	Drought	7H	MQTL7.6	3.167	13.2	124	118.175243	129.824757	Makhtoum-etal-2021	Kavir× Badia
qSCD-7	Drought score	Drought	7H	MQTL7.6	2.544	10.9	38	30.94616583	45.05383417	Makhtoum-etal-2021	Kavir× Badia
qLWD-7	Leaf weight	Drought	7H	MQTL7.6	2.865	12.1	40	33.64571963	46.35428037	Makhtoum-etal-2021	Kavir× Badia
qPm.SEVAD-7Ha	Severity for powdery Medlow in drought stress	Drought	7H	MQTL7.8	5.208	20.8	92	88.30351959	95.69648041	Makhtoum-etal-2021	Badia× Kavir
qPm.SEVAD-7Hb	Severity for powdery Medlow in drought stress	Drought	7H	MQTL7.8	3.498	14.5	154	148.6974626	159.3025374	Makhtoum-etal-2021	Badia× Kavir
qDWSC$_{100.4}$-7	Drought accumulation of water-soluble carbohydrate concentration	Drought	7H	MQTL7.9	3.2	0.08	206.3	186.4646707	226.1353293	Ayman-2004	Tadmor× Er/Apm
qWSC$_{i4}$-7	water-soluble carbohydrate concentration	Drought	7H	MQTL7.10	2.8	0.06	36.7	10.25289421	63.14710573	Ayman-2004	Tadmor× Er/Apm
qWSC$_{i4}$-7	water-soluble carbohydrate concentration	Drought	7H	MQTL7.10	2.7	0.05	97.2	65.46347305	128.9365269	Ayman-2004	Tadmor× Er/Apm
qWSC$_{100s4}$-7	water-soluble carbohydrate concentration	Drought	7H	MQTL7.10	3.6	0.05	90.8	59.06347305	122.5365269	Ayman-2004	Tadmor× Er/Apm
qWSC$_{100s4}$-7	water-soluble carbohydrate concentration	Drought	7H	MQTL7.10	2.7	0.06	114.8	88.35289421	141.2471058	Ayman-2004	Tadmor× Er/Apm
qWSC$_{100s4}$-7	water-soluble carbohydrate concentration	Drought	7H	MQTL7.10	3.8	0.06	129.7	103.2528942	156.1471058	Ayman-2004	Tadmor× Er/Apm
qWSC$_{100s4}$-7	water-soluble carbohydrate concentration	Drought	7H	MQTL7.10	2.9	0.04	227.3	195.5634731	253.0365269	Ayman-2004	Tadmor× Er/Apm
qRWC$_{s}$ -7	Relative water content	Drought	7H	MQTL7.10	3.1	0.05	129.7	97.96347305	161.4365269	Ayman-2004	Tadmor× Er/Apm
qRWC$_{i}$ -7	Relative water content	Drought	7H	MQTL7.10	2.9	0.07	68	45.33105218	90.66894782	Ayman-2004	Tadmor× Er/Apm
qRWC$_{i}$ -7	Relative water content	Drought	7H	MQTL7.10	3.5	0.07	246.6	223.9310522	269.2689478	Ayman-2004	Tadmor× Er/Apm

Continued Table S1.

QTL names	Traits	Stress	Chr.	MQTL	LOD score	Phenotypic variance	Peak position	From	To	Reference	Parents
qTMC-Ap3-1H	TMC-Ap3 accumulation	low-temperature	1H-L	MQTL1.1	5.2	17.4	4.7	0	10.76406356	Francia-2004	Nure× Tremois
qCOLD-1H-L	Cold score	low temperature	1H	MQTL1.5	5.7	0.08	82	62.28846154	101.7115385	Jeffrey-2005	Dicktoo× Morex
qTMC-Ap3-2H	TMC-Ap3 accumulation	low-temperature	2H	MQTL2.1	21.6	55.2	0.3	0	2.211498295	Francia-2004	Nure× Tremois
qCOLD-4S	Cold score	low temperature	4H	MQTL4.8	3.7	0.04	8	0	47.42307692	Jeffrey-2005	Dicktoo× Morex
qCOLD-4L	Cold score	low temperature	4H	MQTL4.8	3	0.04	68	28.57692308	107.4230769	Jeffrey-2005	Dicktoo× Morex
qFTS-5H	Frost tolerance	low-temperature	5H	MQTL5.2	9.2	30.7	1.3	0	4.736961104	Francia-2004	Nure× Tremois
qFTS-5H	Frost tolerance	low-temperature	5H	MQTL5.2	9.1	30.6	5	1.551806997	8.448193003	Francia-2004	Nure× Tremois
qFT-5H	Fv/Fm value	low-temperature	5H	MQTL5.2	13.1	45.3	5.7	3.370757045	8.029242955	Francia-2004	Nure× Tremois
qFT-5H	Fv/Fm value	low-temperature	5H	MQTL5.2	13.7	46.8	1.3	0	3.554587733	Francia-2004	Nure× Tremois
qWS-5H	Winter survival	low-temperature	5H	MQTL5.2	6.7	21.5	3.1	0	8.007660739	Francia-2004	Nure× Tremois
qWS-5H	Winter survival	low-temperature	5H	MQTL5.2	12.6	36.6	6.4	3.517084539	9.282915461	Francia-2004	Nure× Tremois
qCOR14ba -5H	COR14b accumulation	low-temperature	5H	MQTL5.2	24.3	63.2	1.3	0	2.969536485	Francia-2004	Nure× Tremois
qTMC-Ap3-5H	TMC-Ap3 accumulation	low-temperature	5H	MQTL5.2	14.2	45.3	1.3	0	3.629242955	Francia-2004	Nure× Tremois
qVrnH1-5H	Vernalization requirement (VrnH1)	low-temperature	5H	MQTL5.2	10.5	35	6.4	3.385294118	9.414705882	Francia-2004	Nure× Tremois
qCOR14ba -6H	COR14b accumulation	low-temperature	6H	MQTL6.1	3.7	14.1	0.6	0	8.083312474	Francia-2004	Nure× Tremois

Continued Table S1.

QTL names	Traits	Stress	Chr.	MQTL	LOD score	Phenotypic variance	Peak position	From	To	Reference	Parents
qTMC-Ap3-6H	TMC-Ap3 accumulation	low-temperature	6H	MQTL6.1	4.4	15.2	9.6	2.658243034	16.54175697	Francia-2004	Nure× Tremois
QTgw.S42.1H.a	Thousand grain weight	nitrogen stress	1H	MQTL1.1	3	0.4	14	2.372093023	25.62790698	Bernhard-Saal-etal-2011	ISR42-8× Scarlett
QTgw.S42.1H.b	Thousand grain weight	nitrogen stress	1H	MQTL1.1	3	0.7	85	77.05426357	92.94573643	Bernhard-Saal-etal-2011	ISR42-8× Scarlett
QTgw.S42.1H.c	Thousand grain weight	nitrogen stress	1H	MQTL1.1	3	0.2	144	139.2797605	148.7202395	Bernhard-Saal-etal-2011	ISR42-8× Scarlett
Qsur.yf.1H	Plant survival	manganese toxicity	1H	MQTL1.3	3.73	6.1	101.5	88.98692013	113.0130799	X.Huang-etal-2018	Yerong× Franklin
Q-PhN1-2005-MQM	Plant height	Nitrogen stress	1H	MQTL1.5	4.76	8.6	104.5	94.0859954	114.9140046	Gashu-Aynalem-Kindu-etal-2014	Prisma× Apex R
QHei.S42.1H.a	Plant height	Nitrogen stress	1H	MQTL1.6	3	4.4	144	133.1649049	154.8350951	Bernhard-Saal-etal-2011	ISR42-8× Scarlett
QHei.S42.1H.b	Plant height	Nitrogen stress	1H	MQTL1.6	3	2.8	162	133.9733219	179.0265781	Bernhard-Saal-etal-2011	ISR42-8× Scarlett
QEar.S42.1H.a	Number of ears	Nitrogen stress	1H	MQTL1.6	3	5.6	162	153.486711	170.513289	Bernhard-Saal-etal-2011	ISR42-8× Scarlett
QEar.S42.1H.b	Number of ears	Nitrogen stress	1H	MQTL1.6	3	4.8	144	134.0678295	153.9321705	Bernhard-Saal-etal-2011	ISR42-8× Scarlett
QHea.S42.1H.a	Days until heading	Nitrogen stress	1H	MQTL1.6	3	4	130	118.0813953	141.9186047	Bernhard-Saal-etal-2011	ISR42-8× Scarlett
QHea.S42.1H.b	Days until heading	Nitrogen stress	1H	MQTL1.6	3	3.4	39	24.97811218	53.02188782	Bernhard-Saal-etal-2011	ISR42-8× Scarlett
QHea.S42.1H.c	Days until heading	Nitrogen stress	1H	MQTL1.6	3	0.2	105	0	343.372093	Bernhard-Saal-etal-2011	ISR42-8× Scarlett
QHei.S42.2H.a	Plant height	Nitrogen stress	2H	MQTL2.2	3	6.6	17	9.776603242	24.22339676	Bernhard-Saal-etal-2011	ISR42-8× Scarlett
QHei.S42.2H.b	Plant height	Nitrogen stress	2H	MQTL2.2	3	19.4	86	83.54255574	88.45744426	Bernhard-Saal-etal-2011	ISR42-8× Scarlett

Continued Table S1.

QTL names	Traits	Stress	Chr.	MQTL	LOD score	Phenotypic variance	Peak position	From	To	Reference	Parents
QHei.S42.2H.c	Plant height	Nitrogen stress	2H	MQTL2.2	3	0.1	146	0	622.744186	Bernhard-Saal-etal-2011	ISR42-8× Scarlett
QYld.S42.2H.a	Grain yield	Nitrogen stress	2H	MQTL2.2	3	1.4	27	0	61.05315615	Bernhard-Saal-etal-2011	ISR42-8× Scarlett
QYld.S42.2H.b	Grain yield	Nitrogen stress	2H	MQTL2.2	3	1.3	92	55.3273703	128.6726297	Bernhard-Saal-etal-2011	ISR42-8× Scarlett
QYld.S42.2H.c	Grain yield	Nitrogen stress	2H	MQTL2.2	3	4.9	122	112.2705268	131.7294732	Bernhard-Saal-etal-2011	ISR42-8× Scarlett
QYld.S42.2H.d	Grain yield	Nitrogen stress	2H	MQTL2.2	3	0.4	42	0	161.1860465	Bernhard-Saal-etal-2011	ISR42-8× Scarlett
QTgw.S42.2H.a	Thousand grain weight	Nitrogen stress	2H	MQTL2.3	3	3.7	27	21.11426931	32.88573069	Bernhard-Saal-etal-2011	ISR42-8× Scarlett
QHea.S42.2H.a	Days until heading	Nitrogen stress	2H	MQTL2.4	3	29.9	42	40.40553784	43.59446216	Bernhard-Saal-etal-2011	ISR42-8× Scarlett
QHea.S42.2H.b	Days until heading	Nitrogen stress	2H	MQTL2.4	3	4.2	146	134.648948	157.351052	Bernhard-Saal-etal-2011	ISR42-8× Scarlett
QEar.S42.2H.a	number of ears	Nitrogen stress	2H	MQTL2.6	3	6.5	17	9.665474061	24.33452594	Bernhard-Saal-etal-2011	ISR42-8× Scarlett
QEar.S42.2H.b	number of ears	Nitrogen stress	2H	MQTL2.6	3	56.1	86	85.15018862	86.84981138	Bernhard-Saal-etal-2011	ISR42-8× Scarlett
Q-TkwN0-2008-MQM	Thousand kernel weight	Nitrogen stress	3H	MQTL3.2	3.34	13.3	74.2	67.46613236	80.93386764	Gashu-Aynalem-Kindu-etal-2014	Prisma× Apex R
QYld.S42.3H.a	Grain yield	Nitrogen stress	3H	MQTL3.2	3	16.4	25	22.09302326	27.90697674	Bernhard-Saal-etal-2011	ISR42-8× Scarlett
QYld.S42.3H.b	Grain yield	Nitrogen stress	3H	MQTL3.2	3	9.1	155	149.7610529	160.2389471	Bernhard-Saal-etal-2011	ISR42-8× Scarlett
QHea.S42.3H.a	Days until heading	Nitrogen stress	3H	MQTL3.2	3	5	25	15.46511628	34.53488372	Bernhard-Saal-etal-2011	ISR42-8× Scarlett
QHea.S42.3H.b	Days until heading	Nitrogen stress	3H	MQTL3.2	3	7.9	155	148.9652635	161.0347365	Bernhard-Saal-etal-2011	ISR42-8× Scarlett

Continued Table S1.

QTL names	Traits	Stress	Chr.	MQTL	LOD score	Phenotypic variance	Peak position	From	To	Reference	Parents
QHei.S42.3H.a	Plant height	Nitrogen stress	3H	MQTL3.2	3	10.8	49	44.58570198	53.41429802	Bernhard-Saal-etal-2011	ISR42-8× Scarlett
QHei.S42.3H.b	Plant height	Nitrogen stress	3H	MQTL3.2	3	30.4	155	153.4317625	156.5682375	Bernhard-Saal-etal-2011	ISR42-8× Scarlett
qALU-3	Aluminum content	Aluminum Tolerance	3H	MQTL3.5	2.15	0.11	115	101.1218569	128.8781431	S.NAVAKODE-etal-2009	OWBDOM× OWBREC
qALU-3	Aluminum content	Aluminum Tolerance	3H	MQTL3.5	1.69	0.09	121	104.0378251	137.9621749	S.NAVAKODE-etal-2009	OWBDOM× OWBREC
QTgw.S42.3H.a	Thousand grain weight	Nitrogen stress	3H	MQTL3.5	3	4.8	110	105.9937463	114.0062537	Bernhard-Saal-etal-2011	ISR42-8× Scarlett
QTgw.S42.3H.b	Thousand grain weight	Nitrogen stress	3H	MQTL3.5	3	7.8	175	168.8878951	181.1121049	Bernhard-Saal-etal-2011	ISR42-8× Scarlett
Q-ShwN2-2005-MQM	Sheath weight	Nitrogen stress	3H	MQTL3.5	4.34	15.5	125.2	119.4219071	130.9780929	Gashu-Aynalem-Kindu-etal-2014	Prisma× Apex R
Q-PhN2-2005-MQM	Plant height	Nitrogen stress	3H	MQTL3.5	17.94	54.4	125.2	123.5536684	126.8463316	Gashu-Aynalem-Kindu-etal-2014	Prisma× Apex R
Q-DsN2-2005-MQM	Stem weight	Nitrogen stress	3H	MQTL3.5	11.03	41.8	126.2	124.0574058	128.3425942	Gashu-Aynalem-Kindu-etal-2014	Prisma× Apex R
Q-PhN1-2005-MQM	Plant height	Nitrogen stress	3H	MQTL3.5	19.61	51.0	125.2	123.4439129	126.9560871	Gashu-Aynalem-Kindu-etal-2014	Prisma× Apex R
Q-SwN2-2005-MQM	Straw weight	Nitrogen stress	3H	MQTL3.5	7.87	25.9	128.4	124.942068	131.857932	Gashu-Aynalem-Kindu-etal-2014	Prisma× Apex R
Q-AgNupN2-2005-MQM	Nitrogen uptake	Nitrogen stress	3H	MQTL3.5	7.11	25.2	125.2	121.6460143	128.7539857	Gashu-Aynalem-Kindu-etal-2014	Prisma× Apex R
Q-PhN3-2005-MQM	Plant height	Nitrogen stress	3H	MQTL3.5	8.35	33.9	126.2	123.5580991	128.8419009	Gashu-Aynalem-Kindu-etal-2014	Prisma× Apex R
Q-PhN0-2008-MQM	Plant height	Nitrogen stress	3H	MQTL3.5	11.34	42.6	126.2	124.0976423	128.3023577	Gashu-Aynalem-Kindu-etal-2014	Prisma× Apex R
Q-DsN1-2005-MQM	Stem weight	Nitrogen stress	3H	MQTL3.5	7.86	24.9	125.2	121.6031952	128.7968048	Gashu-Aynalem-Kindu-etal-2014	Prisma× Apex R

Continued Table S1.

QTL names	Traits	Stress	Chr.	MQTL	LOD score	Phenotypic variance	Peak position	From	To	Reference	Parents
Q-NUEbN2-2005-MQM	Nitrogen use efficiency	Nitrogen stress	3H	MQTL3.5	6.84	20.8	125.2	120.8942096	129.5057904	Gashu-Aynalem-Kindu-etal-2014	Prisma× Apex R
Q-NupEbN2-2005-MQM	Nitrogen uptake efficiency	Nitrogen stress	3H	MQTL3.5	7.98	27.8	133.4	130.1784015	136.6215985	Gashu-Aynalem-Kindu-etal-2014	Prisma× Apex R
Q-AgbN2-2005-MQM	Above-ground biomass	Nitrogen stress	3H	MQTL3.5	7.12	25.4	125.2	121.6739984	128.7260016	Gashu-Aynalem-Kindu-etal-2014	Prisma× Apex R
Q-PhN0-2005-MQM	Plant height	Nitrogen stress	3H	MQTL3.5	13.89	38.5	126.2	123.8737548	128.5262452	Gashu-Aynalem-Kindu-etal-2014	Prisma× Apex R
Q-DsN0-2005-MQM	Stem weight	Nitrogen stress	3H	MQTL3.5	5.91	25.2	126.2	122.6460143	129.7539857	Gashu-Aynalem-Kindu-etal-2014	Prisma× Apex R
QHea.S42.4H.a	Plant height	Nitrogen stress	4H	MQTL4.3	3	5.2	19	9.831842576	28.16815742	Bernhard-Saal-etal-2011	ISR42-8× Scarlett
QHea.S42.4H.b	Plant height	Nitrogen stress	4H	MQTL4.3	3	1.6	150	120.2034884	179.7965116	Bernhard-Saal-etal-2011	ISR42-8× Scarlett
qALU-4	Aluminum content	Aluminum Tolerance	4H	MQTL4.5	1.87	0.11	53	39.12185687	66.87814313	S.NAVAKODE-etal-2009	OWBDOM× OWBREC
QTgw.S42.4H.a	Thousand grain weight	Nitrogen stress	4H	MQTL4.7	3	4.3	95	83.91292591	106.0870741	Bernhard-Saal-etal-2011	ISR42-8× Scarlett
QTgw.S42.4H.b	Thousand grain weight	Nitrogen stress	4H	MQTL4.7	3	18.4	131	128.408999	133.591001	Bernhard-Saal-etal-2011	ISR42-8× Scarlett
QTgw.S42.4H.c	Thousand grain weight	Nitrogen stress	4H	MQTL4.7	3	0.7	31	0	99.10631229	Bernhard-Saal-etal-2011	ISR42-8× Scarlett
QHei.S42.4H.a	Plant height	Nitrogen stress	4H	MQTL4.7	3	20	180	177.6162791	182.3837209	Bernhard-Saal-etal-2011	ISR42-8× Scarlett
QHei.S42.4H.b	Plant height	Nitrogen stress	4H	MQTL4.7	3	0.2	21	-217.372093	259.372093	Bernhard-Saal-etal-2011	ISR42-8× Scarlett
QHei.S42.4H.c	Plant height	Nitrogen stress	4H	MQTL4.7	3	6.9	130	123.090664	136.909336	Bernhard-Saal-etal-2011	ISR42-8× Scarlett
QEar.S42.4H.a	Ears per m^2	Nitrogen stress	4H	MQTL4.7	3	5.5	21	12.33192389	29.66807611	Bernhard-Saal-etal-2011	ISR42-8× Scarlett

Continued Table S1.

QTL names	Traits	Stress	Chr.	MQTL	LOD score	Phenotypic variance	Peak position	From	To	Reference	Parents
QEar.S42.4H.b	Number of ears	Nitrogen stress	4H	MQTL4.7	3	16.9	130	127.1790285	132.8209715	Bernhard-Saal-etal-2011	ISR42-8× Scarlett
QEar.S42.4H.c	Number of ears	Nitrogen stress	4H	MQTL4.7	3	34.2	180	178.6060112	181.3939888	Bernhard-Saal-etal-2011	ISR42-8× Scarlett
QYld.S42.4H.a	Grain yield	Nitrogen stress	4H	MQTL4.8	3	3.7	170	157.115022	182.884978	Bernhard-Saal-etal-2011	ISR42-8× Scarlett
QYld.S42.4H.b	Grain yield	Nitrogen stress	4H	MQTL4.8	3	0.4	31	0	150.1860465	Bernhard-Saal-etal-2011	ISR42-8× Scarlett
QYld.S42.4H.c	Grain yield	Nitrogen stress	4H	MQTL4.8	3	0.5	95	0	190.3488372	Bernhard-Saal-etal-2011	ISR42-8× Scarlett
QYld.S42.5H.a	Grain yield	Nitrogen stress	5H	MQTL5.4	3	5.1	43	33.65207478	52.34792522	Bernhard-Saal-etal-2011	ISR42-8× Scarlett
QYld.S42.5H.b	Grain yield	Nitrogen stress	5H	MQTL5.4	3	0.7	69	0.893687708	137.1063123	Bernhard-Saal-etal-2011	ISR42-8× Scarlett
QYld.S42.5H.c	Grain yield	Nitrogen stress	5H	MQTL5.4	3	3.6	137	123.7571059	150.2428941	Bernhard-Saal-etal-2011	ISR42-8× Scarlett
QHei.S42.5H.a	Plant height	Nitrogen stress	5H	MQTL5.4	3	8	43	37.04069767	48.95930233	Bernhard-Saal-etal-2011	ISR42-8× Scarlett
QEar.S42.5H.b	Number of ears	Nitrogen stress	5H	MQTL5.4	3	7.8	43	36.88789505	49.11210495	Bernhard-Saal-etal-2011	ISR42-8× Scarlett
Q-LwN2-2005-MQM	Leaf weight	Nitrogen stress	5H	MQTL5.9	2.94	13.4	95.7	89.01638511	102.3836149	Gashu-Aynalem-Kindu-etal-2014	Prisma× Apex R
Q-LwN0-2005-MQM	Leaf weight	Nitrogen stress	5H	MQTL5.9	3.08	14.0	94.7	88.30282575	101.0971743	Gashu-Aynalem-Kindu-etal-2014	Prisma× Apex R
qCP-6	CP at maturity carboxypeptidase	Nitrogen stress	6H	MQTL6.2	2.75	8.4	286	279.3545336	292.6454664	Litao-Yang-etal-2004	'Lewis' (CI15856)× 'Karl' (CI15487)
QTgw.S42.6H.a	Thousand grain weight	Nitrogen stress	6H	MQTL6.3	3	0.7	40	0	108.1063123	Bernhard-Saal-etal-2011	ISR42-8× Scarlett
QTgw.S42.6H.b	Thousand grain weight	Nitrogen stress	6H	MQTL6.3	3	1.5	103	71.21705426	134.7829457	Bernhard-Saal-etal-2011	ISR42-8× Scarlett

Continued Table S1.

QTL names	Traits	Stress	Chr.	MQTL	LOD score	Phenotypic variance	Peak position	From	To	Reference	Parents
QTgw.S42.6H.c	Thousand grain weight	Nitrogen stress	6H	MQTL6.3	3	0.3	6	0	164.9147287	Bernhard-Saal-etal-2011	ISR42-8× Scarlett
QTgw.S42.6H.d	Thousand grain weight	Nitrogen stress	6H	MQTL6.3	3	1.1	112	68.65961945	155.3403805	Bernhard-Saal-etal-2011	ISR42-8× Scarlett
QYld.S42.6H.a	Grain yield	Nitrogen stress	6H	MQTL6.3	3	5.9	112	103.9195901	120.0804099	Bernhard-Saal-etal-2011	ISR42-8× Scarlett
QYld.S42.6H.b	Grain yield	Nitrogen stress	6H	MQTL6.3	3	0.2	6	0	244.372093	Bernhard-Saal-etal-2011	ISR42-8× Scarlett
QEar.S42.6H.a	Number of ears	Nitrogen stress	6H	MQTL6.3	3	7.8	135	128.8878951	141.1121049	Bernhard-Saal-etal-2011	ISR42-8× Scarlett
QEar.S42.6H.b	Number of ears	Nitrogen stress	6H	MQTL6.3	3	6.1	96	88.18452154	103.8154785	Bernhard-Saal-etal-2011	ISR42-8× Scarlett
QHei.S42.6H.a	Plant height	Nitrogen stress	6H	MQTL6.3	3	3.3	103	88.55320648	117.4467935	Bernhard-Saal-etal-2011	ISR42-8× Scarlett
QLC.yf.6H	Leaf chlorosis	manganese toxicity	6H	MQTL6.4	3.97	7.6	74.5	63.35660693	85.53339306	X.Huang-etal-2018	Yerong× Franklin
QSur.yf.6H	Plant survival	manganese toxicity	6H	MQTL6.4	3.44	6.8	78	66.77503129	89.22396871	X.Huang-etal-2018	Yerong× Franklin
QHea.S42.7H.a	Days until heading	Nitrogen stress	7H	MQTL7.1	3	3.7	19	6.115021999	31.884978	Bernhard-Saal-etal-2011	ISR42-8× Scarlett
QHea.S42.7H.b	Days until heading	Nitrogen stress	7H	MQTL7.1	3	4.2	146	134.648948	157.351052	Bernhard-Saal-etal-2011	ISR42-8× Scarlett
QHei.S42.7H.a	Plant height	Nitrogen stress	7H	MQTL7.1	3	5.4	27	18.17140396	35.82859604	Bernhard-Saal-etal-2011	ISR42-8× Scarlett
QHei.S42.7H.b	Plant height	Nitrogen stress	7H	MQTL7.1	3	3.1	62	46.62115529	77.37884471	Bernhard-Saal-etal-2011	ISR42-8× Scarlett
QHei.S42.7H.c	Plant height	Nitrogen stress	7H	MQTL7.1	3	0.1	146	0	622.744186	Bernhard-Saal-etal-2011	ISR42-8× Scarlett
QYld.S42.7H.a	Grain yield	Nitrogen stress	7H	MQTL7.1	3	0.2	27	0	265.372093	Bernhard-Saal-etal-2011	ISR42-8× Scarlett

Continued Table S1.

QTL names	Traits	Stress	Chr.	MQTL	LOD score	Phenotypic variance	Peak position	From	To	Reference	Parents
QYld.S42.7H.b	Grain yield	Nitrogen stress	7H	MQTL7.1	3	3.9	146	133.7757901	158.2242099	Bernhard-Saal-etal-2011	ISR42-8× Scarlett
QYld.S42.7H.c	Grain yield	Nitrogen stress	7H	MQTL7.1	3	0.2	75	47.1627907	94.8372093	Bernhard-Saal-etal-2011	ISR42-8× Scarlett
QYld.S42.7H.d	Grain yield	Nitrogen stress	7H	MQTL7.1	3	0.2	181	157.1627907	204.8372093	Bernhard-Saal-etal-2011	ISR42-8× Scarlett
QEar.S42.7H.a	Number of ears	Nitrogen stress	7H	MQTL7.1	3	1.7	27	0	55.04377565	Bernhard-Saal-etal-2011	ISR42-8× Scarlett
QEar.S42.7H.b	Number of ears	Nitrogen stress	7H	MQTL7.1	3	0.5	62	0	157.3488372	Bernhard-Saal-etal-2011	ISR42-8× Scarlett
QΔN-5H	N remobilization efficiency	Nitrogen stress	7H	MQTL7.4	4.26	12.70	39	31.07737922	46.92262078	Mei-Han-etal-2016	Morex× Barke
QΔN-7H.52-64	N remobilization efficiency	Nitrogen stress	7H	MQTL7.4	3.59	10.8	58	48.68358482	67.31641518	Mei-Han-etal-2016	Morex× Barke
QYld-7H	Biomass	Nitrogen stress	7H	MQTL7.4	4.40	13.2	241	233.3774785	248.6225215	Mei-Han-etal-2016	Morex× Barke
QYld-1H	Biomass	Nitrogen stress	7H	MQTL7.4	3.59	10.8	235	225.6835848	244.3164152	Mei-Han-etal-2016	Morex× Barke
QYld-7H.70	Biomass	Nitrogen stress	7H	MQTL7.4	3	10.99	70.4	61.24465114	79.55534886	Mei-Han-etal-2016	Morex× Barke
QYld-7H.84	Biomass	Nitrogen stress	7H	MQTL7.4	3	10.46	84.92	75.30075679	94.53924321	Mei-Han-etal-2016	Morex× Barke
QYld-7H.54-58	Biomass	Nitrogen stress	7H	MQTL7.4	5.4	6.02	56.285	39.57116546	72.99883454	Mei-Han-etal-2016	Morex× Barke
QGpc-2H.42	Grain protein content	Nitrogen stress	7H	MQTL7.7	3	1.57	121.09	57.00255799	185.177442	Mei-Han-etal-2016	Morex× Barke
QGpc-7H.86	Grain protein content	Nitrogen stress	7H	MQTL7.7	3	1.43	88.06	16.03826297	156.761737	Mei-Han-etal-2016	Morex× Barke
QGpc-7H.130	Grain protein content	Nitrogen stress	7H	MQTL7.7	3	1.14	130.64	41.6492246	218.1707754	Mei-Han-etal-2016	Morex× Barke

Continued Table S1.

QTL names	Traits	Stress	Chr.	MQTL	LOD score	Phenotypic variance	Peak position	From	To	Reference	Parents
QTgw.S42.7H.a	Thousand grain weight	Nitrogen stress	7H	MQTL7.10	3	4	152	140.0813953	163.9186047	Bernhard-Saal-etal-2011	ISR42-8× Scarlett
QTgw.S42.7H.b	Thousand grain weight	Nitrogen stress	7H	MQTL7.10	3	0.5	62	-33.34883721	157.3488372	Bernhard-Saal-etal-2011	ISR42-8× Scarlett
Aerated LRL	Longest root length	Waterlogging	1H	MQTL1.1	2.97	20	17	11.3055556	22.69444444	Sue-Broughton-etal-2015	Franklin× YYXT
Stagnant LRL	Longest root length	Waterlogging	1H	MQTL1.1	3.69	20	35	29.30555556	40.69444444	Sue-Broughton-etal-2015	Franklin× YYXT
Stagnant SDW	Shoot dry weight	Waterlogging	1H	MQTL1.2	3.35	20	40.5	34.80555556	46.19444444	Sue-Broughton-etal-2015	Franklin× YYXT
Aerated SDW	Shoot dry weight	Waterlogging	1H	MQTL1.2	3.65	20	3.3	0	8.994444444	Sue-Broughton-etal-2015	Franklin× YYXT
Aerated SFW	Shoot fresh weight	Waterlogging	1H	MQTL1.2	3.1	20	40.5	34.80555556	46.19444444	Sue-Broughton-etal-2015	Franklin× YYXT
Stagnant SFW	Shoot fresh weight	Waterlogging	1H	MQTL1.2	3.68	20	1.47	0	7.164444444	Sue-Broughton-etal-2015	Franklin× YYXT
Aerated SFW	Shoot fresh weight	Waterlogging	1H	MQTL1.2	3.1	20	1.5	0	10.19444444	Sue-Broughton-etal-2015	Franklin× YYXT
Stagnant RFW	Root fresh weight	Waterlogging	1H	MQTL1.3	3.37	20	58	52.30555556	63.69444444	Sue-Broughton-etal-2015	Franklin× YYXT
Aerated RDW	Root dry weight	Waterlogging	1H	MQTL1.3	3.17	20	58	52.30555556	63.69444444	Sue-Broughton-etal-2015	Franklin× YYXT
Stagnant RDW	Root dry weight	Waterlogging	1H	MQTL1.3	3.17	20	58	52.30555556	63.69444444	Sue-Broughton-etal-2015	Franklin× YYXT
tfy1.1-3	Leaf chlorosis	Waterlogging	1H	MQTL1.6	2.75	7.1	64	42.03123086	85.96876914	Haobing-Li-etal-2008	Franklin× TX9425
QWl.YyFr.2H	Waterlogging score	Waterlogging	2H	MQTL2.4	15.28	22.8	76.1	72.44077927	79.75922073	Meixue-Zhou-2012	Erleng× Franklin
GYw1.1	Grain yield	Waterlogging	2H	MQTL2.4	2.85	4.74	83.85	29.78881857	137.9111874	Da-wei-XUE-et_al-_2010	Yerong× Franklin

Continued Table S1.

QTL names	Traits	Stress	Chr.	MQTL	LOD score	Phenotypic variance	Peak position	From	To	Reference	Parents
GSw1.1	Grain yield	Waterlogging	2H	MQTL2.5	11.53	35.35	77.95	70.70106082	85.19893918	Da-wei-XUE-et_al-_2010	Yerong× Franklin
GSw1.2	Grain yield	Waterlogging	2H	MQTL2.5	6.29	12.18	105.6	84.56141215	126.6385878	Da-wei-XUE-et_al-_2010	Yerong× Franklin
SLw2.1	Spike length	Waterlogging	2H	MQTL2.5	11.16	17.44	83.85	69.15676606	98.54323394	Da-wei-XUE-et_al-_2010	Yerong× Franklin
SLw2.2	Spike length	Waterlogging	2H	MQTL2.5	8.74	13.05	112.1	92.46398467	131.7360153	Da-wei-XUE-et_al-_2010	Yerong× Franklin
tfy1.1-1	Leaf chlorosis	Waterlogging	2H	MQTL2.5	9.21	23.3	33	26.30565404	39.69434596	Haobing-Li-etal-2008	Franklin× TX9425
tfsur-1	Plant survival	Waterlogging	2H	MQTL2.7	3.29	19	57	48.7906178	65.20938215	Haobing-Li-etal-2008	Franklin× TX9425
tfsur-2	Plant survival	Waterlogging	2H	MQTL2.7	2.75	13.2	16.5	4.683465086	28.31653491	Haobing-Li-etal-2008	Franklin× TX9425
Stagnant LRL	Longest root length	Waterlogging	2H	MQTL2.8	3.53	20	156	150.3055556	161.6944444	Sue-Broughton-etal-2015	Franklin× YYXT
QWl.YyFr.3H	Waterlogging score	Waterlogging	3H	MQTL3.1	9.83	13.6	5.2	0	11.33457592	Meixue-Zhou-2012	Erleng× Franklin
Aerated Tiller	Tiller number	Waterlogging	3H	MQTL3.2	4.87	20	24	18.3055556	29.69444444	Sue-Broughton-etal-2015	Franklin× YYXT
Stagnant Tiller	Tiller number	Waterlogging	3H	MQTL3.2	5.63	20	35.9	30.2055556	41.59444444	Sue-Broughton-etal-2015	Franklin× YYXT
yfy2.1-2	Leaf yellowing	Waterlogging	3H	MQTL3.2	6.41	11.9	47	40.18710535	53.81289465	Haobing-Li-etal-2008	Franklin× Yerong
yfy2.2-1	Leaf yellowing	Waterlogging	3H	MQTL3.2	4.5	9.5	47.5	38.96595302	56.03404698	Haobing-Li-etal-2008	Franklin× Yerong
tfy1.2-1	Leaf chlorosis	Waterlogging	3H	MQTL3.3	7.31	36	69	64.66727053	73.33272947	Haobing-Li-etal-2008	Franklin× TX9425
tfy2.1-1	Leaf chlorosis	Waterlogging	3H	MQTL3.3	9.28	34.1	70.5	65.92585745	75.07414255	Haobing-Li-etal-2008	Franklin× TX9425

Continued Table S1.

QTL names	Traits	Stress	Chr.	MQTL	LOD score	Phenotypic variance	Peak position	From	To	Reference	Parents
SLw2.3	Spike length	Waterlogging	3H	MQTL3.4	7.74	14.37	93	75.16771051	110.8322895	Da-wei-XUE-et_al-_2010	Yerong× Franklin
Stagnant Tiller	Tiller number	Waterlogging	4H	MQTL4.2	4.8	20	6.8	1.105555556	12.49444444	Sue-Broughton-etal-2015	Franklin× YYXT
Aerated Porosity	Porosity	Waterlogging	4H	MQTL4.7	12.1	20	116	110.3055556 110.3055556	121.6944444	Sue-Broughton-etal-2015	Franklin× YYXT
Stagnant Porosity	Porosity	Waterlogging	4H	MQTL4.7	13.52	20	116	110.3055556	121.6944444	Sue-Broughton-etal-2015	Franklin× YYXT
QWl.YyFr.4H	waterlogging score	Waterlogging	4H	MQTL4.7	4.1	5.2	121.1	105.0557245	137.1442755	Meixue-Zhou-2012	Erleng× Franklin
tfmas	Plant biomass	Waterlogging	4H	MQTL4.7	2.75	16.3	62.5	52.93078154	72.06921846	Haobing-Li-etal-2008	Franklin× TX9425
yfy2.1-3	Leaf yellowing	Waterlogging	4H	MQTL4.8	9.25	18.5	108	10.36176515	112.3823485	Haobing-Li-etal-2008	Franklin× Yerong
yfy2.2-3	Leaf yellowing	Waterlogging	4H	MQTL4.8	10.37	22.4	109	105.3806497	112.6193503	Haobing-Li-etal-2008	Franklin× Yerong
yfmas	Reduction of plant biomass	Waterlogging	4H	MQTL4.8	3.03	8.2	105.5	95.61299435	115.3870056	Haobing-Li-etal-2008	Franklin× Yerong
yfsur-2	Plant survival	Waterlogging	5H	MQTL5.6	5.05	13.1	50	43.8111873	56.1888127	Haobing-Li-etal-2008	Franklin× Yerong
yfy1.1-2	Leaf yellowing	Waterlogging	5H	MQTL5.6	3.94	7.6	45.5	34.83244127	56.16755873	Haobing-Li-etal-2008	Franklin× Yerong
GSw2.3	Grains per spike	Waterlogging	5H	MQTL5.7	6.18	10.02	58.85	33.2761477	84.4238523	Da-wei-XUE-et_al-_2010	Yerong× Franklin
PHw2.1	Plant height	Waterlogging	6H	MQTL6.1	5.52	9.74	38.6	12.29096509	64.90903491	Da-wei-XUE-et_al-_2010	Yerong× Franklin
QWl.YyFr.6H	Waterlogging score	Waterlogging	6H	MQTL6.3	3.57	4.5	78.4	59.85994832	96.94005168	Meixue-Zhou-2012	Erleng× Franklin
GYw1.2	Grain yield	Waterlogging	7H	MQTL7.1	7.45	30.43	39.15	30.72903385	47.57096615	Da-wei-XUE-et_al-_2010	Yerong× Franklin

Continued Table S1.

QTL names	Traits	Stress	Chr.	MQTL	LOD score	Phenotypic variance	Peak position	From	To	Reference	Parents
GSw1.3	Grains number per spike	Waterlogging	7H	MQTL7.1	7.35	9.06	46.6	18.31633554	74.88366446	Da-wei-XUE-et_al-_2010	Yerong× Franklin
tfy2.1-2	Leaf chlorosis	Waterlogging	7H	MQTL7.3	3.62	16	85	75.2513587	94.7486413	Haobing-Li-etal-2008	Franklin× TX9425
yfy2.1-1	Leaf yellowing	Waterlogging	7H	MQTL7.4	3.72	6.7	68.5	56.39948562	80.60051438	Haobing-Li-etal-2008	Franklin× Yerong
SPw1.1	Spikes per plant	Waterlogging	7H	MQTL7.9	3.31	8.13	138.95	107.4309348	170.4690652	Da-wei-XUE-et_al-_2010	Yerong× Franklin
QGY-T.CmGa.1H	Grain yield	Salinity	1H	MQTL1.1	3.95	12.5	11	0.37037037	21.62962963	Xiaohui-Liu-2017	CM72× Gairdner
qPHE-1	Phenol	Salinity	1H	MQTL1.2	2.792	11.7	126	119.4284793	132.5715207	QTL-Makhtoum-etal-2019	Badia× Kavir
qPHE-1	Phenol	Salinity	1H	MQTL1.2	2.792	11.7	126	119.4284793	132.5715207	QTL-Makhtoum-etal-2019	Badia× Kavir
qPHE-1	Phenol	Salinity	1H	MQTL1.2	5.369	21.3	34	30.39029143	37.60970857	QTL-Makhtoum-etal-2019	Badia× Kavir
qSCS-1	Salinity score	Salinity	1H	MQTL1.2	3.872	15.9	126	121.1643527	130.8356473	Makhtoum-etal-2021	Kavir ×Badia
qRLS-1	Root length	Salinity	1H	MQTL1.2	2.725	11.5	126	119.314192	132.685808	Makhtoum-etal-2021	Kavir ×Badia
qSLS-1a	Stomata length	Salinity	1H	MQTL1.3	3.313	14	28	22.50808625	33.49191375	Makhtoum-etal-2021	Kavir ×Badia
qSLS-1b	Stomata length	Salinity	1H	MQTL1.3	2.689	11.5	108	101.314192	114.685808	Makhtoum-etal-2021	Kavir ×Badia
qLNS-1	Leaf number	Salinity	1H	MQTL1.3	2.975	12.5	28	21.8490566	34.1509434	Makhtoum-etal-2021	Kavir ×Badia
qSUG-1	Suger content	Salinity	1H	MQTL1.3	2.808	11.80	26	19.48417013	32.51582987	QTL-Makhtoum-etal-2019	Badia× Kavir
qSUG-1	Suger content	Salinity	1H	MQTL1.3	2.808	11.80	26	19.48417013	32.51582987	QTL-Makhtoum-etal-2019	Badia× Kavir

Continued Table S1.

QTL names	Traits	Stress	Chr.	MQTL	LOD score	Phenotypic variance	Peak position	From	To	Reference	Parents
qSUG-1	Suger content	Salinity	1H	MQTL1.3	2.682	11.3	14	7.195859075	20.80414093	QTL-Makhtoum-etal-2019	Badia× Kavir
qSUG-1	Suger content	Salinity	1H	MQTL1.3	2.868	12	26.2	19.7927673	32.6072327	QTL-Makhtoum-etal-2019	Badia× Kavir
QSl.YyFr.1H	Salinity score	salinity	1H	MQTL1.3	5.01	6.6	59	46.3590556	71.64094433	Gaofeng-Zhou-2011	YYXT× Franklin
qASL12-1	Radicle length	Salinity	1H	MQTL1.3	2.893	12.5	76	54.8	97.2	Ghaffari-Moghaddam-etal-2019	Badia× Comino
qPPT-1	Spike diameter	Salinity	1H	MQTL1.3	3.566	14.7	28	22.76960596	33.23039404	Makhtoum-etal-2021	Kavir× Badia
qPER-1	Peroxidas	Salinity	1H	MQTL1.4	5.037	20.2	82	78.19372315	85.80627685	QTL-Makhtoum-etal-2019	Badia× Kavir
qSTI (SDW)-1	Shoot dry weight	Salinity	1H	MQTL1.5	3	6	137.8	100.9944444	174.6055556	Hanen-Sbei-etal-2014	Natural population
qLIS-1	leaf injury score	Salinity	1H	MQTL1.5	3	5	140.5	96.33333333	184.6666667	Hanen-Sbei-etal-2014	Natural population
QSA-T.CmGa.1H	Stomatal pore area	Salinity	1H	MQTL1.6	3.12	12.5	121.4	110.7703704	132.0296296	Xiaohui-Liu-2017	CM72× Gairdner
qSPP1s	Spikes per plant	Salinity	1H	MQTL1.6	5.82	9.38	81.7	65.24999198	98.15000802	Dawei-Xue-2009	CM72× Gairdner
qSLAV-2	Salinity tolerance score	Salinity	2H	MQTL2.1	3	0.046	25.7	0	53.66538624	Yun-Fan-2016	Natural population
qSLAV-2	Salinity tolerance score	Salinity	2H	MQTL2.1	3	0.059	3.5	0	25.30352147	Yun-Fan-2016	Natural population
qSLAV-2	Salinity tolerance score	Salinity	2H	MQTL2.1	3	0.056	3.5	0	25.97156727	Yun-Fan-2016	Natural population
qSLAV-2	Salinity tolerance score	Salinity	2H	MQTL2.1	3	0.056	5	0	27.97156727	Yun-Fan-2016	Natural population
qSLAV-2	Salinity tolerance score	Salinity	2H	MQTL2.1	3	0.056	5.3	0	27.97156727	Yun-Fan-2016	Natural population

Continued Table S1.

QTL names	Traits	Stress	Chr.	MQTL	LOD score	Phenotypic variance	Peak position	From	To	Reference	Parents
qSLAV-2	Salinity tolerance score	Salinity	2H	MQTL2.1	3	0.056	25.7	2.728432	48.67156727	Yun-Fan-2016	Natural population
qSLAV-2	Salinity tolerance score	Salinity	2H	MQTL2.1	3	0.049	25.7	0	51.95321973	Yun-Fan-2016	Natural population
qSLAV-2	Salinity tolerance score	Salinity	2H	MQTL2.1	3.66	4.8	3.5	0	30.30016181	Yun-Fan-2016	Natural population
qSLAV-2	Salinity tolerance score	Salinity	2H	MQTL2.1	2.11	2.6	25.7	0	75.17722181	Yun-Fan-2016	Natural population
qSWt-2	Shoot weight	Salinity	2H	MQTL2.1	2	5	0	0	19.3974359	R.P.Ellis-etal-2001	Derkado× B83-12/21/5
qGS2-2	Growth stage	Salinity	2H	MQTL2.1	5.4	8	23	0	34.49839744	R.P.Ellis-etal-2001	Derkado× B83-12/21/5
QBM-T.CmGa.2H	Biomass	Salinity	2H	MQTL2.1	3.49	14	6.8	0	116.29074074	Xiaohui-Liu-2017	CM72× Gairdner
QTR-T.C-2	TR transpiration rate	Salinity	2H	MQTL2.1	2.54	10.4	6.8	0	19.57599715	Xiaohui-Liu-2017	CM72× Gairdner
QGS-T-2	Stomatal conductance	Salinity	2H	MQTL2.1	2.54	10.3	10.3	0	23.20003596	Xiaohui-Liu-2017	CM72× Gairdner
qLWS-2	Leaf weight	Salinity	2H	MQTL2.2	2.651	11.2	4	0	10.86489218	Makhtoum-etal-2021	Kavir× Badia
qSUG2-a	Suger content	Salinity	2H	MQTL2.2	3.323	13.8	6	0.428493301	11.5715067	QTL-Makhtoum-etal-2019	Badia× Kavir
qSUG2-b	Suger content	Salinity	2H	MQTL2.2	4.622	18.7	32	27.88840682	36.11159318	QTL-Makhtoum-etal-2019	Badia× Kavir
qFLL-2	length of flag leaf	Salinity	2H	MQTL2.2	2.077	9.1	6	0	35.12087912	Ghaffari-Moghaddam-etal-1397	Badia× comino
QSl.YyFr.2H	Salinity tolerance score	Salinity	2H	MQTL2.2	7.64	10.6	48	40.12922334	55.87077666	Gaofeng-Zhou-2011	YYXT× Franklin
qSDW15- 2	Dry weight of grains	Salinity	2H	MQTL2.2	2.28	10	12	0	38.5	Ghaffari-Moghaddam-etal-2019	Badia× Comino

Continued Table S1.

QTL names	Traits	Stress	Chr.	MQTL	LOD score	Phenotypic variance	Peak position	From	To	Reference	Parents
qASL4-2a	Radicle length	Salinity	2H	MQTL2.2	2.629	11.4	16	0	39.22561404	Ghaffari-Moghaddam-etal-2019	Badia× Comino
qSV4-2a	Seed vigor	Salinity	2H	MQTL2.4	2.389	10.4	64	38.51923077	89.48076923	Ghaffari-Moghaddam-etal-2019	Badia× Comino
qCAT-2	Catalase	Salinity	2H	MQTL2.5	3.608	14.9	58.1	52.93981259	63.26018741	QTL-Makhtoum-etal-2019	Badia× Kavir
qDWP2s	Dry weight per plant	Salinity	2H	MQTL2.5	2.85	10.75	326.3	311.9464116	340.6535884	Dawei-Xue-2009	CM72× Gairdner
qGNP2s	Grain number per plant	Salinity	2H	MQTL2.5	6.83	25.33	340.3	334.2083666	346.3916334	Dawei-Xue-2009	CM72× Gairdner
qFLW-2	Flag leaf width	Salinity	2H	MQTL2.5	3.462	14.3	60	54.62330123	65.37669877	Makhtoum-etal-2021	Kavir× Badia
QLT-T.CmGa.2H	Leaf temperature	Salinity	2H	MQTL2.6	3.09	11.2	112.8	100.9365741	124.6634259	Xiaohui-Liu-2017	CM72× Gairdner
QLT-T.CmGa.2H	Leaf temperature	Salinity	2H	MQTL2.6	4.19	16.5	112.8	104.7472503	120.8527497	Xiaohui-Liu-2017	CM72× Gairdner
QGY-C.CmGa.2H	Grain yield	Salinity	2H	MQTL2.7	3.77	15	134	125.1419753	142.8580247	Xiaohui-Liu-2017	CM72× Gairdner
qVPD-T/C-2	Leaf vapour pressure	Salinity	2H	MQTL2.7	2.63	10.7	153.6	141.1822084	166.0177916	Xiaohui-Liu-2017	CM72× Gairdner
qARL20- 2a	Radicle length	Salinity	2H	MQTL2.7	3.622	15.4	112	94.79220779	129.2077922	Ghaffari-Moghaddam-etal-2019	Badia× Comino
qSFW4-2a	Shoot fresh weight	Salinity	2H	MQTL2.7	2.268	9.9	114	87.23232323	140.7676768	Ghaffari-Moghaddam-etal-2019	Badia× Comino
qARL15- 2a	Radicle length	Salinity	2H	MQTL2.7	2.43	10.6	112	87	137	Ghaffari-Moghaddam-etal-2019	Badia× Comino
qPEO-2	Phenol	Salinity	2H	MQTL2.8	3.05	12.7	102.5	96.44592185	108.5540781	QTL-Makhtoum-etal-2019	Badia× Kavir
qPHE-2	Phenol	Salinity	2H	MQTL2.8	7.704	29.1	102	99.35784218	104.6421578	QTL-Makhtoum-etal-2019	Badia× Kavir

Continued Table S1.

QTL names	Traits	Stress	Chr.	MQTL	LOD score	Phenotypic variance	Peak position	From	To	Reference	Parents
QSl.TxNn.2H	Salinity score	salinity	2H	MQTL2.8	24.37	45	14.7	11.23073286	18.16926714	Rugen-Xu-2012	TX9425× Naso Nijo
RDW/P	Dry weight of roots	Salinity Waterlogging	2H	MQTL2.8	4.11	9.6	16.1	0	32.36218972	Rugen-Xu-2012	TX9425× Naso Nijo
PHS/C	Plant height	salinity	2H	MQTL2.9	3.26	7.7	22.9	2.625062172	43.17493783	Rugen-Xu-2012	TX9425× Naso Nijo
GSDWS	Shoots dry weight	salinity	2H	MQTL2.9	9.17	18.1	23.1	14.47475021	31.72524979	Rugen-Xu-2012	TX9425× Naso Nijo
NoTS	Number of tiller	salinity	2H	MQTL2.9	3.12	7.3	23.1	1.714106674	44.48589333	Rugen-Xu-2012	TX9425× Naso Nijo
PHS	Plant height	salinity	2H	MQTL2.9	4.86	9.6	22.9	6.637810284	39.16218972	Rugen-Xu-2012	TX9425× Naso Nijo
GSFWS	Shoots dry weight	salinity	2H	MQTL2.9	4.39	10.5	22.9	8.031712259	37.76828774	Rugen-Xu-2012	TX9425× Naso Nijo
RFW/P	fresh weight of roots	Salinity Waterlogging	2H	MQTL2.9	4.58	10.6	22.9	8.171979125	37.62802088	Rugen-Xu-2012	TX9425× Naso Nijo
qPm.SEVAS-3H	Severity for powdery Medlow in salinity stress	salinity	3H	MQTL3.1	3.0242	18.8	59.77	55.610277	63.789723	Makhtoum-etal-2021	Badia× Kavir
QPC-S.TxFr.3H	Proline content	salinity	3H	MQTL3.1	3.22	18.6	74.8	64.08464755	85.51535245	Yun-Fan-2015	TX9425× Franklin
qPER-3	Peroxidas	salinity	3H	MQTL3.2	3.35	13.9	44	38.46857608	49.53142392	QTL-Makhtoum-etal-2019	Badia× Kavir
qPER-3	Peroxidas	salinity	3H	MQTL3.2	3.35	13.9	44	38.46857608	49.53142392	QTL-Makhtoum-etal-2019	Badia× Kavir
qPER-3a	Peroxidas	salinity	3H	MQTL3.2	3.57	14.8	44	38.80494646	49.19505354	QTL-Makhtoum-etal-2019	Badia× Kavir
qPER-3b	Peroxidas	salinity	3H	MQTL3.2	4.902	19.7	66	62.09711713	69.90288287	QTL-Makhtoum-etal-2019	Badia× Kavir
qPER-3c	Peroxidas	salinity	3H	MQTL3.2	3.565	14.7	150	144.769606	155.230394	QTL-Makhtoum-etal-2019	Badia× Kavir

Continued Table S1.

QTL names	Traits	Stress	Chr.	MQTL	LOD score	Phenotypic variance	Peak position	From	To	Reference	Parents
qPER-3	Peroxidas	Salinity	3H	MQTL3.2	3.468	14.4	44.2	38.86063941	49.53936059	QTL-Makhtoum-etal-2019	Badia× Kavir
qSPP3s	Spikes per plant	Salinity	3H	MQTL3.2	2.96	3.97	429.7	390.8332304	468.5667696	Dawei-Xue-2009	CM72× Gairdner
qLWS-3	Leaf weight	Salinity	3H	MQTL3.2	2.995	12.4	44	37.79945222	50.20054778	Makhtoum-etal-2021	Kavir× Badia
QGY-T.CmGa.3H	Grain yield	salinity tolerance	3H	MQTL3.3	4.35	13.9	58.9	49.34098055	68.45901945	Xiaohui-Liu-2017	CM72× Gairdner
QGY-T/C.CmGa.3H	Grain yield	salinity tolerance	3H	MQTL3.3	4.42	17.3	58.9	51.21963177	66.58036823	Xiaohui-Liu-2017	CM72× Gairdner
QSTC.CmGa.3H	Salinity tolerance score	salinity tolerance	3H	MQTL3.3	4.29	16.8	60.1	52.19104938	68.00895062	Xiaohui-Liu-2017	CM72× Gairdner
q AWL-T-3	AWL width/length	salinity tolerance	3H	MQTL3.5	2.78	11.3	136.9	125.14156017	148.6584399	Xiaohui-Liu-2017	CM72× Gairdner
qPH3s	Plant height	Salinity	3H	MQTL3.5	5.17	14.15	109.9	98.99533037	120.8046696	Dawei-Xue-2009	CM72× Gairdner
q SALV-3	Salinity tolerance score	salinity tolerance	3H	MQTL3.5	3	0.028	35.7	0	81.64313454	Yun-Fan-2016	Natural population
q SALV-3	Salinity tolerance score	salinity tolerance	3H	MQTL3.5	3	0.036	20	0	55.73354908	Yun-Fan-2016	Natural population
q SALV-3	Salinity tolerance score	salinity tolerance	3H	MQTL3.5	3	0.032	97.4	57.19975728	137.6002427	Yun-Fan-2016	Natural population
q SALV-3	Salinity tolerance score	salinity tolerance	3H	MQTL3.5	3	0.033	145.5	106.5179465	184.4820535	Yun-Fan-2016	Natural population
q SALV-3	Salinity tolerance score	salinity tolerance	3H	MQTL3.5	3	0.032	145.5	105.2997573	185.7002427	Yun-Fan-2016	Natural population
q SALV-3	Salinity tolerance score	salinity tolerance	3H	MQTL3.5	2.12	2.7	133.5	85.8552679	181.1447321	Yun-Fan-2016	Natural population
qLIS-3	Leaf injury score	salt tolerance	3H	MQTL3.6	3	8	91.9	64.29583333	119.5041667	Hanen-Sbei-etal-2014	Natural population

Continued Table S1.

QTL names	Traits	Stress	Chr.	MQTL	LOD score	Phenotypic variance	Peak position	From	To	Reference	Parents
qLEL-3	Leaf length	Salinity	3H	MQTL3.6	2.87	12	132	125.5927673	138.4072327	Makhtoum-etal-2021	Kavir× Badia
qLIS-4	leaf injury score	salt tolerance	4H	MQTL4.1	3	4	65.1	9.891666667	120.308333	Hanen-Sbei-etal-2014	Natural population
qSAL-4	Salt tolerance at germination	salt tolerance	4H	MQTL4.1	5.1	0.147	0.25	0	6.801613934	Yoshiro-1996	Steptoe× Morex
qSWt-4	Shoots weights	salinity tolerance	4H	MQTL4.1	4.4	7	18	4.858974359	31.14102564	R.P.Ellis-etal-2001	Derkado× B83-12/21/5
qGN-4	Grain Number	salinity tolerance	4H	MQTL4.1	8.1	5	6	0	24.3974359	R.P.Ellis-etal-2001	Derkado× B83-12/21/5
qPY-4	Grain yield	salinity tolerance	4H	MQTL4.1	9.7	5	3	0	21.3974359	R.P.Ellis-etal-2001	Derkado× B83-12/21/5
qRLS-4	Root length	Salinity	4H	MQTL4.2	2.836	11.9	140	133.538925	146.461075	Makhtoum-etal-2021	Kavir× Badia
qSUG-4	Suger content	Salinity	4H	MQTL4.3	5.045	20.2	118	114.1937231	121.8062769	QTL-Makhtoum-etal-2019	Badia× Kavir
qSWS-4	Stomata width	Salinity	4H	MQTL4.4	2.863	12.2	28	21.6978039	34.3021961	Makhtoum-etal-2021	Kavir× Badia
qSPP4s	Spikes per plant	Salinity	4H	MQTL4.5	3.85	5.84	368.8	342.378583	395.221417	Dawei-Xue-2009	CM72× Gairdner
qSPL4s	Spikes per line	Salinity	4H	MQTL4.5	10.57	27.30	337.2	331.547946	342.852054	Dawei-Xue-2009	CM72× Gairdner
qSHT-4	Shoot diameter	Salinity	4H	MQTL4.5	3.094	12.9	50	44.03978353	55.96021647	Makhtoum-etal-2021	Kavir× Badia
qPHE-4a	Phenol	Salinity	4H	MQTL4.5	2.544	10.8	42	34.88085255	49.11914745	QTL-Makhtoum-etal-2019	Badia× Kavir
qPHE-4b	Phenol	Salinity	4H	MQTL4.5	4.631	18.7	118	113.884068	122.1115932	QTL-Makhtoum-etal-2019	Badia× Kavir
qTI4s	Tiller number	Salinity	4H	MQTL4.6	2.78	4.48	337.2	302.7577957	3716422043	Dawei-Xue-2009	CM72× Gairdner

Continued Table S1.

QTL names	Traits	Stress	Chr.	MQTL	LOD score	Phenotypic variance	Peak position	From	To	Reference	Parents
qRFW15- 4a	Fresh weight root	Salinity	4H	MQTL4.6	2.499	10.9	26	1.688073394	50.31192661	Ghaffari-Moghaddam-etal-2019	Badia× Comino
qSDW-4a	Fresh weight shoot	Salinity	4H	MQTL4.6	2.25	9.8	118	90.95918367	145.0408163	Ghaffari-Moghaddam-etal-2019	Badia× Comino
qSFW15- 4	Fresh weight shoot	Salinity	4H	MQTL4.6	2.132	9.4	40	11.80851064	68.19148936	Ghaffari-Moghaddam-etal-2019	Badia× Comino
qLWS-4a	Leaf weight	Salinity	4H	MQTL4.6	5.212	20.8	56	52.30351959	59.69648041	Makhtoum-etal-2021	Kavir× Badia
qLWS-4b	Leaf weight	Salinity	4H	MQTL4.6	3.169	14.9	140	134.8398126	145.1601874	Makhtoum-etal-2021	Kavir× Badia
qSLS-4	Stomata length	Salinity	4H	MQTL4.6	5.145	20.9	58	54.3212061	61.6787939	Makhtoum-etal-2021	Kavir× Badia
qLNS-4	Leaf number	Salinity	4H	MQTL4.6	3.909	1.6	56	7.945754717	104.0542453	Makhtoum-etal-2021	Kavir× Badia
qSCS-4	Salinity score	Salinity	4H	MQTL4.6	2.851	1.2	56	0	120.072327	Makhtoum-etal-2021	Kavir× Badia
qLEN-4	Leaf number	Salinity	4H	MQTL4.6	2.721	11.5	58	51.31419196	64.68580804	Makhtoum-etal-2021	Kavir× Badia
qPER-4	Peroxidas	Salinity	4H	MQTL4.6	3.333	13.8	60	54.4284933	65.5715067	QTL-Makhtoum-etal-2019	Badia× Kavir
qPER-4	Peroxidas	Salinity	4H	MQTL4.6	3.333	13.8	60	54.4284933	65.05715067	QTL-Makhtoum-etal-2019	Badia× Kavir
qPER-4a	Peroxidas	Salinity	4H	MQTL4.6	2.718	11.4	60	53.25554452	66.74445548	QTL-Makhtoum-etal-2019	Badia× Kavir
qPER-4b	Peroxidas	Salinity	4H	MQTL4.6	6.399	24.9	118	114.912177	121.087823	QTL-Makhtoum-etal-2019	Badia× Kavir
qLEL-4	Leaf length	Salinity	4H	MQTL4.6	3.223	13.4	58	52.26217967	63.73782033	Makhtoum-etal-2021	Kavir× Badia
qTLW-4	Total weight of leaves	Salinity	4H	MQTL4.7	3.354	14.3	76	57.46853147	94.53146853	Ghaffari-Moghaddam-etal-1397	Badia× comino

Continued Table S1.

QTL names	Traits	Stress	Chr.	MQTL	LOD score	Phenotypic variance	Peak position	From	To	Reference	Parents
qSDW12- 4a	Dry weight of seeds	Salinity	4H	MQTL4.8	2.422	10.6	122	97	147	Ghaffari-Moghaddam-etal-2019	Badia× Comino
q SALV-4	Salinity tolerance score	Salinity	4H	MQTL4.8	3	0.031	145.5	104.002975	186.9970247	Yun-Fan-2016	Natural population
qSLAV-4	Salinity tolerance score	Salinity	4H	MQTL4.8	3	0.038	145	111.147164	178.852836	Yun-Fan-2016	Natural population
qSLAV-4	Salinity tolerance score	Salinity	4H	MQTL4.8	3	0.049	145.1	118.7467803	171.2532197	Yun-Fan-2016	Natural population
qSLAV-4	Salinity tolerance score	Salinity	4H	MQTL4.8	3	0.041	145.1	113.7242008	176.4757992	Yun-Fan-2016	Natural population
qSLAV-4	Salinity tolerance score	Salinity	4H	MQTL4.8	3	0.043	145	115.0835403	174.9164597	Yun-Fan-2016	Natural population
qSLAV-4	Salinity tolerance score	Salinity	4H	MQTL4.8	5.67	7.5	145	127.8748964	162.1521036	Yun-Fan-2016	Natural population
qFSW-4	Seedling fresh weight	Salinity	4H	MQTL4.8	2.896	12.5	76	54.8	97.2	Ghaffari-Moghaddam-etal-1397	Badia× comino
qTNL-4	Total number of leaves	Salinity	4H	MQTL4.8	2.084	9.1	118	88.87912088	147.1208791	Ghaffari-Moghaddam-etal-1397	Badia× comino
qSD-4	Stem diameter	Salinity	4H	MQTL4.8	2.515	10.9	126	101.6880734	150.3119266	Ghaffari-Moghaddam-etal-1397	Badia× comino
qSAL-5	Salinity tolerance score at germination	Salinity	5H	MQTL5.1	19.4	0.467	10.9	8.837714672	12.9628533	Yoshiro-1996	Steptoe× Morex
qSAL-5	Salinity tolerance score at germination	Salinity	5H	MQTL5.1	5.5	0.174	17.9	12.36501581	23.43498419	Yoshiro-1996	Harrington× TR 306
qSLAV-5	Salinity tolerance score	Salinity	5H	MQTL5.2	3	0.040	145.1	115.0835403	174.9164597	Yun-Fan-2016	Natural population
qSLAV-5	Salinity tolerance score	Salinity	5H	MQTL5.2	3	0.030	43.5	0.6197411	86.3802589	Yun-Fan-2016	Natural population
qSLAV-5	Salinity tolerance score	Salinity	5H	MQTL5.2	3	0.039	97.9	64.91518546	130.8848145	Yun-Fan-2016	Natural population

Continued Table S1.

QTL names	Traits	Stress	Chr.	MQTL	LOD score	Phenotypic variance	Peak position	From	To	Reference	Parents
qSLAV-5	Salinity tolerance score	Salinity	5H	MQTL5.2	3	0.044	98.2	68.96345984	127.4365402	Yun-Fan-2016	Natural population
qSLAV-5	Salinity tolerance score	Salinity	5H	MQTL5.2	3	0.035	166.1	129.3454924	202.8545076	Yun-Fan-2016	Natural population
qSLAV-5	Salinity tolerance score	Salinity	5H	MQTL5.2	3	0.035	168.3	131.5454924	205.0545076	Yun-Fan-2016	Natural population
qSLAV-5	Salinity tolerance score	Salinity	5H	MQTL5.2	3	0.035	168.3	131.5454924	205.0545076	Yun-Fan-2016	Natural population
qSLAV-5	Salinity tolerance score	Salinity	5H	MQTL5.2	3.91	5.1	43.5	18.27631829	68.72368171	Yun-Fan-2016	Natural population
qSGa-5	Seedling gibberellic acid content	Salinity	5H	MQTL5.3	5.7	6	0	0	16.33119658	R.P.Ellis-etal-2001	Derkado× B83-12/21/5
qPY-5	Grain yield	Salinity	5H	MQTL5.3	15.6	7	0	0	14.14102564	R.P.Ellis-etal-2001	Derkado× B83-12/21/5
qSδC-5	Shoot δ	Salinity	5H	MQTL5.3	10.3	15	2	0	8.132478632	R.P.Ellis-etal-2001	Derkado× B83-12/21/5
qSW_{tI}-5	Reduced shoot	Salinity	5H	MQTL5.3	6.6	10	1	0	10.19871795	R.P.Ellis-etal-2001	Derkado× B83-12/21/5
qRW_{t}-5	Root dry weight	Salinity	5H	MQTL5.3	7	20	1	0	5.599358974	R.P.Ellis-etal-2001	Derkado× B83-12/21/5
qSHT-5	Shoot diameter	Salinity	5H	MQTL5.4	2.608	11	42	35.0102916	48.9897084	Makhtoum-etal-2021	Kavir× Badia
qPm.SEVAS-5H	Severity for powdery Medlow in salinity stress	Salinity	5H	MQTL5.4	3.183	13.3	112	106.2190382	117.7809618	Makhtoum-etal-2021	Badia× Kavir
qLIS-5	Leaf injury score	Salinity	5H	MQTL5.8	3	4	89.4	34.19166667	144.6083333	Hanen-Sbei-etal-2014	Natural population
qLEL-5	Leaf length	Salinity	5H	MQTL5.9	2.526	10.7	138	130.8143185	145.1856815	Makhtoum-etal-2021	Kavir× Badia
qLFW-5	Leaf weight	Salinity	5H	MQTL5.9	2.566	10.8	138	130.8808526	145.1191474	Makhtoum-etal-2021	Kavir× Badia

Continued Table S1.

QTL names	Traits	Stress	Chr.	MQTL	LOD score	Phenotypic variance	Peak position	From	To	Reference	Parents

qFLW-5	Flag leaf weight	Salinity	5H	MQTL5.9	3.3	13.7	138	132.3878254	143.6121746	Makhtoum-etal-2021	Kavir× Badia
qFLW-5	Flag leaf weight	Salinity	5H	MQTL5.9	4.699	19	138	133.9533267	142.0466733	Makhtoum-etal-2021	Kavir× Badia
qSUG-5	Suger content	Salinity	5H	MQTL5.9	2.526	10.7	132	131.86	132.14	QTL-Makhtoum-etal-2019	Badia× Kavir
qNAK6s	Na^+:K^+ ratio	Salinity	6H	MQTL6.1	6.1	29.81	485.8	480.6238485	490.9761515	Dawei-Xue-2009	CM72× Gairdner
qGY6s	Grain yield	Salinity	6H	MQTL6.1	3.02	6.21	493.6	468.7528059	518.4471941	Dawei-Xue-2009	CM72× Gairdner
QSl.YyFr.6H	Salinity tolerance score	Salinity	6H	MQTL6.1	4.71	6.8	26	13.7308415	38.26915185	Gaofeng-Zhou-2011	YYXT× Franklin
qSAL-6	Salt tolerance at germination	Salinity	6H	MQTL6.2	2.2	0.068	5.7	0	19.86304777	Yoshiro-1996	Steptoe× Morex
qSHT-6a	Shoot diameter	Salinity	6H	MQTL6.3	2.69	11.3	62	55.19585907	68.80414093	Makhtoum-etal-2021	Kavir× Badia
qSHT-6b	Shoot diameter	Salinity	6H	MQTL6.3	4.328	17.6	74	69.63143225	78.36856775	Makhtoum-etal-2021	Kavir× Badia
qLWS-6	Leaf weight	Salinity	6H	MQTL6.3	2.754	11.6	74	67.37182824	80.62817176	Makhtoum-etal-2021	Kavir× Badia
qLNS-6	Leaf number	Salinity	6H	MQTL6.3	2.794	11.7	62	55.42847928	68.57152072	Makhtoum-etal-2021	Kavir× Badia
qSCS-6a	Salinity tolerance score	Salinity	6H	MQTL6.3	2.695	11.4	62	55.25554452	68.74445548	Makhtoum-etal-2021	Kavir× Badia
qSCS-6b	Salinity tolerance score	Salinity	6H	MQTL6.3	2.705	11.4	74	67.25554452	80.74445548	Makhtoum-etal-2021	Kavir× Badia
qSLAV-6	Salinity tolerance score	Salinity	6H	MQTL6.3	3	0.029	173.7	129.3411115	218.0588885	Yun-Fan-2016	Natural population
qSLAV-6	Salinity tolerance score	Salinity	6H	MQTL6.3	3	0.029	38	0	82.3588852	Yun-Fan-2016	Natural population

Continued Table S1.

QTL names	Traits	Stress	Chr.	MQTL	LOD score	Phenotypic variance	Peak position	From	To	Reference	Parents

qSLAV-6	Salinity tolerance score	Salinity	6H	MQTL6.3	3	0.036	68.2	32.46645092	103.9335491	Yun-Fan-2016	Natural population
qFLDW-6	Dry weight of flag leaves	Salinity	6H	MQTL6.4	2.173	9.5	32	4.105263158	59.89473684	Ghaffari-Moghaddam-etal-1397	Badia× comino
qPPT-6	Spike diameter	Salinity	6H	MQTL6.4	3.086	12.9	62	56.03978353	67.96021647	Makhtoum-etal-2021	Kavir× Badia
qLEN-6a	Leaf number	Salinity	6H	MQTL6.4	4.512	18.3	42	37.79853593	46.20146407	Makhtoum-etal-2021	Kavir× Badia
qLEN-6b	Leaf number	Salinity	6H	MQTL6.4	2.571	10.9	70	62.94616583	77.05383417	Makhtoum-etal-2021	Kavir× Badia
qLFW-6	Leaf weight	Salinity	6H	MQTL6.4	2.521	10.7	98	90.81431846	105.1856815	Makhtoum-etal-2021	Kavir× Badia
qFLW-6	Flag leaf weight	Salinity	6H	MQTL6.4	3.295	13.7	98	92.38782537	103.6121746	Makhtoum-etal-2021	Kavir× Badia
qLEW-6	Leaf width	Salinity	6H	MQTL6.4	2.949	12.4	112	105.7994522	118.2005478	Makhtoum-etal-2021	Kavir× Badia
qAWW-6	Awn weight	Salinity	6H	MQTL6.4	2.804	11.8	112	105.4841701	118.5158299	Makhtoum-etal-2021	Kavir× Badia
qLEL-6	Leaf length	Salinity	6H	MQTL6.4	2.669	11.2	98	91.13510782	104.8648922	Makhtoum-etal-2021	Kavir× Badia
QTL5	Seminal roots	Salinity	7H	MQTL7.1	15.05	45.3	17.8	11.71949153	23.88050847	Wenta-Xue-2014	Tremois× Nure
QTL5	Seminal roots	Salinity	7H	MQTL7.1	14.94	45.0	17.8	11.71949153	23.88050847	Wenta-Xue-2014	Tremois× Nure
QTL5	Seminal roots	Salinity	7H	MQTL7.1	14.58	44.2	17.2	11.11949153	23.28050847	Wenta-Xue-2014	Tremois× Nure
QTL5	Seminal roots	Salinity	7H	MQTL7.1	13.77	42.4	17.2	11.11949153	23.28050847	Wenta-Xue-2014	Tremois× Nure
QTL5	Seminal roots	Salinity	7H	MQTL7.1	12.19	38.6	17.2	11.11949153	23.28050847	Wenta-Xue-2014	Tremois× Nure

Continued Table S1.

QTL names	Traits	Stress	Chr.	MQTL	LOD score	Phenotypic variance	Peak position	From	To	Reference	Parents
qSLAV-7	Salinity tolerance score	Salinity	7H	MQTL7.1	3	0.031	140.9	99.40297526	182.3970247	Yun-Fan-2016	Natural population

qSLAV-7	Salinity tolerance score	Salinity	7H	MQTL7.1	2.13	2.6	3.5	0	52.97722181	Yun-Fan-2016	Natural population
qSLAV-7	Salinity tolerance score	Salinity	7H	MQTL7.1	2.47	3.2	125.4	85.19975728	165.6002427	Yun-Fan-2016	Natural population
qSPL7s	Spikes per line	Salinity	7H	MQTL7.1	3.36	7.52	522.1	501.58124	542.61876	Dawei-Xue-2009	CM72× Gairdner
QST.TxFr.7H	Salinity tolerance score	Salinity	7H	MQTL7.1	5.4	29.2	82.3	75.47446728	89.12553272	Yun-Fan-2015	TX9425× Franklin
qSAL-7	Shoot sodium exclusion	Salinity	7H	MQTL7.3	3	20	13.9	3.934722222	23.86527778	Yuri-2010	CPI-71284-48× Barque-73
qSAL-7	Shoot sodium exclusion	Salinity	7H	MQTL7.3	3	20	28.3	18.33472222	38.26527778	Yuri-2010	CPI-71284-48× Barque-73
qLEW-7a	Leaf width	Salinity	7H	MQTL7.5	3.468	14.4	36	30.66063941	41.33936059	Makhtoum-etal-2021	Kavir× Badia
qLEW-7b	Leaf width	Salinity	7H	MQTL7.5	2.91	12.2	94	87.6978039	100.3021961	Makhtoum-etal-2021	Kavir× Badia
qPDL-7a	Peduncle length	Salinity	7H	MQTL7.5	3.184	13.3	36	30.21903816	41.78096184	Makhtoum-etal-2021	Kavir× Badia
qPDL-7b	Peduncle length	Salinity	7H	MQTL7.5	3.02	12.6	94	87.8978361	100.1021264	Makhtoum-etal-2021	Kavir× Badia
qAWW-7a	Awn weight	Salinity	7H	MQTL7.5	3.026	12.7	36	29.94592185	42.05407815	Makhtoum-etal-2021	Kavir× Badia
qAWW-7b	Awn weight	Salinity	7H	MQTL7.5	3.067	12.8	94	87.99321934	100.0067807	Makhtoum-etal-2021	Kavir× Badia
qSLS-7	Stomata length	Salinity	7H	MQTL7.5	3.652	15.3	100	94.97471945	105.0252806	Makhtoum-etal-2021	Kavir× Badia
qPHE-7	Phenol	Salinity	7H	MQTL7.6	28.3	7.454	144	143.946	144.054	QTL-Makhtoum-etal-2019	Badia× Kavir

Continued Table S1.

QTL names	Traits	Stress	Chr.	MQTL	LOD score	Phenotypic variance	Peak position	From	To	Reference	Parents
qPLS-7	Plumule length	Salinity	7H	MQTL7.6	2.572	10.9	98	90.94616583	105.0538342	Makhtoum-etal-2021	Badia× Kavir
QSl.YyFr.7H	Salinity tolerance score	Salinity	7H	MQTL7.6	10.87	15.9	69	63.75281556	74.24718444	Gaofeng-Zhou-2011	YYXT× Franklin

qLWS-7	Leaf weight	Salinity	7H	MQTL7.6	3.483	14.4	98	92.66063941	103.3393606	Makhtoum-etal-2021	Kavir× Badia
qSCS-7	Salinity score	Salinity	7H	MQTL7.6	4.466	18.1	98	93.75211091	102.2478891	Makhtoum-etal-2021	Kavir× Badia
qPPT-7	Spike diameter	Salinity	7H	MQTL7.6	2.681	11.3	98	91.19585907	104.8041409	Makhtoum-etal-2021	Kavir× Badia
qLEL-7	Leaf length	Salinity	7H	MQTL7.8	2.958	12.4	94	87.79945222	100.2005478	Makhtoum-etal-2021	Kavir× Badia
qSPL-7	Spike length	Salinity	7H	MQTL7.9	2.899	12.2	68	61.6978039	74.3021961	Makhtoum-etal-2021	Kavir× Badia
qLNS-7a	Leaf number	Salinity	7H	MQTL7.9	4.05	16.6	62	57.36826551	66.63173449	Makhtoum-etal-2021	Kavir× Badia
qLNS-7b	Leaf number	Salinity	7H	MQTL7.9	4.468	18.1	98	93.75211091	102.2478891	Makhtoum-etal-2021	Kavir× Badia
qRLN-7a	Root length	Salinity	7H	MQTL7.9	2.533	10.8	66	58.88085255	73.11914745	Makhtoum-etal-2021	Kavir× Badia
qRLN-7b	Root length	Salinity	7H	MQTL7.9	2.605	11.1	134	127.0732619	140.9267381	Makhtoum-etal-2021	Kavir× Badia
qPER-7	Peroxidas	Salinity	7H	MQTL7.10	5.183	20.7	76	72.2856622	79.7143378	QTL-Makhtoum-etal-2019	Badia× Kavir
qSUG-7	Suger content	Salinity	7H	MQTL7.10	4.259	17.3	76	71.55567674	80.44432326	QTL-Makhtoum-etal-2019	Badia× Kavir
qRFW15- 7a	Fresh weight of root	Salinity	7H	MQTL7.10	2.22	9.7	78	50.68041237	105.3195876	Ghaffari-Moghaddam-etal-2019	Badia× Comino
qSHT-7	Shoot diameter	Salinity	7H	MQTL7.10	2.619	11.1	78	71.07326194	84.92673806	Makhtoum-etal-2021	Kavir× Badia
