# Supplementary material for: Mega Meta-QTLs: A Strategy for the Production of Golden Barley (Hordeum vulgare L.) Tolerant to Abiotic Stresses
Source: Genes (Basel). 2022 Nov 10;13(11):2087. doi: 10.3390/genes13112087 (PMC9690463; doi:10.3390/genes13112087)
Supplement: Supplementary file 1 [file genes-13-02087-s001.zip › Table S2.pdf]

Table S2. No. of original QTLs and QTLs for MQTLs

Meta-QTL	No. of original QTLs	QTLs
MQTL1.1	7	Bernhard-Saal-etal-2011-TGW-1H; F-TMC-Ap3-1H; Xiaohui-LiuX-GY-T-1H; Sue-Broughton-etal-LRL-1H
MQTL1.2	14	Makhtoum-etal-2021-SCS-1H; Sue-Broughton-etal-SDW-1H; Arifuzzaman-RSR-1H; QTL-Makhtoum-etal-2019-PHE-1H; Makhtoum-etal-2021-RLS-1H; QTL-Makhtoum-etal-2019-PHR-1H; Arifuzzaman-RDW-1H; Sue-Broughton-etal-SFW-1H
MQTL1.3	18	X.Huang-etal-2018-Sur.yf-1H; Makhtoum-etal-2021-SLS-1H; QTL-Makhtoum-etal-2019-SUG-1H; Makhtoum-etal-2021-LNS-1H; Makhtoum-etal-2021-PPT-1H; Arifuzzaman-PH-1H; Sue-Broughton-etal-RFW-1H; Sue-Broughton-etal-RDW-1H; Gaofeng-Zhou-SAL-1H; Ghaffari-Moghaddam-etal-2019-ASL12-1H; Makhtoum-etal-2021-LWD-1H; Mohammed-Aziz-HI-1H; Mohammed-Aziz-PH-1H
MQTL1.4	3	Makhtoum-etal-2021-SEVAD-1H; Makhtoum-etal-2021-SND-1H; QTL-Makhtoum-etal-2019-PER-1H
MQTL1.5	4	Jeffrey-2005-COLD-1H; Hanen-Sbei-etal-2014-STI-1H; Hanen-Sbei-etal-2014-LIS-1H; Gashu-Kindu-etal-2014-PhNF-1H
MQTL1.6	18	Haobing-Li-etal-2008-LChl-1H; Mohammed-Aziz-TGW-1H; Mohammed-Aziz-RSR-1H; Mohammed-Aziz-RDW-1H; Xiaohui-LiuX-SA-T-1H; Mohammed-Aziz-WS-1H; Mohammed-2012-WS-1H; Bernhard-Saal-etal-2011-HEI-1H; Dawei-Xue-SPP-1H; Bernhard-Saal-etal-2011-EAR-1H; Arifuzzaman-TKW-1H; Bernhard-Saal-etal-2011-HEA-1H

Continued Table S2.

Meta-QTL	No. of original QTLs	QTLs
MQTL2.1	15	R.P.Ellis-SWt-2H; Xiaohui-LiuX-BM-T.C-2H; Xiaohui-LiuX-TR-T.C-2H; F-TMC-Ap3-2H; Xiaohui-LiuX-GS-T-2H; Yun-Fan-SALV-2H; R.P.Ellis-GS2-2H
MQTL2.2	32	Ghaffari-Moghaddam-etal-1397-FLL-2H; Ghaffari-Moghaddam-etal-2019-SDW15-2H; Yun-2015-DT-2H; Ghaffari-Moghaddam-etal-2019-ASL4-2H; Kornelia-Gudys-PRO-2H; Ayman-OP-2H; Kornelia-Gudys-ETH-2H; Kornelia-Gudys-ABS.RC-2H; Makhtoum-etal-2021-LWS-2H; Yun-2015-RWC-2H; Kornelia-Gudys-WC-2H; QTL-Makhtoum-etal-2019-SUG-2H; Kornelia-Gudys-WL-2H; Bernhard-Saal-etal-2011-HEI-2H; Kornelia-Gudys-TR0.RC-2H; Gaofeng-Zhou-SAL-2H; Mohammed-Aziz-RL-2H; Bernhard-Saal-etal-2011-YLD-2H; Mohammed-2012-WS-2H; Mohammed-Aziz-HI-2H; Mohammed-Aziz-WS-2H
MQTL2.3	9	Arifuzzaman-RL-2H; Makhtoum-etal-2021-AUDPCAD-2H; Bernhard-Saal-etal-2011-TGW-2H; Arifuzzaman-SDW-2H; Arifuzzaman-RDW-2H; Mohammed-Aziz-RDW-2H
MQTL2.4	6	Ghaffari-Moghaddam-etal-2019-SV4-2H; Makhtoum-etal-2021-PLN-2H; Meixue-Zhou-2012-Wl-2H; Bernhard-Saal-etal-2011-HEA-2H; Da-wei-XUE-et_al-_2010-GYW-2H; Mohammed-Aziz-PH-2H
MQTL2.5	23	Mohammed-Aziz-KER-2H; Da-wei-XUE-et_al-_2010-GSW-2H; QTL-Makhtoum-etal-2019-CAT-2H; Mohammed-Aziz-SPS-2H; Arifuzzaman-PH-2H; Mohammed-Aziz-TILS-2H; Makhtoum-etal-2021-FLW-2H; Mohammed-Aziz-RWC-2H; Da-wei-XUE-et_al-_2010-SLW-2H; Haobing-Li-etal-2008-LChl-2H; Dawei-Xue-DWP-2H; Mohammed-Aziz-SDW-2H
MQTL2.6	4	Xiaohui-LiuX-LT-T-2H; Xiaohui-LiuX-LT-C-2H; Dawei-Xue-GNP-2H; Bernhard-Saal-etal-2011-EAR-2H
MQTL2.7	19	Mohammed-Aziz-GY-2H; Ayman-WSC-2H; Xiaohui-LiuX-GY-C-2H; Makhtoum-etal-2021-LWD-2H; Makhtoum-etal-2021-RWD-2H; Haobing-Li-etal-2008-PS-2H; Ayman-DWSC-2H; Ghaffari-Moghaddam-etal-2019-ARL20-2H; Ghaffari-Moghaddam-etal-2019-SFW4-2H; Xiaohui-LiuX-VPD-T.C-2H; Ghaffari-Moghaddam-etal-2019-ARL15-2H
MQTL2.8	6	Rugen-Xu-RDW.P-2H; QTL-Makhtoum-etal-2019-PEO-2H; QTL-Makhtoum-etal-2019-PHE-2H; Kornelia-Gudys-ET0.RC-2H; Rugen-Xu-SAL-2H; Sue-Broughton-etal-LRL-2H
MQTL2.9	6	Rugen-Xu-PHS.C-2H; Rugen-Xu-GSDWS-2H; Rugen-Xu-NoTS-2H; Rugen-Xu-PHS-2H; Rugen-Xu-RFW.P-2H; Rugen-Xu-GSFWS-2H

Continued Table S2.

Meta-QTL	No. of original QTLs	QTLs
MQTL3.1	8	Yun-2015-PCD-3H; Meixue-Zhou-2012-Wl-3H; Ayman-OA-3H; Yun-2015-PCS-3H; Makhtoum-etal-2021-Pm.SEVAS-3H; Makhtoum-etal-2021-SCD-3H
MQTL3.2	24	Bernhard-Saal-etal-2011-YLD-3H; Bernhard-Saal-etal-2011-HEA-3H; Bernhard-Saal-etal-2011-HEI-3H; Gashu-Kindu-etal-2014-TkwNO-3H; Haobing-Li-etal-2008-LYP3H; Makhtoum-etal-2021-LWS-3H; QTL-Makhtoum-etal-2019-PER-3H; Mohammed-Aziz-PH-3H; Ayman-RWC-3H; Makhtoum-etal-2021-AUDPCAD-3H; Haobing-Li-etal-2008-LYP-3H; Sue-Broughton-etal-Tiller-3H; Mohammed-Aziz-SPS-3H; Mohammed-Aziz-KER-3H; Dawei-Xue-SPP-3H
MQTL3.3	13	Xiaohui-LiuX-GY-T-3H; Xiaohui-LiuX-GY-T.C-3H; Haobing-Li-etal-2008-LChl-3H; Xiaohui-LiuX-STC-3H; Ayman-DWSC-3H; Ayman-WSC-3H
MQTL3.4	15	Da-wei-XUE-et_al-_2010-SLW-3H; Arifuzzaman-TKW-3H; Arifuzzaman-PH-3H; Mohammed-2012-WS-3H; Mohammed-Aziz-WS-3H; Arifuzzaman-RDW-3H; Arifuzzaman-RSR-3H; Mohammed-Aziz-HI-3H; Arifuzzaman-RL-3H; Mohammed-Aziz-GY-3H
MQTL3.5	31	Mohammed-Aziz-RL-3H; Mohammed-Aziz-TGW-3H; Mohammed-Aziz-RWC-3H; S.NAVAKODE-etal-ALU-3H; Mohammed-Aziz-RSR-3H; Mohammed-Aziz-RDW-3H; Xiaohui-LiuX-AWL-T-3H; Bernhard-Saal-etal-2011-TGW-3H; Gashu-Kindu-etal-2014-ShwN-3H; Gashu-Kindu-etal-2014-PhNT-3H;Gashu-Kindu-etal-2014-DsNT-3H; Gashu-Kindu-etal-2014-PhNF-3H; Gashu-Kindu-etal-2014-SwN-3H; Gashu-Kindu-etal-2014-ANT-3H; Gashu-Kindu-etal-2014-PhNTR-3H; Gashu-Kindu-etal-2014-PhN0-3H; Gashu-Kindu-etal-2014-DsNF-3H; Gashu-Kindu-etal-2014-NNT-3H; Gashu-Kindu-etal-2014-AgbN-3H; Gashu-Kindu-etal-2014-PhNO-3H; Gashu-Kindu-etal-2014-DsNO-3H; Yun-Fan-SALV-3H; Dawei-Xue-PH-3H
MQTL3.6	8	Ayman-OP-3H; Mohammed-Aziz-PC-3H; Mohammed-2012-PC-3H; Hanen-Sbei-etal-2014-LIS-3H; Makhtoum-etal-2021-LEL-3H

Continued Table S2.

Meta-QTL	No. of original QTLs	QTLs
MQTL4.1	5	Hanen-Sbei-etal-2014-LIS-4H; R.P.Ellis-SWt-4H; R.P.Ellis-GN-4H; Yoshiro-1996-SAL-4H; R.P.Ellis-PY-4H
MQTL4.2	8	Makhtoum-etal-2021-RLD-4H; Makhtoum-etal-2021-RLS-4H; Sue-Broughton-etal-Tiller-4H; Makhtoum-etal-2021-FLW-4H; Makhtoum-etal-2021-LND-4H
MQTL4.3	3	QTL-Makhtoum-etal-2019-SUG-4H; Bernhard-Saal-etal-2011-HEA-4H
MQTL4.4	1	Makhtoum-etal-2021-SWS-4H
MQTL4.5	11	Makhtoum-etal-2021-RWD-4H; Dawei-Xue-SPP-4H; S.NAVAKODE-etal-ALU-4H; Makhtoum-etal-2021-SEVAD-4H; QTL-Makhtoum-etal-2019-PHE-4H; Dawei-Xue-SPL-4H; Makhtoum-etal-2021-SHT-4H
MQTL4.6	18	Mohammed-Aziz-PH-4H; Dawei-Xue-TN-4H; Makhtoum-etal-2021-LEN-4H; Makhtoum-etal-2021-LEL-4H; QTL-Makhtoum-etal-2019-PER-4H; Makhtoum-etal-2021-Pm.AUDPCAS-4H; Makhtoum-etal-2021-LWS-4H; Makhtoum-etal-2021-SLS-4H; Ghaffari-Moghaddam-etal-2019-RFW15-4H; Makhtoum-etal-2021-LNS-4H; Ghaffari-Moghaddam-etal-2019-SDW4-4H; Ghaffari-Moghaddam-etal-2019-SFW15-4H; Makhtoum-etal-2021-SCS-4H
MQTL4.7	14	Meixue-Zhou-2012-Wl-4H; Bernhard-Saal-etal-2011-TGW-4H; Bernhard-Saal-etal-2011-HEI-4H; Haobing-Li-etal-2008-PBR-4H; Bernhard-Saal-etal-2011-EAR-4H; Sue-Broughton-etal-Porosity-4H; Ghaffari-Moghaddam-etal-1397-TLW-4H
MQTL4.8	36	Mohammed-2012-PC-4H; Mohammed-Aziz-PC-4H; Ghaffari-Moghaddam-etal-1397-FSH-4H; Mohammed-Aziz-KER-4H; Arifuzzaman-TKW-4H; Mohammed-Aziz-HI-4H; Arifuzzaman-RDW-4H; Mohammed-Aziz-OP-4H; Jeffrey-2005-COLD-4H; Mohammed-Aziz-WS-4H; Mohammed-2012-WS-4H; Mohammed-Aziz-TGW-4H; Ghaffari-Moghaddam-etal-1397-TNL-4H; Bernhard-Saal-etal-2011-YLD-4H; Mohammed-Aziz-SPS-4H; Yun-Fan-SALV-4H; Haobing-Li-etal-2008-LYP-4H; Arifuzzaman-PH-4H; Mohammed-Aziz-TILS-4H ; Ghaffari-Moghaddam-etal-1397-SD-4H; Haobing-Li-etal-2008-RPB-4H; Ghaffari-Moghaddam-etal-2019-SDW12-1H

Continued Table S2.

Meta-QTL	No. of original QTLs	QTLs
MQTL5.1	2	Yoshiro-1996-SAL-5H; Yoshiro-1996-SAL-7H
MQTL5.2	17	F-FTS-5H; F-FT-5H; F-FT_-5H; F-WS-5H; F-COR14ba-5H; F-TMC-Ap3-5H; F-VrnH1-5H; Yun-Fan-SALV-5H
MQTL5.3	5	R.P.Ellis-SGa-5H; R.P.Ellis-PY-5H; R.P.Ellis-SSC-5H; R.P.Ellis-SWt-5H; R.P.Ellis-RWt-5H
MQTL5.4	9	Makhtoum-etal-2021-RWD-5H; Bernhard-Saal-etal-2011-YLD-5H; Makhtoum-etal-2021-Pm.SEVAS-5H; Makhtoum-etal-2021-PDL-5H; Makhtoum-etal-2021-SHT-5H; Bernhard-Saal-etal-2011-HEI-5H; Bernhard-Saal-etal-2011-EAR-5H; Bernhard-Saal-etal-2011-HEA-5H;Bernhard-Saal-etal-2011-TGW-5H
MQTL5.5	1	Makhtoum-etal-2021-RLD-5H
MQTL5.6	2	Haobing-Li-etal-2008-PS-5H; Haobing-Li-etal-2008-LYP-5H
MQTL5.7	1	Da-wei-XUE-et_al-_2010-GSW-5H
MQTL5.8	12	Mohammed-2012-PC-5H; Mohammed-Aziz-PC-5H; Mohammed-Aziz-RL-5H; Hanen-Sbei-etal-2014-LIS-5H; Mohammed-Aziz-RSR-5H; Arifuzzaman-SDW-5H; Arifuzzaman-RSR-5H; Mohammed-Aziz-SDW-5H; Mohammed-Aziz-RDW-5H; Arifuzzaman-RL-5H; Mohammed-Aziz-HI-5H
MQTL5.9	9	Makhtoum-etal-2021-AUDPCAD-5H; Makhtoum-etal-2021-SEVAD-5H; Makhtoum-etal-2021-LEL-5H; QTL-Makhtoum-etal-2019-SUG-5H; Makhtoum-etal-2021-LFW-5H; Makhtoum-etal-2021-LFW-6H; Makhtoum-etal-2021-FLW-5H; Gashu-Kindu-etal-2014-LwNT-5H; Gashu-Kindu-etal-2014-LwNO-5H

Continued Table S2.

Meta-QTL	No. of original QTLs	QTLs
MQTL6.1	6	Dawei-Xue-Na-K-R-6H; F-COR14ba-6H; F-TMC-Ap3-6H; Gaofeng-Zhou-SAL-6H; Dawei-Xue-GY-6H; Da-wei-XUE-et_al-_2010-PHW-6H
MQTL6.2	2	Litao-Yang-etal-2004-CP-6H; Yoshiro-1996-SAL-6H
MQTL6.3	38	Meixue-Zhou-2012-Wl-6H; Arifuzzaman-TKW-6H; Bernhard-Saal-etal-2011-TGW-6H; Arifuzzaman-SDW-6H; Makhtoum-etal-2021-LWS-6H; Makhtoum-etal-2021-FLW-6H; Makhtoum-etal-2021-LNS-6H; Makhtoum-etal-2021-SEVAD-6H; Bernhard-Saal-etal-2011-EAR-6H; Makhtoum-etal-2021-SHT-6H; Makhtoum-etal-2021-PPT-6H; Makhtoum-etal-2021-SCS-6H; Bernhard-Saal-etal-2011-YLD-6H; Mohammed-Aziz-GY-6H; Bernhard-Saal-etal-2011-HEI-6H; Mohammed-Aziz-TGW-6H; Mohammed-Aziz-PC-6H; Mohammed-2012-PC-6H; Mohammed-Aziz-SDW-6H; Yun-Fan-SALV-6H; Mohammed-Aziz-TILS-6H; Mohammed-Aziz-KER-6H; Mohammed-Aziz-HI-6H; Mohammed-Aziz-SPS-6H
MQTL6.4	13	Ghaffari-Moghaddam-etal-1397-FLDW-6H; X.Huang-etal-2018-LC.yf-6H; Makhtoum-etal-2021-LEN-6H; Makhtoum-etal-2021-LFW-6H; Makhtoum-etal-2021-psi(Eo)-6H; Makhtoum-etal-2021-LEW-6H; Makhtoum-etal-2021-AWW-6H; X.Huang-etal-2018-Sur.yf-6H; Makhtoum-etal-2021-LEL-6H; Makhtoum-etal-2021-SPW-6H; Makhtoum-etal-2021-AUDPCAD-6H

Continued Table S2.

Meta-QTL	No. of original QTLs	QTLs
MQTL7.1	25	Wenta-Xue-2014-RL-7H; Bernhard-Saal-etal-2011-HEA-7H; Bernhard-Saal-etal-2011-HEI-7H; Arifuzzaman-TKW-7H; Yun-2015-ST-7H; Da-wei-XUE-et_al-_2010-GYW-7H; Yun-Fan-SALV-7H; Mohammed-Aziz-TGW-7H; Dawei-Xue-SPL-7H; Da-wei-XUE-et_al-_2010-GSW-7H; Bernhard-Saal-etal-2011-YLD-7H; Bernhard-Saal-etal-2011-EAR-7H

MQTL7.2	6	Kornelia-Gudys-WL-7H; Arifuzzaman-RDW-7H; Arifuzzaman-RSR-7H; Mohammed-Aziz-RDW-7H; Mohammed-Aziz-RSR-7H
MQTL7.3	3	Haobing-Li-etal-2008-LChl-7H; Yuri-SAL-7H
MQTL7.4	8	Kornelia-Gudys-1-B.av-7H; Mei-Han-etal-2016-DN-7H; Mei-Han-etal-2016-YLD-7H; Haobing-Li-etal-2008-LYP-7H
MQTL7.5	7	Makhtoum-etal-2021-SLS-7H; Makhtoum-etal-2021-LEW-7H; Makhtoum-etal-2021-PDL-7H; Makhtoum-etal-2021-AWW-7H
MQTL7.6	12	Makhtoum-etal-2021-PPT-7H; Makhtoum-etal-2021-FLL-7H; Makhtoum-etal-2021-PLS-7H; Makhtoum-etal-2021-LWS-7H; Makhtoum-etal-2021-SCS-7H; Makhtoum-etal-2021-AUDPCAD-7H; Gaofeng-Zhou-SAL-7H; Makhtoum-etal-2021-SCD-7H; Makhtoum-etal-2021-PT-7H; Makhtoum-etal-2021-INL-7H; Makhtoum-etal-2021-LWD-7H; QTL-Makhtoum-etal-2019-PHE-7H
MQTL7.7	3	Mei-Han-etal-2016-GPC-7H
MQTL7.8	3	Makhtoum-etal-2021-LEL-7H; Makhtoum-etal-2021-SEVAD-7H
MQTL7.9	7	Makhtoum-etal-2021-LNS-7H; Makhtoum-etal-2021-RLN-7H; Makhtoum-etal-2021-SPL-7H; Da-wei-XUE-et_al-_2010-SPW-7H; Ayman-DWSC-7H
MQTL7.10	15	Ghaffari-Moghaddam-etal-2019-ARL15-7H; Ayman-WSC-7H; Bernhard-Saal-etal-2011-TGW-7H; Ayman-RWC-7H; QTL-Makhtoum-etal-2019-PER-7H; QTL-Makhtoum-etal-2019-SUG-7H; Makhtoum-etal-2021-SHT-7H
