# Supplementary material for: Mega Meta-QTLs: A Strategy for the Production of Golden Barley (Hordeum vulgare L.) Tolerant to Abiotic Stresses
Source: Genes (Basel). 2022 Nov 10;13(11):2087. doi: 10.3390/genes13112087 (PMC9690463; doi:10.3390/genes13112087)
Supplement: Supplementary file 1 [file genes-13-02087-s001.zip › Table S3.pdf]

Table S3. MQTLs, chromosome number, flanking and closest markers, number of QTLs, studies and population in MQTLs

MQTL	Chromosome	Flanking marker	MQTL position	MQTL confidence interval(cM)	Closest marker	Number of initial QTLs	Number of studies	Number of populations
MQTL1.1	1H	P15M47-314-P15M53-444	60.51	56.145-64.875	*Cmwg645(59.938)-bPb-0395(60.740)	7	4	4
MQTL1.1	1H	P15M47-314-P15M53-444	60.51	56.145-64.875	*Cmwg645(59.938)-bPb-0395(60.740)			
MQTL1.1	1H	P15M47-314-P15M53-444	60.51	56.145-64.875	*Cmwg645(59.938)-bPb-0395(60.740)			
MQTL1.1	1H	P15M47-314-P15M53-444	60.51	56.145-64.875	*Cmwg645(59.938)-bPb-0395(60.740)			
MQTL1.1	1H	P15M47-314-P15M53-444	60.51	56.145-64.875	*Cmwg645(59.938)-bPb-0395(60.740)			
MQTL1.1	1H	P15M47-314-P15M53-444	60.51	56.145-64.875	*Cmwg645(59.938)-bPb-0395(60.740)			
MQTL1.1	1H	P15M47-314-P15M53-444	60.51	56.145-64.875	*Cmwg645(59.938)-bPb-0395(60.740)			
MQTL1.2	1H	bPb-9423-bPb-9957	95.12	92.345-97.895	E32M61-265(94.97)-SCRI_RS_113745(95.12)	14	4	3
MQTL1.2	1H	bPb-9423-bPb-9957	95.12	92.345-97.895	E32M61-265(94.97)-SCRI_RS_113745(95.12)			
MQTL1.2	1H	bPb-9423-bPb-9957	95.12	92.345-97.895	E32M61-265(94.97)-SCRI_RS_113745(95.12)			
MQTL1.2	1H	bPb-9423-bPb-9957	95.12	92.345-97.895	E32M61-265(94.97)-SCRI_RS_113745(95.12)			
MQTL1.2	1H	bPb-9423-bPb-9957	95.12	92.345-97.895	E32M61-265(94.97)-SCRI_RS_113745(95.12)			
MQTL1.2	1H	bPb-9423-bPb-9957	95.12	92.345-97.895	E32M61-265(94.97)-SCRI_RS_113745(95.12)			
MQTL1.2	1H	bPb-9423-bPb-9957	95.12	92.345-97.895	E32M61-265(94.97)-SCRI_RS_113745(95.12)			
MQTL1.2	1H	bPb-9423-bPb-9957	95.12	92.345-97.895	E32M61-265(94.97)-SCRI_RS_113745(95.12)			

Continued Table S3.

MQTL	Chromosome	Flanking marker	MQTL position	MQTL confidence interval(cM)	Closest marker	Number of initial QTLs	Number of studies	Number of populations
MQTL1.2	1H	bPb-9423-bPb-9957	95.12	92.345-97.895	E32M61-265(94.97)-SCRI_RS_113745(95.12)	14	4	3
MQTL1.2	1H	bPb-9423-bPb-9957	95.12	92.345-97.895	E32M61-265(94.97)-SCRI_RS_113745(95.12)			
MQTL1.2	1H	bPb-9423-bPb-9957	95.12	92.345-97.895	E32M61-265(94.97)-SCRI_RS_113745(95.12)			
MQTL1.2	1H	bPb-9423-bPb-9957	95.12	92.345-97.895	E32M61-265(94.97)-SCRI_RS_113745(95.12)			
MQTL1.2	1H	bPb-9423-bPb-9957	95.12	92.345-97.895	E32M61-265(94.97)-SCRI_RS_113745(95.12)			
MQTL1.2	1H	bPb-9423-bPb-9957	95.12	92.345-97.895	E32M61-265(94.97)-SCRI_RS_113745(95.12)			
MQTL1.3	1H	E38M54-441-bPb-7435	107.11	105.135-109.08	E38M55-493(107.09)-0501A(107.11)	18	9	5
MQTL1.3	1H	E38M54-441-bPb-7435	107.11	105.135-109.08	E38M55-493(107.09)-0501A(107.11)			
MQTL1.3	1H	E38M54-441-bPb-7435	107.11	105.135-109.08	E38M55-493(107.09)-0501A(107.11)			
MQTL1.3	1H	E38M54-441-bPb-7435	107.11	105.135-109.08	E38M55-493(107.09)-0501A(107.11)			
MQTL1.3	1H	E38M54-441-bPb-7435	107.11	105.135-109.08	E38M55-493(107.09)-0501A(107.11)			
MQTL1.3	1H	E38M54-441-bPb-7435	107.11	105.135-109.08	E38M55-493(107.09)-0501A(107.11)			
MQTL1.3	1H	E38M54-441-bPb-7435	107.11	105.135-109.08	E38M55-493(107.09)-0501A(107.11)			
MQTL1.3	1H	E38M54-441-bPb-7435	107.11	105.135-109.08	E38M55-493(107.09)-0501A(107.11)			
MQTL1.3	1H	E38M54-441-bPb-7435	107.11	105.135-109.08	E38M55-493(107.09)-0501A(107.11)			

Continued Table S3.

MQTL	Chromosome	Flanking marker	MQTL position	MQTL confidence interval(cM)	Closest marker	Number of initial QTLs	Number of studies	Number of populations
MQTL1.3	1H	E38M54-441-bPb-7435	107.11	105.135-109.08	E38M55-493(107.09)-0501A(107.11)	18	9	5
MQTL1.3	1H	E38M54-441-bPb-7435	107.11	105.135-109.08	E38M55-493(107.09)-0501A(107.11)			
MQTL1.3	1H	E38M54-441-bPb-7435	107.11	105.135-109.08	E38M55-493(107.09)-0501A(107.11)			
MQTL1.3	1H	E38M54-441-bPb-7435	107.11	105.135-109.08	E38M55-493(107.09)-0501A(107.11)			
MQTL1.3	1H	E38M54-441-bPb-7435	107.11	105.135-109.08	E38M55-493(107.09)-0501A(107.11)			
MQTL1.3	1H	E38M54-441-bPb-7435	107.11	105.135-109.08	E38M55-493(107.09)-0501A(107.11)			
MQTL1.3	1H	E38M54-441-bPb-7435	107.11	105.135-109.08	E38M55-493(107.09)-0501A(107.11)			
MQTL1.3	1H	E38M54-441-bPb-7435	107.11	105.135-109.08	E38M55-493(107.09)-0501A(107.11)			
MQTL1.3	1H	E38M54-441-bPb-7435	107.11	105.135-109.08	E38M55-493(107.09)-0501A(107.11)			
MQTL1.4	1H	E42M54-612-E37M50-60	113.71	110.91-116.51	bPb-8763(113.65)-E40M32-654(113.84)	3	3	1
MQTL1.4	1H	E42M54-612-E37M50-60	113.71	110.91-116.51	bPb-8763(113.65)-E40M32-654(113.84)			
MQTL1.4	1H	E42M54-612-E37M50-60	113.71	110.91-116.51	bPb-8763(113.65)-E40M32-654(113.84)			
MQTL1.5	1H	bPb-8477-E35M47-56	135.46	131.9-139.02	His3B(135.24)-ABC152F(135.81)	4	3	3
MQTL1.5	1H	bPb-8477-E35M47-56	135.46	131.9-139.02	His3B(135.24)-ABC152F(135.81)			
MQTL1.5	1H	bPb-8477-E35M47-56	135.46	131.9-139.02	His3B(135.24)-ABC152F(135.81)			

Continued Table S3.

MQTL	Chromosome	Flanking marker	MQTL position	MQTL confidence interval(cM)	Closest marker	Number of initial QTLs	Number of studies	Number of populations
MQTL1.5	1H	bPb-8477-E35M47-56	135.46	131.9-139.02	His3B(135.24)-ABC152F(135.81)	4	3	3
MQTL1.6	1H	bPb-26569-MWG984B	162.94	161.265-164.61	bp5019(162.71)-bPt-5002(163.17)	18	8	3
MQTL1.6	1H	bPb-26569-MWG984B	162.94	161.265-164.61	bp5019(162.71)-bPt-5002(163.17)			
MQTL1.6	1H	bPb-26569-MWG984B	162.94	161.265164.615	bp5019(162.71)-bPt-5002(163.17)			
MQTL1.6	1H	bPb-26569-MWG984B	162.94	161.265-164.61	bp5019(162.71)-bPt-5002(163.17)			
MQTL1.6	1H	bPb-26569-MWG984B	162.94	161.265-164.61	bp5019(162.71)-bPt-5002(163.17)			
MQTL1.6	1H	bPb-26569-MWG984B	162.94	161.265-164.61	bp5019(162.71)-bPt-5002(163.17)			
MQTL1.6	1H	bPb-26569-MWG984B	162.94	161.265-164.61	bp5019(162.71)-bPt-5002(163.17)			
MQTL1.6	1H	bPb-26569-MWG984B	162.94	161.265-164.61	bp5019(162.71)-bPt-5002(163.17)			
MQTL1.6	1H	bPb-26569-MWG984B	162.94	161.265-164.61	bp5019(162.71)-bPt-5002(163.17)			
MQTL1.6	1H	bPb-26569-MWG984B	162.94	161.265-164.61	bp5019(162.71)-bPt-5002(163.17)			
MQTL1.6	1H	bPb-26569-MWG984B	162.94	161.265-164.61	bp5019(162.71)-bPt-5002(163.17)			
MQTL1.6	1H	bPb-26569-MWG984B	162.94	161.265-164.61	bp5019(162.71)-bPt-5002(163.17)			
MQTL1.6	1H	bPb-26569-MWG984B	162.94	161.265-164.61	bp5019(162.71)-bPt-5002(163.17)			
MQTL1.6	1H	bPb-26569-MWG984B	162.94	161.265-164.61	bp5019(162.71)-bPt-5002(163.17)			

Continued Table S3.

MQTL	Chromosome	Flanking marker	MQTL position	MQTL confidence interval(cM)	Closest marker	Number of initial QTLs	Number of studies	Number of populations
MQTL1.6	1H	bPb-26569-MWG984B	162.94	161.265-164.61	bp5019(162.71)-bPt-5002(163.17)	18	8	3
MQTL1.6	1H	bPb-26569-MWG984B	162.94	161.265-164.61	bp5019(162.71)-bPt-5002(163.17)			
MQTL1.6	1H	bPb-26569-MWG984B	162.94	161.265-164.61	bp5019(162.71)-bPt-5002(163.17)			
MQTL1.6	1H	bPb-26569-MWG984B	162.94	161.265-164.61	bp5019(162.71)-bPt-5002(163.17)			
MQTL2.1	2H	bPb-5991 (16.61)-bPb-3186(18.73)	17.68	16.615-18.745	AWBMS62(17.55)-WG516(17.79)	15	4	4
MQTL2.1	2H	bPb-5991 (16.61)-bPb-3186(18.73)	17.68	16.615-18.745	AWBMS62(17.55)-WG516(17.79)			
MQTL2.1	2H	bPb-5991 (16.61)-bPb-3186(18.73)	17.68	16.615-18.745	AWBMS62(17.55)-WG516(17.79)			
MQTL2.1	2H	bPb-5991 (16.61)-bPb-3186(18.73)	17.68	16.615-18.745	AWBMS62(17.55)-WG516(17.79)			
MQTL2.1	2H	bPb-5991 (16.61)-bPb-3186(18.73)	17.68	16.615-18.745	AWBMS62(17.55)-WG516(17.79)			
MQTL2.1	2H	bPb-5991 (16.61)-bPb-3186(18.73)	17.68	16.615-18.745	AWBMS62(17.55)-WG516(17.79)			
MQTL2.1	2H	bPb-5991 (16.61)-bPb-3186(18.73)	17.68	16.615-18.745	AWBMS62(17.55)-WG516(17.79)			
MQTL2.1	2H	bPb-5991 (16.61)-bPb-3186(18.73)	17.68	16.615-18.745	AWBMS62(17.55)-WG516(17.79)			
MQTL2.1	2H	bPb-5991 (16.61)-bPb-3186(18.73)	17.68	16.615-18.745	AWBMS62(17.55)-WG516(17.79)			
MQTL2.1	2H	bPb-5991 (16.61)-bPb-3186(18.73)	17.68	16.615-18.745	AWBMS62(17.55)-WG516(17.79)			
MQTL2.1	2H	bPb-5991 (16.61)-bPb-3186(18.73)	17.68	16.615-18.745	AWBMS62(17.55)-WG516(17.79)			

Continued Table S3.

MQTL	Chromosome	Flanking marker	MQTL position	MQTL confidence interval(cM)	Closest marker	Number of initial QTLs	Number of studies	Number of populations
MQTL2.1	2H	bPb-5991 (16.61)-bPb-3186(18.73)	17.68	16.615-18.745	AWBMS62(17.55)-WG516(17.79)	15	4	4
MQTL2.1	2H	bPb-5991 (16.61)-bPb-3186(18.73)	17.68	16.615-18.745	AWBMS62(17.55)-WG516(17.79)			
MQTL2.1	2H	bPb-5991 (16.61)-bPb-3186(18.73)	17.68	16.615-18.745	AWBMS62(17.55)-WG516(17.79)			
MQTL2.1	2H	bPb-5991 (16.61)-bPb-3186(18.73)	17.68	16.615-18.745	AWBMS62(17.55)-WG516(17.79)			
MQTL2.2	2H	bPb-2405(26.62)-rz828(30.43)	28.56	26.97-30.15	bPb-6235a(28.42)-bPb-6128(28.76)	32	11	6
MQTL2.2	2H	bPb-2405(26.62)-rz828(30.43)	28.56	26.97-30.15	bPb-6235a(28.42)-bPb-6128(28.76)			
MQTL2.2	2H	bPb-2405(26.62)-rz828(30.43)	28.56	26.97-30.15	bPb-6235a(28.42)-bPb-6128(28.76)			
MQTL2.2	2H	bPb-2405(26.62)-rz828(30.43)	28.56	26.97-30.15	bPb-6235a(28.42)-bPb-6128(28.76)			
MQTL2.2	2H	bPb-2405(26.62)-rz828(30.43)	28.56	26.97-30.15	bPb-6235a(28.42)-bPb-6128(28.76)			
MQTL2.2	2H	bPb-2405(26.62)-rz828(30.43)	28.56	26.97-30.15	bPb-6235a(28.42)-bPb-6128(28.76)			
MQTL2.2	2H	bPb-2405(26.62)-rz828(30.43)	28.56	26.97-30.15	bPb-6235a(28.42)-bPb-6128(28.76)			
MQTL2.2	2H	bPb-2405(26.62)-rz828(30.43)	28.56	26.97-30.15	bPb-6235a(28.42)-bPb-6128(28.76)			
MQTL2.2	2H	bPb-2405(26.62)-rz828(30.43)	28.56	26.97-30.15	bPb-6235a(28.42)-bPb-6128(28.76)			
MQTL2.2	2H	bPb-2405(26.62)-rz828(30.43)	28.56	26.97-30.15	bPb-6235a(28.42)-bPb-6128(28.76)			
MQTL2.2	2H	bPb-2405(26.62)-rz828(30.43)	28.56	26.97-30.15	bPb-6235a(28.42)-bPb-6128(28.76)			

Continued Table S3.

MQTL	Chromosome	Flanking marker	MQTL position	MQTL confidence interval(cM)	Closest marker	Number of initial QTLs	Number of studies	Number of populations
MQTL2.2	2H	bPb-2405(26.62)-rz828(30.43)	28.56	26.97-30.15	bPb-6235a(28.42)-bPb-6128(28.76)	32	11	6
MQTL2.2	2H	bPb-2405(26.62)-rz828(30.43)	28.56	26.97-30.15	bPb-6235a(28.42)-bPb-6128(28.76)			
MQTL2.2	2H	bPb-2405(26.62)-rz828(30.43)	28.56	26.97-30.15	bPb-6235a(28.42)-bPb-6128(28.76)			
MQTL2.2	2H	bPb-2405(26.62)-rz828(30.43)	28.56	26.97-30.15	bPb-6235a(28.42)-bPb-6128(28.76)			
MQTL2.2	2H	bPb-2405(26.62)-rz828(30.43)	28.56	26.97-30.15	bPb-6235a(28.42)-bPb-6128(28.76)			
MQTL2.2	2H	bPb-2405(26.62)-rz828(30.43)	28.56	26.97-30.15	bPb-6235a(28.42)-bPb-6128(28.76)			
MQTL2.2	2H	bPb-2405(26.62)-rz828(30.43)	28.56	26.97-30.15	bPb-6235a(28.42)-bPb-6128(28.76)			
MQTL2.2	2H	bPb-2405(26.62)-rz828(30.43)	28.56	26.97-30.15	bPb-6235a(28.42)-bPb-6128(28.76)			
MQTL2.2	2H	bPb-2405(26.62)-rz828(30.43)	28.56	26.97-30.15	bPb-6235a(28.42)-bPb-6128(28.76)			
MQTL2.2	2H	bPb-2405(26.62)-rz828(30.43)	28.56	26.97-30.15	bPb-6235a(28.42)-bPb-6128(28.76)			
MQTL2.2	2H	bPb-2405(26.62)-rz828(30.43)	28.56	26.97-30.15	bPb-6235a(28.42)-bPb-6128(28.76)			
MQTL2.2	2H	bPb-2405(26.62)-rz828(30.43)	28.56	26.97-30.15	bPb-6235a(28.42)-bPb-6128(28.76)			
MQTL2.2	2H	bPb-2405(26.62)-rz828(30.43)	28.56	26.97-30.15	bPb-6235a(28.42)-bPb-6128(28.76)			
MQTL2.2	2H	bPb-2405(26.62)-rz828(30.43)	28.56	26.97-30.15	bPb-6235a(28.42)-bPb-6128(28.76)			
MQTL2.2	2H	bPb-2405(26.62)-rz828(30.43)	28.56	26.97-30.15	bPb-6235a(28.42)-bPb-6128(28.76)			

Continued Table S3.

MQTL	Chromosome	Flanking marker	MQTL position	MQTL confidence interval(cM)	Closest marker	Number of initial QTLs	Number of studies	Number of populations
MQTL2.2	2H	bPb-2405(26.62)-rz828(30.43)	28.56	26.97-30.15	bPb-6235a(28.42)-bPb-6128(28.76)	32	11	6
MQTL2.2	2H	bPb-2405(26.62)-rz828(30.43)	28.56	26.97-30.15	bPb-6235a(28.42)-bPb-6128(28.76)			
MQTL2.2	2H	bPb-2405(26.62)-rz828(30.43)	28.56	26.97-30.15	bPb-6235a(28.42)-bPb-6128(28.76)			
MQTL2.2	2H	bPb-2405(26.62)-rz828(30.43)	28.56	26.97-30.15	bPb-6235a(28.42)-bPb-6128(28.76)			
MQTL2.2	2H	bPb-2405(26.62)-rz828(30.43)	28.56	26.97-30.15	bPb-6235a(28.42)-bPb-6128(28.76)			
MQTL2.2	2H	bPb-2405(26.62)-rz828(30.43)	28.56	26.97-30.15	bPb-6235a(28.42)-bPb-6128(28.76)			
MQTL2.3	2H	bPb-3519(38.17)-bPt-0193(42.74)	40.64	38.17-43.11	BNL16.06(40.61)-bPb-3574(41.08)	9	4	2
MQTL2.3	2H	bPb-3519(38.17)-bPt-0193(42.74)	40.64	38.17-43.11	BNL16.06(40.61)-bPb-3574(41.08)			
MQTL2.3	2H	bPb-3519(38.17)-bPt-0193(42.74)	40.64	38.17-43.11	BNL16.06(40.61)-bPb-3574(41.08)			
MQTL2.3	2H	bPb-3519(38.17)-bPt-0193(42.74)	40.64	38.17-43.11	BNL16.06(40.61)-bPb-3574(41.08)			
MQTL2.3	2H	bPb-3519(38.17)-bPt-0193(42.74)	40.64	38.17-43.11	BNL16.06(40.61)-bPb-3574(41.08)			
MQTL2.3	2H	bPb-3519(38.17)-bPt-0193(42.74)	40.64	38.17-43.11	BNL16.06(40.61)-bPb-3574(41.08)			
MQTL2.3	2H	bPb-3519(38.17)-bPt-0193(42.74)	40.64	38.17-43.11	BNL16.06(40.61)-bPb-3574(41.08)			
MQTL2.3	2H	bPb-3519(38.17)-bPt-0193(42.74)	40.64	38.17-43.11	BNL16.06(40.61)-bPb-3574(41.08)			
MQTL2.3	2H	bPb-3519(38.17)-bPt-0193(42.74)	40.64	38.17-43.11	BNL16.06(40.61)-bPb-3574(41.08)			

Continued Table S3.

MQTL	Chromosome	Flanking marker	MQTL position	MQTL confidence interval(cM)	Closest marker	Number of initial QTLs	Number of studies	Number of populations
MQTL2.4	2H	bPb-7039(56.71)-bPb-3575(59.69)	58.18	56.715-59.645	P61M48h(58.13)-bPb-3575(59.69)	6	6	5
MQTL2.4	2H	bPb-7039(56.71)-bPb-3575(59.69)	58.18	56.715-59.645	P61M48h(58.13)-bPb-3575(59.69)			
MQTL2.4	2H	bPb-7039(56.71)-bPb-3575(59.69)	58.18	56.715-59.645	P61M48h(58.13)-bPb-3575(59.69)			
MQTL2.4	2H	bPb-7039(56.71)-bPb-3575(59.69)	58.18	56.715-59.645	P61M48h(58.13)-bPb-3575(59.69)			
MQTL2.4	2H	bPb-7039(56.71)-bPb-3575(59.69)	58.18	56.715-59.645	P61M48h(58.13)-bPb-3575(59.69)			
MQTL2.4	2H	bPb-7039(56.71)-bPb-3575(59.69)	58.18	56.715-59.645	P61M48h(58.13)-bPb-3575(59.69)			
MQTL2.5	2H	bPb-3056(69.50)-bPb-8511(72.57)	71.03	69.51-72.55	BF064492(71.03)-AWBMA33(71.19)	23	7	5
MQTL2.5	2H	bPb-3056(69.50)-bPb-8511(72.57)	71.03	69.51-72.55	BF064492(71.03)-AWBMA33(71.19)			
MQTL2.5	2H	bPb-3056(69.50)-bPb-8511(72.57)	71.03	69.51-72.55	BF064492(71.03)-AWBMA33(71.19)			
MQTL2.5	2H	bPb-3056(69.50)-bPb-8511(72.57)	71.03	69.51-72.55	BF064492(71.03)-AWBMA33(71.19)			
MQTL2.5	2H	bPb-3056(69.50)-bPb-8511(72.57)	71.03	69.51-72.55	BF064492(71.03)-AWBMA33(71.19)			
MQTL2.5	2H	bPb-3056(69.50)-bPb-8511(72.57)	71.03	69.51-72.55	BF064492(71.03)-AWBMA33(71.19)			
MQTL2.5	2H	bPb-3056(69.50)-bPb-8511(72.57)	71.03	69.51-72.55	BF064492(71.03)-AWBMA33(71.19)			
MQTL2.5	2H	bPb-3056(69.50)-bPb-8511(72.57)	71.03	69.51-72.55	BF064492(71.03)-AWBMA33(71.19)			
MQTL2.5	2H	bPb-3056(69.50)-bPb-8511(72.57)	71.03	69.51-72.55	BF064492(71.03)-AWBMA33(71.19)			

Continued Table S3.

MQTL	Chromosome	Flanking marker	MQTL position	MQTL confidence interval(cM)	Closest marker	Number of initial QTLs	Number of studies	Number of populations
MQTL2.5	2H	bPb-3056(69.50)-bPb-8511(72.57)	71.03	69.51-72.55	BF064492(71.03)-AWBMA33(71.19)	23	7	5
MQTL2.5	2H	bPb-3056(69.50)-bPb-8511(72.57)	71.03	69.51-72.55	BF064492(71.03)-AWBMA33(71.19)			
MQTL2.5	2H	bPb-3056(69.50)-bPb-8511(72.57)	71.03	69.51-72.55	BF064492(71.03)-AWBMA33(71.19)			
MQTL2.5	2H	bPb-3056(69.50)-bPb-8511(72.57)	71.03	69.51-72.55	BF064492(71.03)-AWBMA33(71.19)			
MQTL2.5	2H	bPb-3056(69.50)-bPb-8511(72.57)	71.03	69.51-72.55	BF064492(71.03)-AWBMA33(71.19)			
MQTL2.5	2H	bPb-3056(69.50)-bPb-8511(72.57)	71.03	69.51-72.55	BF064492(71.03)-AWBMA33(71.19)			
MQTL2.5	2H	bPb-3056(69.50)-bPb-8511(72.57)	71.03	69.51-72.55	BF064492(71.03)-AWBMA33(71.19)			
MQTL2.5	2H	bPb-3056(69.50)-bPb-8511(72.57)	71.03	69.51-72.55	BF064492(71.03)-AWBMA33(71.19)			
MQTL2.5	2H	bPb-3056(69.50)-bPb-8511(72.57)	71.03	69.51-72.55	BF064492(71.03)-AWBMA33(71.19)			
MQTL2.5	2H	bPb-3056(69.50)-bPb-8511(72.57)	71.03	69.51-72.55	BF064492(71.03)-AWBMA33(71.19)			
MQTL2.5	2H	bPb-3056(69.50)-bPb-8511(72.57)	71.03	69.51-72.55	BF064492(71.03)-AWBMA33(71.19)			
MQTL2.5	2H	bPb-3056(69.50)-bPb-8511(72.57)	71.03	69.51-72.55	BF064492(71.03)-AWBMA33(71.19)			
MQTL2.5	2H	bPb-3056(69.50)-bPb-8511(72.57)	71.03	69.51-72.55	BF064492(71.03)-AWBMA33(71.19)			
MQTL2.5	2H	bPb-3056(69.50)-bPb-8511(72.57)	71.03	69.51-72.55	BF064492(71.03)-AWBMA33(71.19)			
MQTL2.6	2H	Bmac0273C(77.87)-Bmag125(80.33)	79.19	78.355-80.025	Bmag0711(49.14)-EBmac521a(80.08)	4	3	2

Continued Table S3.

MQTL	Chromosome	Flanking marker	MQTL position	MQTL confidence interval(cM)	Closest marker	Number of initial QTLs	Number of studies	Number of populations
MQTL2.6	2H	Bmac0273C(77.87)-Bmag125(80.33)	79.19	78.355-80.025	Bmag0711(49.14)-EBmac521a(80.08)	4	3	2
MQTL2.6	2H	Bmac0273C(77.87)-Bmag125(80.33)	79.19	78.355-80.025	Bmag0711(49.14)-EBmac521a(80.08)			
MQTL2.6	2H	Bmac0273C(77.87)-Bmag125(80.33)	79.19	78.355-80.025	Bmag0711(49.14)-EBmac521a(80.08)			
MQTL2.7	2H	bPb-8274(107.28)-BM444414.1(112.80)	109.91	107.015-112.805	EBmatc0039(109.90)-AWBMA21(109.95)	19	6	6
MQTL2.7	2H	bPb-8274(107.28)-BM444414.1(112.80)	109.91	107.015-112.805	EBmatc0039(109.90)-AWBMA21(109.95)			
MQTL2.7	2H	bPb-8274(107.28)-BM444414.1(112.80)	109.91	107.015-112.805	EBmatc0039(109.90)-AWBMA21(109.95)			
MQTL2.7	2H	bPb-8274(107.28)-BM444414.1(112.80)	109.91	107.015-112.805	EBmatc0039(109.90)-AWBMA21(109.95)			
MQTL2.7	2H	bPb-8274(107.28)-BM444414.1(112.80)	109.91	107.015-112.805	EBmatc0039(109.90)-AWBMA21(109.95)			
MQTL2.7	2H	bPb-8274(107.28)-BM444414.1(112.80)	109.91	107.015-112.805	EBmatc0039(109.90)-AWBMA21(109.95)			
MQTL2.7	2H	bPb-8274(107.28)-BM444414.1(112.80)	109.91	107.015-112.805	EBmatc0039(109.90)-AWBMA21(109.95)			
MQTL2.7	2H	bPb-8274(107.28)-BM444414.1(112.80)	109.91	107.015-112.805	EBmatc0039(109.90)-AWBMA21(109.95)			
MQTL2.7	2H	bPb-8274(107.28)-BM444414.1(112.80)	109.91	107.015-112.805	EBmatc0039(109.90)-AWBMA21(109.95)			
MQTL2.7	2H	bPb-8274(107.28)-BM444414.1(112.80)	109.91	107.015-112.805	EBmatc0039(109.90)-AWBMA21(109.95)			
MQTL2.7	2H	bPb-8274(107.28)-BM444414.1(112.80)	109.91	107.015-112.805	EBmatc0039(109.90)-AWBMA21(109.95)			
MQTL2.7	2H	bPb-8274(107.28)-BM444414.1(112.80)	109.91	107.015-112.805	EBmatc0039(109.90)-AWBMA21(109.95)			

Continued Table S3.

MQTL	Chromosome	Flanking marker	MQTL position	MQTL confidence interval(cM)	Closest marker	Number of initial QTLs	Number of studies	Number of populations
MQTL2.7	2H	bPb-8274(107.28)-BM444414.1(112.80)	109.91	107.015-112.805	EBmatc0039(109.90)-AWBMA21(109.95)	19	6	6
MQTL2.7	2H	bPb-8274(107.28)-BM444414.1(112.80)	109.91	107.015-112.805	EBmatc0039(109.90)-AWBMA21(109.95)			
MQTL2.7	2H	bPb-8274(107.28)-BM444414.1(112.80)	109.91	107.015-112.805	EBmatc0039(109.90)-AWBMA21(109.95)			
MQTL2.7	2H	bPb-8274(107.28)-BM444414.1(112.80)	109.91	107.015-112.805	EBmatc0039(109.90)-AWBMA21(109.95)			
MQTL2.7	2H	bPb-8274(107.28)-BM444414.1(112.80)	109.91	107.015-112.805	EBmatc0039(109.90)-AWBMA21(109.95)			
MQTL2.7	2H	bPb-8274(107.28)-BM444414.1(112.80)	109.91	107.015-112.805	EBmatc0039(109.90)-AWBMA21(109.95)			
MQTL2.7	2H	bPb-8274(107.28)-BM444414.1(112.80)	109.91	107.015-112.805	EBmatc0039(109.90)-AWBMA21(109.95)			
MQTL2.8	2H	bPb-3858(121.32)-bpb-6297(124.49)	122.88	121.31-124.45	BQ740141(122.82)-3179-497(122.96)	6	4	4
MQTL2.8	2H	bPb-3858(121.32)-bpb-6297(124.49)	122.88	121.31-124.45	BQ740141(122.82)-3179-497(122.96)			
MQTL2.8	2H	bPb-3858(121.32)-bpb-6297(124.49)	122.88	121.31-124.45	BQ740141(122.82)-3179-497(122.96)			
MQTL2.8	2H	bPb-3858(121.32)-bpb-6297(124.49)	122.88	121.31-124.45	BQ740141(122.82)-3179-497(122.96)			
MQTL2.8	2H	bPb-3858(121.32)-bpb-6297(124.49)	122.88	121.31-124.45	BQ740141(122.82)-3179-497(122.96)			
MQTL2.8	2H	bPb-3858(121.32)-bpb-6297(124.49)	122.88	121.31-124.45	BQ740141(122.82)-3179-497(122.96)			
MQTL2.9	2H	BM815937(221.69)-BM816122(257.49)	255.19	254.055-256.325	BM815937(221.69)-BM816122(257.49)	6	1	1
MQTL2.9	2H	BM815937(221.69)-BM816122(257.49)	255.19	254.055-256.325	BM815937(221.69)-BM816122(257.49)			

Continued Table S3.

MQTL	Chromosome	Flanking marker	MQTL position	MQTL confidence interval(cM)	Closest marker	Number of initial QTLs	Number of studies	Number of populations
MQTL2.9	2H	BM815937(221.69)-BM816122(257.49)	255.19	254.055-256.325	BM815937(221.69)-BM816122(257.49)	6	1	1
MQTL2.9	2H	BM815937(221.69)-BM816122(257.49)	255.19	254.055-256.325	BM815937(221.69)-BM816122(257.49)			
MQTL2.9	2H	BM815937(221.69)-BM816122(257.49)	255.19	254.055-256.325	BM815937(221.69)-BM816122(257.49)			
MQTL2.9	2H	BM815937(221.69)-BM816122(257.49)	255.19	254.055-256.325	BM815937(221.69)-BM816122(257.49)			
MQTL3.1	3H	SCRI_RS_7396(192.19)-MWG571C(104.33)	101.74	99.2-104.28	E32M62-92(101.28)-E35M48-250(101.83)	8	6	4
MQTL3.1	3H	SCRI_RS_7396(192.19)-MWG571C(104.33)	101.74	99.2-104.28	E32M62-92(101.28)-E35M48-250(101.83)			
MQTL3.1	3H	SCRI_RS_7396(192.19)-MWG571C(104.33)	101.74	99.2-104.28	E32M62-92(101.28)-E35M48-250(101.83)			
MQTL3.1	3H	SCRI_RS_7396(192.19)-MWG571C(104.33)	101.74	99.2-104.28	E32M62-92(101.28)-E35M48-250(101.83)			
MQTL3.1	3H	SCRI_RS_7396(192.19)-MWG571C(104.33)	101.74	99.2-104.28	E32M62-92(101.28)-E35M48-250(101.83)			
MQTL3.1	3H	SCRI_RS_7396(192.19)-MWG571C(104.33)	101.74	99.2-104.28	E32M62-92(101.28)-E35M48-250(101.83)			
MQTL3.1	3H	SCRI_RS_7396(192.19)-MWG571C(104.33)	101.74	99.2-104.28	E32M62-92(101.28)-E35M48-250(101.83)			
MQTL3.1	3H	SCRI_RS_7396(192.19)-MWG571C(104.33)	101.74	99.2-104.28	E32M62-92(101.28)-E35M48-250(101.83)			
MQTL3.2	3H	GBMS089(142.78)-bPb-4660(145.65)	143.96	142.755-145.65	E41M61-400(143.95)-E42M51-442(143.96)	24	9	6
MQTL3.2	3H	GBMS089(142.78)-bPb-4660(145.65)	143.96	142.755-145.65	E41M61-400(143.95)-E42M51-442(143.96)			
MQTL3.2	3H	GBMS089(142.78)-bPb-4660(145.65)	143.96	142.755-145.65	E41M61-400(143.95)-E42M51-442(143.96)			

Continued Table S3.

MQTL	Chromosome	Flanking marker	MQTL position	MQTL confidence interval(cM)	Closest marker	Number of initial QTLs	Number of studies	Number of populations
MQTL3.2	3H	GBMS089(142.78)-bPb-4660(145.65)	143.96	142.755-145.65	E41M61-400(143.95)-E42M51-442(143.96)	24	9	6
MQTL3.2	3H	GBMS089(142.78)-bPb-4660(145.65)	143.96	142.755-145.65	E41M61-400(143.95)-E42M51-442(143.96)			
MQTL3.2	3H	GBMS089(142.78)-bPb-4660(145.65)	143.96	142.755-145.65	E41M61-400(143.95)-E42M51-442(143.96)			
MQTL3.2	3H	GBMS089(142.78)-bPb-4660(145.65)	143.96	142.755-145.65	E41M61-400(143.95)-E42M51-442(143.96)			
MQTL3.2	3H	GBMS089(142.78)-bPb-4660(145.65)	143.96	142.755-145.65	E41M61-400(143.95)-E42M51-442(143.96)			
MQTL3.2	3H	GBMS089(142.78)-bPb-4660(145.65)	143.96	142.755-145.65	E41M61-400(143.95)-E42M51-442(143.96)			
MQTL3.2	3H	GBMS089(142.78)-bPb-4660(145.65)	143.96	142.755-145.65	E41M61-400(143.95)-E42M51-442(143.96)			
MQTL3.2	3H	GBMS089(142.78)-bPb-4660(145.65)	143.96	142.755-145.65	E41M61-400(143.95)-E42M51-442(143.96)			
MQTL3.2	3H	GBMS089(142.78)-bPb-4660(145.65)	143.96	142.755-145.65	E41M61-400(143.95)-E42M51-442(143.96)			
MQTL3.2	3H	GBMS089(142.78)-bPb-4660(145.65)	143.96	142.755-145.65	E41M61-400(143.95)-E42M51-442(143.96)			
MQTL3.2	3H	GBMS089(142.78)-bPb-4660(145.65)	143.96	142.755-145.65	E41M61-400(143.95)-E42M51-442(143.96)			
MQTL3.2	3H	GBMS089(142.78)-bPb-4660(145.65)	143.96	142.755-145.65	E41M61-400(143.95)-E42M51-442(143.96)			
MQTL3.2	3H	GBMS089(142.78)-bPb-4660(145.65)	143.96	142.755-145.65	E41M61-400(143.95)-E42M51-442(143.96)			
MQTL3.2	3H	GBMS089(142.78)-bPb-4660(145.65)	143.96	142.755-145.65	E41M61-400(143.95)-E42M51-442(143.96)			
MQTL3.2	3H	GBMS089(142.78)-bPb-4660(145.65)	143.96	142.755-145.65	E41M61-400(143.95)-E42M51-442(143.96)			

Continued Table S3.

MQTL	Chromosome	Flanking marker	MQTL position	MQTL confidence interval(cM)	Closest marker	Number of initial QTLs	Number of studies	Number of populations
MQTL3.2	3H	GBMS089(142.78)-bPb-4660(145.65)	143.96	142.755-145.65	E41M61-400(143.95)-E42M51-442(143.96)	24	9	6
MQTL3.2	3H	GBMS089(142.78)-bPb-4660(145.65)	143.96	142.755-145.65	E41M61-400(143.95)-E42M51-442(143.96)			
MQTL3.2	3H	GBMS089(142.78)-bPb-4660(145.65)	143.96	142.755-145.65	E41M61-400(143.95)-E42M51-442(143.96)			
MQTL3.2	3H	GBMS089(142.78)-bPb-4660(145.65)	143.96	142.755-145.65	E41M61-400(143.95)-E42M51-442(143.96)			
MQTL3.2	3H	GBMS089(142.78)-bPb-4660(145.65)	143.96	142.755-145.65	E41M61-400(143.95)-E42M51-442(143.96)			
MQTL3.2	3H	GBMS089(142.78)-bPb-4660(145.65)	143.96	142.755-145.65	E41M61-400(143.95)-E42M51-442(143.96)			
MQTL3.3	3H	SCRI_RS_201075(162.23)-SCRI_RS_230023(168.75)	165.44	162.305-168.575	bPb-3320(165.22)-basd27g02(165.47)	13	3	3
MQTL3.3	3H	SCRI_RS_201075(162.23)-SCRI_RS_230023(168.75)	165.44	162.305-168.575	bPb-3320(165.22)-basd27g02(165.47)			
MQTL3.3	3H	SCRI_RS_201075(162.23)-SCRI_RS_230023(168.75)	165.44	162.305-168.575	bPb-3320(165.22)-basd27g02(165.47)			
MQTL3.3	3H	SCRI_RS_201075(162.23)-SCRI_RS_230023(168.75)	165.44	162.305-168.575	bPb-3320(165.22)-basd27g02(165.47)			
MQTL3.3	3H	SCRI_RS_201075(162.23)-SCRI_RS_230023(168.75)	165.44	162.305-168.575	bPb-3320(165.22)-basd27g02(165.47)			
MQTL3.3	3H	SCRI_RS_201075(162.23)-SCRI_RS_230023(168.75)	165.44	162.305-168.575	bPb-3320(165.22)-basd27g02(165.47)			
MQTL3.3	3H	SCRI_RS_201075(162.23)-SCRI_RS_230023(168.75)	165.44	162.305-168.575	bPb-3320(165.22)-basd27g02(165.47)			
MQTL3.3	3H	SCRI_RS_201075(162.23)-SCRI_RS_230023(168.75)	165.44	162.305-168.575	bPb-3320(165.22)-basd27g02(165.47)			
MQTL3.3	3H	SCRI_RS_201075(162.23)-SCRI_RS_230023(168.75)	165.44	162.305-168.575	bPb-3320(165.22)-basd27g02(165.47)			

Continued Table S3.

MQTL	Chromosome	Flanking marker	MQTL position	MQTL confidence interval(cM)	Closest marker	Number of initial QTLs	Number of studies	Number of populations
MQTL3.3	3H	SCRI_RS_201075(162.23)-SCRI_RS_230023(168.75)	165.44	162.305-168.575	bPb-3320(165.22)-basd27g02(165.47)	13	3	3
MQTL3.3	3H	SCRI_RS_201075(162.23)-SCRI_RS_230023(168.75)	165.44	162.305-168.575	bPb-3320(165.22)-basd27g02(165.47)			
MQTL3.3	3H	SCRI_RS_201075(162.23)-SCRI_RS_230023(168.75)	165.44	162.305-168.575	bPb-3320(165.22)-basd27g02(165.47)			
MQTL3.3	3H	SCRI_RS_201075(162.23)-SCRI_RS_230023(168.75)	165.44	162.305-168.575	bPb-3320(165.22)-basd27g02(165.47)			
MQTL3.4	3H	GBM1014(168.64)-WG940(180.24)	179.49	178.6-180.38	bPt-6567(179.18)-bPt-5150(179.52)	15	4	2
MQTL3.4	3H	GBM1014(168.64)-WG940(180.24)	179.49	178.6-180.38	bPt-6567(179.18)-bPt-5150(179.52)			
MQTL3.4	3H	GBM1014(168.64)-WG940(180.24)	179.49	178.6-180.38	bPt-6567(179.18)-bPt-5150(179.52)			
MQTL3.4	3H	GBM1014(168.64)-WG940(180.24)	179.49	178.6-180.38	bPt-6567(179.18)-bPt-5150(179.52)			
MQTL3.4	3H	GBM1014(168.64)-WG940(180.24)	179.49	178.6-180.38	bPt-6567(179.18)-bPt-5150(179.52)			
MQTL3.4	3H	GBM1014(168.64)-WG940(180.24)	179.49	178.6-180.38	bPt-6567(179.18)-bPt-5150(179.52)			
MQTL3.4	3H	GBM1014(168.64)-WG940(180.24)	179.49	178.6-180.38	bPt-6567(179.18)-bPt-5150(179.52)			
MQTL3.4	3H	GBM1014(168.64)-WG940(180.24)	179.49	178.6-180.38	bPt-6567(179.18)-bPt-5150(179.52)			
MQTL3.4	3H	GBM1014(168.64)-WG940(180.24)	179.49	178.6-180.38	bPt-6567(179.18)-bPt-5150(179.52)			
MQTL3.4	3H	GBM1014(168.64)-WG940(180.24)	179.49	178.6-180.38	bPt-6567(179.18)-bPt-5150(179.52)			
MQTL3.4	3H	GBM1014(168.64)-WG940(180.24)	179.49	178.6-180.38	bPt-6567(179.18)-bPt-5150(179.52)			

Continued Table S3.

MQTL	Chromosome	Flanking marker	MQTL position	MQTL confidence interval(cM)	Closest marker	Number of initial QTLs	Number of studies	Number of populations
MQTL3.4	3H	GBM1014(168.64)-WG940(180.24)	179.49	178.6-180.38	bPt-6567(179.18)-bPt-5150(179.52)	15	4	2
MQTL3.4	3H	GBM1014(168.64)-WG940(180.24)	179.49	178.6-180.38	bPt-6567(179.18)-bPt-5150(179.52)			
MQTL3.4	3H	GBM1014(168.64)-WG940(180.24)	179.49	178.6-180.38	bPt-6567(179.18)-bPt-5150(179.52)			
MQTL3.4	3H	GBM1014(168.64)-WG940(180.24)	179.49	178.6-180.38	bPt-6567(179.18)-bPt-5150(179.52)			
MQTL3.5	3H	2500-1514(187.99)-bp5488(190.33)	189.08	187.81-190.35	bp4025(189.03)-7241-553(189.17)	31	7	5
MQTL3.5	3H	2500-1514(187.99)-bp5488(190.33)	189.08	187.81-190.35	bp4025(189.03)-7241-553(189.17)			
MQTL3.5	3H	2500-1514(187.99)-bp5488(190.33)	189.08	187.81-190.35	bp4025(189.03)-7241-553(189.17)			
MQTL3.5	3H	2500-1514(187.99)-bp5488(190.33)	189.08	187.81-190.35	bp4025(189.03)-7241-553(189.17)			
MQTL3.5	3H	2500-1514(187.99)-bp5488(190.33)	189.08	187.81-190.35	bp4025(189.03)-7241-553(189.17)			
MQTL3.5	3H	2500-1514(187.99)-bp5488(190.33)	189.08	187.81-190.35	bp4025(189.03)-7241-553(189.17)			
MQTL3.5	3H	2500-1514(187.99)-bp5488(190.33)	189.08	187.81-190.35	bp4025(189.03)-7241-553(189.17)			
MQTL3.5	3H	2500-1514(187.99)-bp5488(190.33)	189.08	187.81-190.35	bp4025(189.03)-7241-553(189.17)			
MQTL3.5	3H	2500-1514(187.99)-bp5488(190.33)	189.08	187.81-190.35	bp4025(189.03)-7241-553(189.17)			
MQTL3.5	3H	2500-1514(187.99)-bp5488(190.33)	189.08	187.81-190.35	bp4025(189.03)-7241-553(189.17)			
MQTL3.5	3H	2500-1514(187.99)-bp5488(190.33)	189.08	187.81-190.35	bp4025(189.03)-7241-553(189.17)			

Continued Table S3.

MQTL	Chromosome	Flanking marker	MQTL position	MQTL confidence interval(cM)	Closest marker	Number of initial QTLs	Number of studies	Number of populations
MQTL3.5	3H	2500-1514(187.99)-bp5488(190.33)	189.08	187.81-190.35	bp4025(189.03)-7241-553(189.17)	31	7	5
MQTL3.5	3H	2500-1514(187.99)-bp5488(190.33)	189.08	187.81-190.35	bp4025(189.03)-7241-553(189.17)			
MQTL3.5	3H	2500-1514(187.99)-bp5488(190.33)	189.08	187.81-190.35	bp4025(189.03)-7241-553(189.17)			
MQTL3.5	3H	2500-1514(187.99)-bp5488(190.33)	189.08	187.81-190.35	bp4025(189.03)-7241-553(189.17)			
MQTL3.5	3H	2500-1514(187.99)-bp5488(190.33)	189.08	187.81-190.35	bp4025(189.03)-7241-553(189.17)			
MQTL3.5	3H	2500-1514(187.99)-bp5488(190.33)	189.08	187.81-190.35	bp4025(189.03)-7241-553(189.17)			
MQTL3.5	3H	2500-1514(187.99)-bp5488(190.33)	189.08	187.81-190.35	bp4025(189.03)-7241-553(189.17)			
MQTL3.5	3H	2500-1514(187.99)-bp5488(190.33)	189.08	187.81-190.35	bp4025(189.03)-7241-553(189.17)			
MQTL3.5	3H	2500-1514(187.99)-bp5488(190.33)	189.08	187.81-190.35	bp4025(189.03)-7241-553(189.17)			
MQTL3.5	3H	2500-1514(187.99)-bp5488(190.33)	189.08	187.81-190.35	bp4025(189.03)-7241-553(189.17)			
MQTL3.5	3H	2500-1514(187.99)-bp5488(190.33)	189.08	187.81-190.35	bp4025(189.03)-7241-553(189.17)			
MQTL3.5	3H	2500-1514(187.99)-bp5488(190.33)	189.08	187.81-190.35	bp4025(189.03)-7241-553(189.17)			
MQTL3.5	3H	2500-1514(187.99)-bp5488(190.33)	189.08	187.81-190.35	bp4025(189.03)-7241-553(189.17)			
MQTL3.5	3H	2500-1514(187.99)-bp5488(190.33)	189.08	187.81-190.35	bp4025(189.03)-7241-553(189.17)			
MQTL3.5	3H	2500-1514(187.99)-bp5488(190.33)	189.08	187.81-190.35	bp4025(189.03)-7241-553(189.17)			

Continued Table S3.

MQTL	Chromosome	Flanking marker	MQTL position	MQTL confidence interval(cM)	Closest marker	Number of initial QTLs	Number of studies	Number of populations
MQTL3.5	3H	2500-1514(187.99)-bp5488(190.33)	189.08	187.81-190.35	bp4025(189.03)-7241-553(189.17)	31	7	5
MQTL3.5	3H	2500-1514(187.99)-bp5488(190.33)	189.08	187.81-190.35	bp4025(189.03)-7241-553(189.17)			
MQTL3.5	3H	2500-1514(187.99)-bp5488(190.33)	189.08	187.81-190.35	bp4025(189.03)-7241-553(189.17)			
MQTL3.5	3H	2500-1514(187.99)-bp5488(190.33)	189.08	187.81-190.35	bp4025(189.03)-7241-553(189.17)			
MQTL3.5	3H	2500-1514(187.99)-bp5488(190.33)	189.08	187.81-190.35	bp4025(189.03)-7241-553(189.17)			
MQTL3.6	3H	MWG932(213.54)-bPt-7973(221.50)	217.54	213.575-221.505	ABA302(217.25)-P15M47-91(217.59)	8	5	4
MQTL3.6	3H	MWG932(213.54)-bPt-7973(221.50)	217.54	213.575-221.505	ABA302(217.25)-P15M47-91(217.59)			
MQTL3.6	3H	MWG932(213.54)-bPt-7973(221.50)	217.54	213.575-221.505	ABA302(217.25)-P15M47-91(217.59)			
MQTL3.6	3H	MWG932(213.54)-bPt-7973(221.50)	217.54	213.575-221.505	ABA302(217.25)-P15M47-91(217.59)			
MQTL3.6	3H	MWG932(213.54)-bPt-7973(221.50)	217.54	213.575-221.505	ABA302(217.25)-P15M47-91(217.59)			
MQTL3.6	3H	MWG932(213.54)-bPt-7973(221.50)	217.54	213.575-221.505	ABA302(217.25)-P15M47-91(217.59)			
MQTL3.6	3H	MWG932(213.54)-bPt-7973(221.50)	217.54	213.575-221.505	ABA302(217.25)-P15M47-91(217.59)			
MQTL3.6	3H	MWG932(213.54)-bPt-7973(221.50)	217.54	213.575-221.505	ABA302(217.25)-P15M47-91(217.59)			
MQTL4.1	4H	BOPA1_2065-3135(78.99)-SCRI_RS_12719(87.60)	82.66	79.615-87.705	SCRI_RS_180891(80.50)-SCRI_RS_119628(83.45)	5	3	3
MQTL4.1	4H	BOPA1_2065-3135(78.99)-SCRI_RS_12719(87.60)	82.66	79.615-87.705	SCRI_RS_180891(80.50)-SCRI_RS_119628(83.45)			

Continued Table S3.

MQTL	Chromosome	Flanking marker	MQTL position	MQTL confidence interval(cM)	Closest marker	Number of initial QTLs	Number of studies	Number of populations
MQTL4.1	4H	BOPA1_2065-3135(78.99)-SCRI_RS_12719(87.60)	82.66	79.615-87.705	SCRI_RS_180891(80.50)-SCRI_RS_119628(83.45)	5	3	3
MQTL4.1	4H	BOPA1_2065-3135(78.99)-SCRI_RS_12719(87.60)	82.66	79.615-87.705	SCRI_RS_180891(80.50)-SCRI_RS_119628(83.45)			
MQTL4.1	4H	BOPA1_2065-3135(78.99)-SCRI_RS_12719(87.60)	82.66	79.615-87.705	SCRI_RS_180891(80.50)-SCRI_RS_119628(83.45)			
MQTL4.2	4H	MWG77(109.99)-SCRI_RS_168610(115.78)	113.31	110.89-115.73	BOPA2_12_10063(113.09)-E36M62-78(113.51)	8	4	2
MQTL4.2	4H	MWG77(109.99)-SCRI_RS_168610(115.78)	113.31	110.89-115.73	BOPA2_12_10063(113.09)-E36M62-78(113.51)			
MQTL4.2	4H	MWG77(109.99)-SCRI_RS_168610(115.78)	113.31	110.89-115.73	BOPA2_12_10063(113.09)-E36M62-78(113.51)			
MQTL4.2	4H	MWG77(109.99)-SCRI_RS_168610(115.78)	113.31	110.89-115.73	BOPA2_12_10063(113.09)-E36M62-78(113.51)			
MQTL4.2	4H	MWG77(109.99)-SCRI_RS_168610(115.78)	113.31	110.89-115.73	BOPA2_12_10063(113.09)-E36M62-78(113.51)			
MQTL4.2	4H	MWG77(109.99)-SCRI_RS_168610(115.78)	113.31	110.89-115.73	BOPA2_12_10063(113.09)-E36M62-78(113.51)			
MQTL4.2	4H	MWG77(109.99)-SCRI_RS_168610(115.78)	113.31	110.89-115.73	BOPA2_12_10063(113.09)-E36M62-78(113.51)			
MQTL4.2	4H	MWG77(109.99)-SCRI_RS_168610(115.78)	113.31	110.89-115.73	BOPA2_12_10063(113.09)-E36M62-78(113.51)			
MQTL4.3	4H	E38M55-430(125.72)-GBMS87(132.88)	129.23	125.745-132.715	basd13l12(128.85)-E33M60--5.5(129.43)	3	2	2
MQTL4.3	4H	E38M55-430(125.72)-GBMS87(132.88)	129.23	125.745-132.715	basd13l12(128.85)-E33M60--5.5(129.43)			
MQTL4.3	4H	E38M55-430(125.72)-GBMS87(132.88)	129.23	125.745-132.715	basd13l12(128.85)-E33M60--5.5(129.43)			
MQTL4.4	4H	baal9m23(137.57)-E32M60-172(149.81)	143.73	137.585-149.875	E40M32-153(143.70)-E36M59-94(143.91)	1	1	1

Continued Table S3.

MQTL	Chromosome	Flanking marker	MQTL position	MQTL confidence interval(cM)	Closest marker	Number of initial QTLs	Number of studies	Number of populations
MQTL4.5	4H	bPb-1278(150.51)-Bmac0175a(156.57)	153.54	150.515-156.565	E33M54-416(153.49)-bPb-6872(153.58)	11	6	3
MQTL4.5	4H	bPb-1278(150.51)-Bmac0175a(156.57)	153.54	150.515-156.565	E33M54-416(153.49)-bPb-6872(153.58)			
MQTL4.5	4H	bPb-1278(150.51)-Bmac0175a(156.57)	153.54	150.515-156.565	E33M54-416(153.49)-bPb-6872(153.58)			
MQTL4.5	4H	bPb-1278(150.51)-Bmac0175a(156.57)	153.54	150.515-156.565	E33M54-416(153.49)-bPb-6872(153.58)			
MQTL4.5	4H	bPb-1278(150.51)-Bmac0175a(156.57)	153.54	150.515-156.565	E33M54-416(153.49)-bPb-6872(153.58)			
MQTL4.5	4H	bPb-1278(150.51)-Bmac0175a(156.57)	153.54	150.515-156.565	E33M54-416(153.49)-bPb-6872(153.58)			
MQTL4.5	4H	bPb-1278(150.51)-Bmac0175a(156.57)	153.54	150.515-156.565	E33M54-416(153.49)-bPb-6872(153.58)			
MQTL4.5	4H	bPb-1278(150.51)-Bmac0175a(156.57)	153.54	150.515-156.565	E33M54-416(153.49)-bPb-6872(153.58)			
MQTL4.5	4H	bPb-1278(150.51)-Bmac0175a(156.57)	153.54	150.515-156.565	E33M54-416(153.49)-bPb-6872(153.58)			
MQTL4.5	4H	bPb-1278(150.51)-Bmac0175a(156.57)	153.54	150.515-156.565	E33M54-416(153.49)-bPb-6872(153.58)			
MQTL4.5	4H	bPb-1278(150.51)-Bmac0175a(156.57)	153.54	150.515-156.565	E33M54-416(153.49)-bPb-6872(153.58)			
MQTL4.6	4H	bPt-6055(157.58)-bPt-5419(161.91)	159.69	157.505-161.875	E32M62-386(158.61)-E38M55-139(158.80)	18	7	4
MQTL4.6	4H	bPt-6055(157.58)-bPt-5419(161.91)	159.69	157.505-161.875	E32M62-386(158.61)-E38M55-139(158.80)			
MQTL4.6	4H	bPt-6055(157.58)-bPt-5419(161.91)	159.69	157.505-161.875	E32M62-386(158.61)-E38M55-139(158.80)			
MQTL4.6	4H	bPt-6055(157.58)-bPt-5419(161.91)	159.69	157.505-161.875	E32M62-386(158.61)-E38M55-139(158.80)			

Continued Table S3.

MQTL	Chromo some	Flanking marker	MQTL position	MQTL confidence interval(cM)	Closest marker	Number of initial QTLs	Number of studies	Number of populatio ns
MQTL4.6	4H	bPt-6055(157.58)-bPt-5419(161.91)	159.69	157.505-161.875	E32M62-386(158.61)-E38M55-139(158.80)	18	7	4
MQTL4.6	4H	bPt-6055(157.58)-bPt-5419(161.91)	159.69	157.505-161.875	E32M62-386(158.61)-E38M55-139(158.80)			
MQTL4.6	4H	bPt-6055(157.58)-bPt-5419(161.91)	159.69	157.505-161.875	E32M62-386(158.61)-E38M55-139(158.80)			
MQTL4.6	4H	bPt-6055(157.58)-bPt-5419(161.91)	159.69	157.505-161.875	E32M62-386(158.61)-E38M55-139(158.80)			
MQTL4.6	4H	bPt-6055(157.58)-bPt-5419(161.91)	159.69	157.505-161.875	E32M62-386(158.61)-E38M55-139(158.80)			
MQTL4.6	4H	bPt-6055(157.58)-bPt-5419(161.91)	159.69	157.505-161.875	E32M62-386(158.61)-E38M55-139(158.80)			
MQTL4.6	4H	bPt-6055(157.58)-bPt-5419(161.91)	159.69	157.505-161.875	E32M62-386(158.61)-E38M55-139(158.80)			
MQTL4.6	4H	bPt-6055(157.58)-bPt-5419(161.91)	159.69	157.505-161.875	E32M62-386(158.61)-E38M55-139(158.80)			
MQTL4.6	4H	bPt-6055(157.58)-bPt-5419(161.91)	159.69	157.505-161.875	E32M62-386(158.61)-E38M55-139(158.80)			
MQTL4.6	4H	bPt-6055(157.58)-bPt-5419(161.91)	159.69	157.505-161.875	E32M62-386(158.61)-E38M55-139(158.80)			
MQTL4.6	4H	bPt-6055(157.58)-bPt-5419(161.91)	159.69	157.505-161.875	E32M62-386(158.61)-E38M55-139(158.80)			
MQTL4.6	4H	bPt-6055(157.58)-bPt-5419(161.91)	159.69	157.505-161.875	E32M62-386(158.61)-E38M55-139(158.80)			
MQTL4.6	4H	bPt-6055(157.58)-bPt-5419(161.91)	159.69	157.505-161.875	E32M62-386(158.61)-E38M55-139(158.80)			
MQTL4.6	4H	bPt-6055(157.58)-bPt-5419(161.91)	159.69	157.505-161.875	E32M62-386(158.61)-E38M55-139(158.80)			
MQTL4.7	4H	bags29m17(181.48)-MWG2257(183.72)	182.58	181.43-183.73	BF258346B(182.46)-bPb-7395(182.61)	14	5	5

Continued Table S3.

MQTL	Chromosome	Flanking marker	MQTL position	MQTL confidence interval(cM)	Closest marker	Number of initial QTLs	Number of studies	Number of populations
MQTL4.7	4H	bags29m17(181.48)-MWG2257(183.72)	182.58	181.43-183.73	BF258346B(182.46)-bPb-7395(182.61)	14	5	5
MQTL4.7	4H	bags29m17(181.48)-MWG2257(183.72)	182.58	181.43-183.73	BF258346B(182.46)-bPb-7395(182.61)			
MQTL4.7	4H	bags29m17(181.48)-MWG2257(183.72)	182.58	181.43-183.73	BF258346B(182.46)-bPb-7395(182.61)			
MQTL4.7	4H	bags29m17(181.48)-MWG2257(183.72)	182.58	181.43-183.73	BF258346B(182.46)-bPb-7395(182.61)			
MQTL4.7	4H	bags29m17(181.48)-MWG2257(183.72)	182.58	181.43-183.73	BF258346B(182.46)-bPb-7395(182.61)			
MQTL4.7	4H	bags29m17(181.48)-MWG2257(183.72)	182.58	181.43-183.73	BF258346B(182.46)-bPb-7395(182.61)			
MQTL4.7	4H	bags29m17(181.48)-MWG2257(183.72)	182.58	181.43-183.73	BF258346B(182.46)-bPb-7395(182.61)			
MQTL4.7	4H	bags29m17(181.48)-MWG2257(183.72)	182.58	181.43-183.73	BF258346B(182.46)-bPb-7395(182.61)			
MQTL4.7	4H	bags29m17(181.48)-MWG2257(183.72)	182.58	181.43-183.73	BF258346B(182.46)-bPb-7395(182.61)			
MQTL4.7	4H	bags29m17(181.48)-MWG2257(183.72)	182.58	181.43-183.73	BF258346B(182.46)-bPb-7395(182.61)			
MQTL4.7	4H	bags29m17(181.48)-MWG2257(183.72)	182.58	181.43-183.73	BF258346B(182.46)-bPb-7395(182.61)			
MQTL4.7	4H	bags29m17(181.48)-MWG2257(183.72)	182.58	181.43-183.73	BF258346B(182.46)-bPb-7395(182.61)			
MQTL4.7	4H	bags29m17(181.48)-MWG2257(183.72)	182.58	181.43-183.73	BF258346B(182.46)-bPb-7395(182.61)			
MQTL4.8	4H	ABC319C(208.25)-GBMS128a(210.67)	209.18	208.185-210.75	mHsh(209.15)-ABC305(209.24)	36	9	5
MQTL4.8	4H	ABC319C(208.25)-GBMS128a(210.67)	209.18	208.185-210.75	mHsh(209.15)-ABC305(209.24)			

Continued Table S3.

MQTL	Chromosome	Flanking marker	MQTL position	MQTL confidence interval(cM)	Closest marker	Number of initial QTLs	Number of studies	Number of populatio ns
MQTL4.8	4H	ABC319C(208.25)-GBMS128a(210.67)	209.18	208.185-210.75	mHsh(209.15)-ABC305(209.24)	36	9	5
MQTL4.8	4H	ABC319C(208.25)-GBMS128a(210.67)	209.18	208.185-210.75	mHsh(209.15)-ABC305(209.24)			
MQTL4.8	4H	ABC319C(208.25)-GBMS128a(210.67)	209.18	208.185-210.75	mHsh(209.15)-ABC305(209.24)			
MQTL4.8	4H	ABC319C(208.25)-GBMS128a(210.67)	209.18	208.185-210.75	mHsh(209.15)-ABC305(209.24)			
MQTL4.8	4H	ABC319C(208.25)-GBMS128a(210.67)	209.18	208.185-210.75	mHsh(209.15)-ABC305(209.24)			
MQTL4.8	4H	ABC319C(208.25)-GBMS128a(210.67)	209.18	208.185-210.75	mHsh(209.15)-ABC305(209.24)			
MQTL4.8	4H	ABC319C(208.25)-GBMS128a(210.67)	209.18	208.185-210.75	mHsh(209.15)-ABC305(209.24)			
MQTL4.8	4H	ABC319C(208.25)-GBMS128a(210.67)	209.18	208.185-210.75	mHsh(209.15)-ABC305(209.24)			
MQTL4.8	4H	ABC319C(208.25)-GBMS128a(210.67)	209.18	208.185-210.75	mHsh(209.15)-ABC305(209.24)			
MQTL4.8	4H	ABC319C(208.25)-GBMS128a(210.67)	209.18	208.185-210.75	mHsh(209.15)-ABC305(209.24)			
MQTL4.8	4H	ABC319C(208.25)-GBMS128a(210.67)	209.18	208.185-210.75	mHsh(209.15)-ABC305(209.24)			
MQTL4.8	4H	ABC319C(208.25)-GBMS128a(210.67)	209.18	208.185-210.75	mHsh(209.15)-ABC305(209.24)			
MQTL4.8	4H	ABC319C(208.25)-GBMS128a(210.67)	209.18	208.185-210.75	mHsh(209.15)-ABC305(209.24)			
MQTL4.8	4H	ABC319C(208.25)-GBMS128a(210.67)	209.18	208.185-210.75	mHsh(209.15)-ABC305(209.24)			
MQTL4.8	4H	ABC319C(208.25)-GBMS128a(210.67)	209.18	208.185-210.75	mHsh(209.15)-ABC305(209.24)			

Continued Table S3.

MQTL	Chromosome	Flanking marker	MQTL position	MQTL confidence interval(cM)	Closest marker	Number of initial QTLs	Number of studies	Number of populations
MQTL4.8	4H	ABC319C(208.25)-GBMS128a(210.67)	209.18	208.185-210.75	mHsh(209.15)-ABC305(209.24)	36	9	5
MQTL4.8	4H	ABC319C(208.25)-GBMS128a(210.67)	209.18	208.185-210.75	mHsh(209.15)-ABC305(209.24)			
MQTL4.8	4H	ABC319C(208.25)-GBMS128a(210.67)	209.18	208.185-210.75	mHsh(209.15)-ABC305(209.24)			
MQTL4.8	4H	ABC319C(208.25)-GBMS128a(210.67)	209.18	208.185-210.75	mHsh(209.15)-ABC305(209.24)			
MQTL4.8	4H	ABC319C(208.25)-GBMS128a(210.67)	209.18	208.185-210.75	mHsh(209.15)-ABC305(209.24)			
MQTL4.8	4H	ABC319C(208.25)-GBMS128a(210.67)	209.18	208.185-210.75	mHsh(209.15)-ABC305(209.24)			
MQTL4.8	4H	ABC319C(208.25)-GBMS128a(210.67)	209.18	208.185-210.75	mHsh(209.15)-ABC305(209.24)			
MQTL4.8	4H	ABC319C(208.25)-GBMS128a(210.67)	209.18	208.185-210.75	mHsh(209.15)-ABC305(209.24)			
MQTL4.8	4H	ABC319C(208.25)-GBMS128a(210.67)	209.18	208.185-210.75	mHsh(209.15)-ABC305(209.24)			
MQTL4.8	4H	ABC319C(208.25)-GBMS128a(210.67)	209.18	208.185-210.75	mHsh(209.15)-ABC305(209.24)			
MQTL4.8	4H	ABC319C(208.25)-GBMS128a(210.67)	209.18	208.185-210.75	mHsh(209.15)-ABC305(209.24)			
MQTL4.8	4H	ABC319C(208.25)-GBMS128a(210.67)	209.18	208.185-210.75	mHsh(209.15)-ABC305(209.24)			
MQTL4.8	4H	ABC319C(208.25)-GBMS128a(210.67)	209.18	208.185-210.75	mHsh(209.15)-ABC305(209.24)			
MQTL4.8	4H	ABC319C(208.25)-GBMS128a(210.67)	209.18	208.185-210.75	mHsh(209.15)-ABC305(209.24)			
MQTL4.8	4H	ABC319C(208.25)-GBMS128a(210.67)	209.18	208.185-210.75	mHsh(209.15)-ABC305(209.24)			

Continued Table S3.

MQTL	Chromosome	Flanking marker	MQTL position	MQTL confidence interval(cM)	Closest marker	Number of initial QTLs	Number of studies	Number of populations
MQTL4.8	4H	ABC319C(208.25)-GBMS128a(210.67)	209.18	208.185-210.75	mHsh(209.15)-ABC305(209.24)	36	9	5
MQTL4.8	4H	ABC319C(208.25)-GBMS128a(210.67)	209.18	208.185-210.75	mHsh(209.15)-ABC305(209.24)			
MQTL4.8	4H	ABC319C(208.25)-GBMS128a(210.67)	209.18	208.185-210.75	mHsh(209.15)-ABC305(209.24)			
MQTL4.8	4H	ABC319C(208.25)-GBMS128a(210.67)	209.18	208.185-210.75	mHsh(209.15)-ABC305(209.24)			
MQTL5.1	5H	Act8A(11.32)-bags22c02(11.32)	10.05	8.115-11.985	ABG464(10.90)-Act8A(11.32)	2	1	1
MQTL5.1	5H	Act8A(11.32)-bags22c02(11.32)	10.05	8.115-11.985	ABG464(10.90)-Act8A(11.32)			
MQTL5.2	5H	baak29b22(106.14)-ABG064(107.69)	106.97	106.175-107.765	bp3200(106.34)-E40M40-354(107.08)	17	2	2
MQTL5.2	5H	baak29b22(106.14)-ABG064(107.69)	106.97	106.175-107.765	bp3200(106.34)-E40M40-354(107.08)			
MQTL5.2	5H	baak29b22(106.14)-ABG064(107.69)	106.97	106.175-107.765	bp3200(106.34)-E40M40-354(107.08)			
MQTL5.2	5H	baak29b22(106.14)-ABG064(107.69)	106.97	106.175-107.765	bp3200(106.34)-E40M40-354(107.08)			
MQTL5.2	5H	baak29b22(106.14)-ABG064(107.69)	106.97	106.175-107.765	bp3200(106.34)-E40M40-354(107.08)			
MQTL5.2	5H	baak29b22(106.14)-ABG064(107.69)	106.97	106.175-107.765	bp3200(106.34)-E40M40-354(107.08)			
MQTL5.2	5H	baak29b22(106.14)-ABG064(107.69)	106.97	106.175-107.765	bp3200(106.34)-E40M40-354(107.08)			
MQTL5.2	5H	baak29b22(106.14)-ABG064(107.69)	106.97	106.175-107.765	bp3200(106.34)-E40M40-354(107.08)			
MQTL5.2	5H	baak29b22(106.14)-ABG064(107.69)	106.97	106.175-107.765	bp3200(106.34)-E40M40-354(107.08)			

Continued Table S3.

MQTL	Chromosome	Flanking marker	MQTL position	MQTL confidence interval(cM)	Closest marker	Number of initial QTLs	Number of studies	Number of populations
MQTL5.2	5H	baak29b22(106.14)-ABG064(107.69)	106.97	106.175-107.765	bp3200(106.34)-E40M40-354(107.08)	17	2	2
MQTL5.2	5H	baak29b22(106.14)-ABG064(107.69)	106.97	106.175-107.765	bp3200(106.34)-E40M40-354(107.08)			
MQTL5.2	5H	baak29b22(106.14)-ABG064(107.69)	106.97	106.175-107.765	bp3200(106.34)-E40M40-354(107.08)			
MQTL5.2	5H	baak29b22(106.14)-ABG064(107.69)	106.97	106.175-107.765	bp3200(106.34)-E40M40-354(107.08)			
MQTL5.2	5H	baak29b22(106.14)-ABG064(107.69)	106.97	106.175-107.765	bp3200(106.34)-E40M40-354(107.08)			
MQTL5.2	5H	baak29b22(106.14)-ABG064(107.69)	106.97	106.175-107.765	bp3200(106.34)-E40M40-354(107.08)			
MQTL5.2	5H	baak29b22(106.14)-ABG064(107.69)	106.97	106.175-107.765	bp3200(106.34)-E40M40-354(107.08)			
MQTL5.2	5H	baak29b22(106.14)-ABG064(107.69)	106.97	106.175-107.765	bp3200(106.34)-E40M40-354(107.08)			
MQTL5.3	5H	GBMS70(118.08)-BOPA1_ABC17741-1-2-493(121.90)	119.96	118.01-121.910	E42M55-350(119.80)-E37M50-70(120.07)	5	1	1
MQTL5.3	5H	GBMS70(118.08)-BOPA1_ABC17741-1-2-493(121.90)	119.96	118.01-121.910	E42M55-350(119.80)-E37M50-70(120.07)			
MQTL5.3	5H	GBMS70(118.08)-BOPA1_ABC17741-1-2-493(121.90)	119.96	118.01-121.910	E42M55-350(119.80)-E37M50-70(120.07)			
MQTL5.3	5H	GBMS70(118.08)-BOPA1_ABC17741-1-2-493(121.90)	119.96	118.01-121.910	E42M55-350(119.80)-E37M50-70(120.07)			
MQTL5.3	5H	GBMS70(118.08)-BOPA1_ABC17741-1-2-493(121.90)	119.96	118.01-121.910	E42M55-350(119.80)-E37M50-70(120.07)			
MQTL5.4	5H	bags30n07(131.75)-bp139(137)	134.24	131.75-136.73	Bmac282a(134.20)-E37M62-231(134.43)	9	4	2
MQTL5.4	5H	bags30n07(131.75)-bp139(137)	134.24	131.75-136.73	Bmac282a(134.20)-E37M62-231(134.43)			

Continued Table S3.

MQTL	Chromosome	Flanking marker	MQTL position	MQTL confidence interval(cM)	Closest marker	Number of initial QTLs	Number of studies	Number of populations
MQTL5.4	5H	bags30n07(131.75)-bp139(137)	134.24	131.75-136.73	Bmac282a(134.20)-E37M62-231(134.43)	9	4	2
MQTL5.4	5H	bags30n07(131.75)-bp139(137)	134.24	131.75-136.73	Bmac282a(134.20)-E37M62-231(134.43)			
MQTL5.4	5H	bags30n07(131.75)-bp139(137)	134.24	131.75-136.73	Bmac282a(134.20)-E37M62-231(134.43)			
MQTL5.4	5H	bags30n07(131.75)-bp139(137)	134.24	131.75-136.73	Bmac282a(134.20)-E37M62-231(134.43)			
MQTL5.4	5H	bags30n07(131.75)-bp139(137)	134.24	131.75-136.73	Bmac282a(134.20)-E37M62-231(134.43)			
MQTL5.4	5H	bags30n07(131.75)-bp139(137)	134.24	131.75-136.73	Bmac282a(134.20)-E37M62-231(134.43)			
MQTL5.4	5H	bags30n07(131.75)-bp139(137)	134.24	131.75-136.73	Bmac282a(134.20)-E37M62-231(134.43)			
MQTL5.5	5H	ABC302(154.23)-GBM1426(159.59)	156.92	154.265-159.575	1896-1435(156.80)-bags4p07(157.03)	1	1	1
MQTL5.6	5H	GBM1041(169.88)-bPb-6967(177.68)	173.99	170.165-177.815	Bmac0223(173.52)-CDO57B(174.12)	2	1	1
MQTL5.6	5H	GBM1041(169.88)-bPb-6967(177.68)	173.99	170.165-177.815	Bmac0223(173.52)-CDO57B(174.12)			
MQTL5.7	5H	BG309785(180.09)-bp5428(183.81)	182.05	180.21-183.890	MWG923(181.91)-GBM1227(182.46)	1	1	1
MQTL5.8	5H	bPb-3700(210.054)-GBS0712(213.35)	211.71	210.055-213.365	bPb-4988(211.63)-7523-440(211.80)	12	3	2
MQTL5.8	5H	bPb-3700(210.054)-GBS0712(213.35)	211.71	210.055-213.365	bPb-4988(211.63)-7523-440(211.80)			
MQTL5.8	5H	bPb-3700(210.054)-GBS0712(213.35)	211.71	210.055-213.365	bPb-4988(211.63)-7523-440(211.80)			
MQTL5.8	5H	bPb-3700(210.054)-GBS0712(213.35)	211.71	210.055-213.365	bPb-4988(211.63)-7523-440(211.80)			

Continued Table S3.

MQTL	Chromosome	Flanking marker	MQTL position	MQTL confidence interval(cM)	Closest marker	Number of initial QTLs	Number of studies	Number of populatio ns
MQTL5.8	5H	bPb-3700(210.054)-GBS0712(213.35)	211.71	210.055-213.365	bPb-4988(211.63)-7523-440(211.80)	12	3	2
MQTL5.8	5H	bPb-3700(210.054)-GBS0712(213.35)	211.71	210.055-213.365	bPb-4988(211.63)-7523-440(211.80)			
MQTL5.8	5H	bPb-3700(210.054)-GBS0712(213.35)	211.71	210.055-213.365	bPb-4988(211.63)-7523-440(211.80)			
MQTL5.8	5H	bPb-3700(210.054)-GBS0712(213.35)	211.71	210.055-213.365	bPb-4988(211.63)-7523-440(211.80)			
MQTL5.8	5H	bPb-3700(210.054)-GBS0712(213.35)	211.71	210.055-213.365	bPb-4988(211.63)-7523-440(211.80)			
MQTL5.8	5H	bPb-3700(210.054)-GBS0712(213.35)	211.71	210.055-213.365	bPb-4988(211.63)-7523-440(211.80)			
MQTL5.8	5H	bPb-3700(210.054)-GBS0712(213.35)	211.71	210.055-213.365	bPb-4988(211.63)-7523-440(211.80)			
MQTL5.8	5H	bPb-3700(210.054)-GBS0712(213.35)	211.71	210.055-213.365	bPb-4988(211.63)-7523-440(211.80)			
MQTL5.9	5H	bPb-1224(231.1)-E39M59-72(232.01)	231.6	231.225-231.975	dhn9(231.4)-bPb-6367(231.8)	9	4	3
MQTL5.9	5H	bPb-1224(231.1)-E39M59-72(232.01)	231.6	231.225-231.975	dhn9(231.4)-bPb-6367(231.8)			
MQTL5.9	5H	bPb-1224(231.1)-E39M59-72(232.01)	231.6	231.225-231.975	dhn9(231.4)-bPb-6367(231.8)			
MQTL5.9	5H	bPb-1224(231.1)-E39M59-72(232.01)	231.6	231.225-231.975	dhn9(231.4)-bPb-6367(231.8)			
MQTL5.9	5H	bPb-1224(231.1)-E39M59-72(232.01)	231.6	231.225-231.975	dhn9(231.4)-bPb-6367(231.8)			
MQTL5.9	5H	bPb-1224(231.1)-E39M59-72(232.01)	231.6	231.225-231.975	dhn9(231.4)-bPb-6367(231.8)			
MQTL5.9	5H	bPb-1224(231.1)-E39M59-72(232.01)	231.6	231.225-231.975	dhn9(231.4)-bPb-6367(231.8)			

Continued Table S3.

MQTL	Chromosome	Flanking marker	MQTL position	MQTL confidence interval(cM)	Closest marker	Number of initial QTLs	Number of studies	Number of populations
MQTL5.9	5H	bPb-1224(231.1)-E39M59-72(232.01)	231.6	231.225-231.975	dhn9(231.4)-bPb-6367(231.8)	9	4	3
MQTL5.9	5H	bPb-1224(231.1)-E39M59-72(232.01)	231.6	231.225-231.975	dhn9(231.4)-bPb-6367(231.8)			
MQTL6.1	6H	AWBMA36(35.59)-bPb-4754(41.19)	38.29	35.535-41.045	bPb- 8054(38.32)-bPb-9768(38.80)	6	4	4
MQTL6.1	6H	AWBMA36(35.59)-bPb-4754(41.19)	38.29	35.535-41.045	bPb- 8054(38.32)-bPb-9768(38.80)			
MQTL6.1	6H	AWBMA36(35.59)-bPb-4754(41.19)	38.29	35.535-41.045	bPb- 8054(38.32)-bPb-9768(38.80)			
MQTL6.1	6H	AWBMA36(35.59)-bPb-4754(41.19)	38.29	35.535-41.045	bPb- 8054(38.32)-bPb-9768(38.80)			
MQTL6.1	6H	AWBMA36(35.59)-bPb-4754(41.19)	38.29	35.535-41.045	bPb- 8054(38.32)-bPb-9768(38.80)			
MQTL6.1	6H	AWBMA36(35.59)-bPb-4754(41.19)	38.29	35.535-41.045	bPb- 8054(38.32)-bPb-9768(38.80)			
MQTL6.2	6H	ABC152A(53.56)-bPb-3746(64.44)	58.86	53.61-64.11	bPb-4555(58.80)-GBM1049(58.86)	2	2	2
MQTL6.2	6H	ABC152A(53.56)-bPb-3746(64.44)	58.86	53.61-64.11	bPb-4555(58.80)-GBM1049(58.86)			
MQTL6.3	6H	bPt-6063(74.62)-EBmac624(77.27)	75.86	74.505-77.215	Bmac297(75.84)-bPb-1116(75.92)	38	8	4
MQTL6.3	6H	bPt-6063(74.62)-EBmac624(77.27)	75.86	74.505-77.215	Bmac297(75.84)-bPb-1116(75.92)			
MQTL6.3	6H	bPt-6063(74.62)-EBmac624(77.27)	75.86	74.505-77.215	Bmac297(75.84)-bPb-1116(75.92)			
MQTL6.3	6H	bPt-6063(74.62)-EBmac624(77.27)	75.86	74.505-77.215	Bmac297(75.84)-bPb-1116(75.92)			
MQTL6.3	6H	bPt-6063(74.62)-EBmac624(77.27)	75.86	74.505-77.215	Bmac297(75.84)-bPb-1116(75.92)			

Continued Table S3.

MQTL	Chromosome	Flanking marker	MQTL position	MQTL confidence interval(cM)	Closest marker	Number of initial QTLs	Number of studies	Number of populations
MQTL6.3	6H	bPt-6063(74.62)-EBmac624(77.27)	75.86	74.505-77.215	Bmac297(75.84)-bPb-1116(75.92)	38	8	4
MQTL6.3	6H	bPt-6063(74.62)-EBmac624(77.27)	75.86	74.505-77.215	Bmac297(75.84)-bPb-1116(75.92)			
MQTL6.3	6H	bPt-6063(74.62)-EBmac624(77.27)	75.86	74.505-77.215	Bmac297(75.84)-bPb-1116(75.92)			
MQTL6.3	6H	bPt-6063(74.62)-EBmac624(77.27)	75.86	74.505-77.215	Bmac297(75.84)-bPb-1116(75.92)			
MQTL6.3	6H	bPt-6063(74.62)-EBmac624(77.27)	75.86	74.505-77.215	Bmac297(75.84)-bPb-1116(75.92)			
MQTL6.3	6H	bPt-6063(74.62)-EBmac624(77.27)	75.86	74.505-77.215	Bmac297(75.84)-bPb-1116(75.92)			
MQTL6.3	6H	bPt-6063(74.62)-EBmac624(77.27)	75.86	74.505-77.215	Bmac297(75.84)-bPb-1116(75.92)			
MQTL6.3	6H	bPt-6063(74.62)-EBmac624(77.27)	75.86	74.505-77.215	Bmac297(75.84)-bPb-1116(75.92)			
MQTL6.3	6H	bPt-6063(74.62)-EBmac624(77.27)	75.86	74.505-77.215	Bmac297(75.84)-bPb-1116(75.92)			
MQTL6.3	6H	bPt-6063(74.62)-EBmac624(77.27)	75.86	74.505-77.215	Bmac297(75.84)-bPb-1116(75.92)			
MQTL6.3	6H	bPt-6063(74.62)-EBmac624(77.27)	75.86	74.505-77.215	Bmac297(75.84)-bPb-1116(75.92)			
MQTL6.3	6H	bPt-6063(74.62)-EBmac624(77.27)	75.86	74.505-77.215	Bmac297(75.84)-bPb-1116(75.92)			
MQTL6.3	6H	bPt-6063(74.62)-EBmac624(77.27)	75.86	74.505-77.215	Bmac297(75.84)-bPb-1116(75.92)			
MQTL6.3	6H	bPt-6063(74.62)-EBmac624(77.27)	75.86	74.505-77.215	Bmac297(75.84)-bPb-1116(75.92)			
MQTL6.3	6H	bPt-6063(74.62)-EBmac624(77.27)	75.86	74.505-77.215	Bmac297(75.84)-bPb-1116(75.92)			

Continued Table S3.

MQTL	Chromosome	Flanking marker	MQTL position	MQTL confidence interval(cM)	Closest marker	Number of initial QTLs	Number of studies	Number of populations
MQTL6.3	6H	bPt-6063(74.62)-EBmac624(77.27)	75.86	74.505-77.215	Bmac297(75.84)-bPb-1116(75.92)	38	8	4
MQTL6.3	6H	bPt-6063(74.62)-EBmac624(77.27)	75.86	74.505-77.215	Bmac297(75.84)-bPb-1116(75.92)			
MQTL6.3	6H	bPt-6063(74.62)-EBmac624(77.27)	75.86	74.505-77.215	Bmac297(75.84)-bPb-1116(75.92)			
MQTL6.3	6H	bPt-6063(74.62)-EBmac624(77.27)	75.86	74.505-77.215	Bmac297(75.84)-bPb-1116(75.92)			
MQTL6.3	6H	bPt-6063(74.62)-EBmac624(77.27)	75.86	74.505-77.215	Bmac297(75.84)-bPb-1116(75.92)			
MQTL6.3	6H	bPt-6063(74.62)-EBmac624(77.27)	75.86	74.505-77.215	Bmac297(75.84)-bPb-1116(75.92)			
MQTL6.3	6H	bPt-6063(74.62)-EBmac624(77.27)	75.86	74.505-77.215	Bmac297(75.84)-bPb-1116(75.92)			
MQTL6.3	6H	bPt-6063(74.62)-EBmac624(77.27)	75.86	74.505-77.215	Bmac297(75.84)-bPb-1116(75.92)			
MQTL6.3	6H	bPt-6063(74.62)-EBmac624(77.27)	75.86	74.505-77.215	Bmac297(75.84)-bPb-1116(75.92)			
MQTL6.3	6H	bPt-6063(74.62)-EBmac624(77.27)	75.86	74.505-77.215	Bmac297(75.84)-bPb-1116(75.92)			
MQTL6.3	6H	bPt-6063(74.62)-EBmac624(77.27)	75.86	74.505-77.215	Bmac297(75.84)-bPb-1116(75.92)			
MQTL6.3	6H	bPt-6063(74.62)-EBmac624(77.27)	75.86	74.505-77.215	Bmac297(75.84)-bPb-1116(75.92)			
MQTL6.3	6H	bPt-6063(74.62)-EBmac624(77.27)	75.86	74.505-77.215	Bmac297(75.84)-bPb-1116(75.92)			
MQTL6.3	6H	bPt-6063(74.62)-EBmac624(77.27)	75.86	74.505-77.215	Bmac297(75.84)-bPb-1116(75.92)			
MQTL6.3	6H	bPt-6063(74.62)-EBmac624(77.27)	75.86	74.505-77.215	Bmac297(75.84)-bPb-1116(75.92)			

Continued Table S3.

MQTL	Chromo some	Flanking marker	MQTL position	MQTL confidence interval(cM)	Closest marker	Number of initial QTLs	Number of studies	Number of populations
MQTL6.3	6H	bPt-6063(74.62)-EBmac624(77.27)	75.86	74.505-77.215	Bmac297(75.84)-bPb-1116(75.92)	38	8	4
MQTL6.3	6H	bPt-6063(74.62)-EBmac624(77.27)	75.86	74.505-77.215	Bmac297(75.84)-bPb-1116(75.92)			
MQTL6.3	6H	bPt-6063(74.62)-EBmac624(77.27)	75.86	74.505-77.215	Bmac297(75.84)-bPb-1116(75.92)			
MQTL6.4	6H	EBmac607b(94.81)-af93(dhn8)(95.335)	95.04	94.745-95.335	4191-268(95.03)-E45M48e(93.05)	13	4	3
MQTL6.4	6H	EBmac607b(94.81)-af93(dhn8)(95.335)	95.04	94.745-95.335	4191-268(95.03)-E45M48e(93.05)			
MQTL6.4	6H	EBmac607b(94.81)-af93(dhn8)(95.335)	95.04	94.745-95.335	4191-268(95.03)-E45M48e(93.05)			
MQTL6.4	6H	EBmac607b(94.81)-af93(dhn8)(95.335)	95.04	94.745-95.335	4191-268(95.03)-E45M48e(93.05)			
MQTL6.4	6H	EBmac607b(94.81)-af93(dhn8)(95.335)	95.04	94.745-95.335	4191-268(95.03)-E45M48e(93.05)			
MQTL6.4	6H	EBmac607b(94.81)-af93(dhn8)(95.335)	95.04	94.745-95.335	4191-268(95.03)-E45M48e(93.05)			
MQTL6.4	6H	EBmac607b(94.81)-af93(dhn8)(95.335)	95.04	94.745-95.335	4191-268(95.03)-E45M48e(93.05)			
MQTL6.4	6H	EBmac607b(94.81)-af93(dhn8)(95.335)	95.04	94.745-95.335	4191-268(95.03)-E45M48e(93.05)			
MQTL6.4	6H	EBmac607b(94.81)-af93(dhn8)(95.335)	95.04	94.745-95.335	4191-268(95.03)-E45M48e(93.05)			
MQTL6.4	6H	EBmac607b(94.81)-af93(dhn8)(95.335)	95.04	94.745-95.335	4191-268(95.03)-E45M48e(93.05)			
MQTL6.4	6H	EBmac607b(94.81)-af93(dhn8)(95.335)	95.04	94.745-95.335	4191-268(95.03)-E45M48e(93.05)			
MQTL6.4	6H	EBmac607b(94.81)-af93(dhn8)(95.335)	95.04	94.745-95.335	4191-268(95.03)-E45M48e(93.05)			

Continued Table S3.

MQTL	Chromosome	Flanking marker	MQTL position	MQTL confidence interval(cM)	Closest marker	Number of initial QTLs	Number of studies	Number of populations
MQTL6.4	6H	EBmac607b(94.81)-af93(dhn8)(95.335)	95.04	94.745-95.335	4191-268(95.03)-E45M48e(93.05)	13	4	3
MQTL7.1	7H	AWBMA36(35.59)-bPb-4754(41.19)	76.96	74.135-79.785	bPb- 8054(38.32)-bPb-9768(38.80)	25	8	6
MQTL7.1	7H	AWBMA36(35.59)-bPb-4754(41.19)	76.96	74.135-79.785	bPb- 8054(38.32)-bPb-9768(38.80)			
MQTL7.1	7H	AWBMA36(35.59)-bPb-4754(41.19)	76.96	74.135-79.785	bPb- 8054(38.32)-bPb-9768(38.80)			
MQTL7.1	7H	AWBMA36(35.59)-bPb-4754(41.19)	76.96	74.135-79.785	bPb- 8054(38.32)-bPb-9768(38.80)			
MQTL7.1	7H	AWBMA36(35.59)-bPb-4754(41.19)	76.96	74.135-79.785	bPb- 8054(38.32)-bPb-9768(38.80)			
MQTL7.1	7H	AWBMA36(35.59)-bPb-4754(41.19)	76.96	74.135-79.785	bPb- 8054(38.32)-bPb-9768(38.80)			
MQTL7.1	7H	AWBMA36(35.59)-bPb-4754(41.19)	76.96	74.135-79.785	bPb- 8054(38.32)-bPb-9768(38.80)			
MQTL7.1	7H	AWBMA36(35.59)-bPb-4754(41.19)	76.96	74.135-79.785	bPb- 8054(38.32)-bPb-9768(38.80)			
MQTL7.1	7H	AWBMA36(35.59)-bPb-4754(41.19)	76.96	74.135-79.785	bPb- 8054(38.32)-bPb-9768(38.80)			
MQTL7.1	7H	AWBMA36(35.59)-bPb-4754(41.19)	76.96	74.135-79.785	bPb- 8054(38.32)-bPb-9768(38.80)			
MQTL7.1	7H	AWBMA36(35.59)-bPb-4754(41.19)	76.96	74.135-79.785	bPb- 8054(38.32)-bPb-9768(38.80)			
MQTL7.1	7H	AWBMA36(35.59)-bPb-4754(41.19)	76.96	74.135-79.785	bPb- 8054(38.32)-bPb-9768(38.80)			
MQTL7.1	7H	AWBMA36(35.59)-bPb-4754(41.19)	76.96	74.135-79.785	bPb- 8054(38.32)-bPb-9768(38.80)			
MQTL7.1	7H	AWBMA36(35.59)-bPb-4754(41.19)	76.96	74.135-79.785	bPb- 8054(38.32)-bPb-9768(38.80)			

Continued Table S3.

MQTL	Chromosome	Flanking marker	MQTL position	MQTL confidence interval(cM)	Closest marker	Number of initial QTLs	Number of studies	Number of populations
MQTL7.1	7H	AWBMA36(35.59)-bPb-4754(41.19)	76.96	74.135-79.785	bPb- 8054(38.32)-bPb-9768(38.80)	25	8	6
MQTL7.1	7H	AWBMA36(35.59)-bPb-4754(41.19)	76.96	74.135-79.785	bPb- 8054(38.32)-bPb-9768(38.80)			
MQTL7.1	7H	AWBMA36(35.59)-bPb-4754(41.19)	76.96	74.135-79.785	bPb- 8054(38.32)-bPb-9768(38.80)			
MQTL7.1	7H	AWBMA36(35.59)-bPb-4754(41.19)	76.96	74.135-79.785	bPb- 8054(38.32)-bPb-9768(38.80)			
MQTL7.1	7H	AWBMA36(35.59)-bPb-4754(41.19)	76.96	74.135-79.785	bPb- 8054(38.32)-bPb-9768(38.80)			
MQTL7.1	7H	AWBMA36(35.59)-bPb-4754(41.19)	76.96	74.135-79.785	bPb- 8054(38.32)-bPb-9768(38.80)			
MQTL7.1	7H	AWBMA36(35.59)-bPb-4754(41.19)	76.96	74.135-79.785	bPb- 8054(38.32)-bPb-9768(38.80)			
MQTL7.1	7H	AWBMA36(35.59)-bPb-4754(41.19)	76.96	74.135-79.785	bPb- 8054(38.32)-bPb-9768(38.80)			
MQTL7.1	7H	AWBMA36(35.59)-bPb-4754(41.19)	76.96	74.135-79.785	bPb- 8054(38.32)-bPb-9768(38.80)			
MQTL7.1	7H	AWBMA36(35.59)-bPb-4754(41.19)	76.96	74.135-79.785	bPb- 8054(38.32)-bPb-9768(38.80)			
MQTL7.1	7H	AWBMA36(35.59)-bPb-4754(41.19)	76.96	74.135-79.785	bPb- 8054(38.32)-bPb-9768(38.80)			
MQTL7.2	7H	ABC152A(53.56)-bPb-3746(64.44)	92.08	88.525-95.635	bPb-4555(58.80)-GBM1049(58.86)	6	3	2
MQTL7.2	7H	ABC152A(53.56)-bPb-3746(64.44)	92.08	88.525-95.635	bPb-4555(58.80)-GBM1049(58.86)			
MQTL7.2	7H	ABC152A(53.56)-bPb-3746(64.44)	92.08	88.525-95.635	bPb-4555(58.80)-GBM1049(58.86)			
MQTL7.2	7H	ABC152A(53.56)-bPb-3746(64.44)	92.08	88.525-95.635	bPb-4555(58.80)-GBM1049(58.86)			

Continued Table S3.

MQTL	Chromosome	Flanking marker	MQTL position	MQTL confidence interval(cM)	Closest marker	Number of initial QTLs	Number of studies	Number of populations
MQTL7.2	7H	ABC152A(53.56)-bPb-3746(64.44)	92.08	88.525-95.635	bPb-4555(58.80)-GBM1049(58.86)	6	3	2
MQTL7.2	7H	ABC152A(53.56)-bPb-3746(64.44)	92.08	88.525-95.635	bPb-4555(58.80)-GBM1049(58.86)			
MQTL7.3	7H	bPt-6063(74.62)-EBmac624(77.27)	100.55	97.545-103.555	Bmac297(75.84)-bPb-1116(75.92)	3	2	2
MQTL7.3	7H	bPt-6063(74.62)-EBmac624(77.27)	100.55	97.545-103.555	Bmac297(75.84)-bPb-1116(75.92)			
MQTL7.3	7H	bPt-6063(74.62)-EBmac624(77.27)	100.55	97.545-103.555	Bmac297(75.84)-bPb-1116(75.92)			
MQTL7.4	7H	EBmac607b(94.81)-af93(dhn8)(95.335)	111.21	107.49-114.930	4191-268(95.03)-E45M48e(93.05)	8	2	2
MQTL7.4	7H	EBmac607b(94.81)-af93(dhn8)(95.335)	111.21	107.49-114.930	4191-268(95.03)-E45M48e(93.05)			
MQTL7.4	7H	EBmac607b(94.81)-af93(dhn8)(95.335)	111.21	107.49-114.930	4191-268(95.03)-E45M48e(93.05)			
MQTL7.4	7H	EBmac607b(94.81)-af93(dhn8)(95.335)	111.21	107.49-114.930	4191-268(95.03)-E45M48e(93.05)			
MQTL7.4	7H	EBmac607b(94.81)-af93(dhn8)(95.335)	111.21	107.49-114.930	4191-268(95.03)-E45M48e(93.05)			
MQTL7.4	7H	EBmac607b(94.81)-af93(dhn8)(95.335)	111.21	107.49-114.930	4191-268(95.03)-E45M48e(93.05)			
MQTL7.4	7H	EBmac607b(94.81)-af93(dhn8)(95.335)	111.21	107.49-114.930	4191-268(95.03)-E45M48e(93.05)			
MQTL7.4	7H	EBmac607b(94.81)-af93(dhn8)(95.335)	111.21	107.49-114.930	4191-268(95.03)-E45M48e(93.05)			
MQTL7.5	7H	bPb-4597(122.19)-GBMS2193(127.07)	124.67	122.26-127.08	GBM1030(124.47)-bPb-1039(124.83)	7	2	1
MQTL7.5	7H	bPb-4597(122.19)-GBMS2193(127.07)	124.67	122.26-127.08	GBM1030(124.47)-bPb-1039(124.83)			

Continued Table S3.

MQTL	Chromo some	Flanking marker	MQTL position	MQTL confidence interval(cM)	Closest marker	Number of initial QTLs	Number of studies	Number of populations
MQTL7.5	7H	bPb-4597(122.19)-GBMS2193(127.07)	124.67	122.26-127.08	GBM1030(124.47)-bPb-1039(124.83)	7	2	1
MQTL7.5	7H	bPb-4597(122.19)-GBMS2193(127.07)	124.67	122.26-127.08	GBM1030(124.47)-bPb-1039(124.83)			
MQTL7.5	7H	bPb-4597(122.19)-GBMS2193(127.07)	124.67	122.26-127.08	GBM1030(124.47)-bPb-1039(124.83)			
MQTL7.5	7H	bPb-4597(122.19)-GBMS2193(127.07)	124.67	122.26-127.08	GBM1030(124.47)-bPb-1039(124.83)			
MQTL7.5	7H	bPb-4597(122.19)-GBMS2193(127.07)	124.67	122.26-127.08	GBM1030(124.47)-bPb-1039(124.83)			
MQTL7.6	7H	Bmac0167(129.63)-bah34b22(132.57)	131.1	129.655-132.545	BOPA1_5480-826 (131.10)-BOPA1_ ABC11989-1-2-148(131.14)	12	6	2
MQTL7.6	7H	Bmac0167(129.63)-bah34b22(132.57)	131.1	129.655-132.545	BOPA1_5480-826 (131.10)-BOPA1_ ABC11989-1-2-148(131.14)			
MQTL7.6	7H	Bmac0167(129.63)-bah34b22(132.57)	131.1	129.655-132.545	BOPA1_5480-826 (131.10)-BOPA1_ ABC11989-1-2-148(131.14)			
MQTL7.6	7H	Bmac0167(129.63)-bah34b22(132.57)	131.1	129.655-132.545	BOPA1_5480-826 (131.10)-BOPA1_ ABC11989-1-2-148(131.14)			
MQTL7.6	7H	Bmac0167(129.63)-bah34b22(132.57)	131.1	129.655-132.545	BOPA1_5480-826 (131.10)-BOPA1_ ABC11989-1-2-148(131.14)			
MQTL7.6	7H	Bmac0167(129.63)-bah34b22(132.57)	131.1	129.655-132.545	BOPA1_5480-826 (131.10)-BOPA1_ ABC11989-1-2-148(131.14)			
MQTL7.6	7H	Bmac0167(129.63)-bah34b22(132.57)	131.1	129.655-132.545	BOPA1_5480-826 (131.10)-BOPA1_ ABC11989-1-2-148(131.14)			
MQTL7.6	7H	Bmac0167(129.63)-bah34b22(132.57)	131.1	129.655-132.545	BOPA1_5480-826 (131.10)-BOPA1_ ABC11989-1-2-148(131.14)			
MQTL7.6	7H	Bmac0167(129.63)-bah34b22(132.57)	131.1	129.655-132.545	BOPA1_5480-826 (131.10)-BOPA1_ ABC11989-1-2-148(131.14)			
MQTL7.6	7H	Bmac0167(129.63)-bah34b22(132.57)	131.1	129.655-132.545	BOPA1_5480-826 (131.10)-BOPA1_ ABC11989-1-2-148(131.14)			

Continued Table S3.

MQTL	Chromosome	Flanking marker	MQTL position	MQTL confidence interval(cM)	Closest marker	Number of initial QTLs	Number of studies	Number of populations
MQTL7.6	7H	Bmac0167(129.63)-bah34b22(132.57)	131.1	129.655-132.545	BOPA1_5480-826 (131.10)-BOPA1_ ABC11989-1-2-148(131.14)	12	6	2
MQTL7.6	7H	Bmac0167(129.63)-bah34b22(132.57)	131.1	129.655-132.545	BOPA1_5480-826 (131.10)-BOPA1_ ABC11989-1-2-148(131.14)			
MQTL7.7	7H	baak42a24(137.08)-EBmac0764(143.08)	139.2	137.125-143.075	bp8365(138.93)-SCRI_RS_122512(139.52)	3	1	1
MQTL7.7	7H	baak42a24(137.08)-EBmac0764(143.08)	139.2	137.125-143.075	bp8365(138.93)-SCRI_RS_122512(139.52)			
MQTL7.7	7H	baak42a24(137.08)-EBmac0764(143.08)	139.2	137.125-143.075	bp8365(138.93)-SCRI_RS_122512(139.52)			
MQTL7.8	7H	SCRI_RS_187512(154.89)-PSR117(160.79)	157.68	154.51-160.85	bPb-5260(157.53)-Bmag0174b(157.88)	3	2	1
MQTL7.8	7H	SCRI_RS_187512(154.89)-PSR117(160.79)	157.68	154.51-160.85	bPb-5260(157.53)-Bmag0174b(157.88)			
MQTL7.8	7H	SCRI_RS_187512(154.89)-PSR117(160.79)	157.68	154.51-160.85	bPb-5260(157.53)-Bmag0174b(157.88)			
MQTL7.9	7H	fcga199(167.78)-bah52g15(174.23)	170.85	167.59-174.110	ABC305(170.76)-bPb-9269(171.07)	7	4	3
MQTL7.9	7H	fcga199(167.78)-bah52g15(174.23)	170.85	167.59-174.110	ABC305(170.76)-bPb-9269(171.07)			
MQTL7.9	7H	fcga199(167.78)-bah52g15(174.23)	170.85	167.59-174.110	ABC305(170.76)-bPb-9269(171.07)			
MQTL7.9	7H	fcga199(167.78)-bah52g15(174.23)	170.85	167.59-174.110	ABC305(170.76)-bPb-9269(171.07)			
MQTL7.9	7H	fcga199(167.78)-bah52g15(174.23)	170.85	167.59-174.110	ABC305(170.76)-bPb-9269(171.07)			
MQTL7.9	7H	fcga199(167.78)-bah52g15(174.23)	170.85	167.59-174.110	ABC305(170.76)-bPb-9269(171.07)			
MQTL7.9	7H	fcga199(167.78)-bah52g15(174.23)	170.85	167.59-174.110	ABC305(170.76)-bPb-9269(171.07)			

Continued Table S3.

MQTL	Chromo some	Flanking marker	MQTL position	MQTL confidence interval(cM)	Closest marker	Number of initial QTLs	Number of studies	Number of populations
MQTL7.10	7H	bPb-7739(184.68)-ABG652(186.01)	185.11	184.3-185.92	bPb-4129(185.06)-bp5141(185.26)	15	5	4
MQTL7.10	7H	bPb-7739(184.68)-ABG652(186.01)	185.11	184.3-185.92	bPb-4129(185.06)-bp5141(185.26)			
MQTL7.10	7H	bPb-7739(184.68)-ABG652(186.01)	185.11	184.3-185.92	bPb-4129(185.06)-bp5141(185.26)			
MQTL7.10	7H	bPb-7739(184.68)-ABG652(186.01)	185.11	184.3-185.92	bPb-4129(185.06)-bp5141(185.26)			
MQTL7.10	7H	bPb-7739(184.68)-ABG652(186.01)	185.11	184.3-185.92	bPb-4129(185.06)-bp5141(185.26)			
MQTL7.10	7H	bPb-7739(184.68)-ABG652(186.01)	185.11	184.3-185.92	bPb-4129(185.06)-bp5141(185.26)			
MQTL7.10	7H	bPb-7739(184.68)-ABG652(186.01)	185.11	184.3-185.92	bPb-4129(185.06)-bp5141(185.26)			
MQTL7.10	7H	bPb-7739(184.68)-ABG652(186.01)	185.11	184.3-185.92	bPb-4129(185.06)-bp5141(185.26)			
MQTL7.10	7H	bPb-7739(184.68)-ABG652(186.01)	185.11	184.3-185.92	bPb-4129(185.06)-bp5141(185.26)			
MQTL7.10	7H	bPb-7739(184.68)-ABG652(186.01)	185.11	184.3-185.92	bPb-4129(185.06)-bp5141(185.26)			
MQTL7.10	7H	bPb-7739(184.68)-ABG652(186.01)	185.11	184.3-185.92	bPb-4129(185.06)-bp5141(185.26)			
MQTL7.10	7H	bPb-7739(184.68)-ABG652(186.01)	185.11	184.3-185.92	bPb-4129(185.06)-bp5141(185.26)			
MQTL7.10	7H	bPb-7739(184.68)-ABG652(186.01)	185.11	184.3-185.92	bPb-4129(185.06)-bp5141(185.26)			
MQTL7.10	7H	bPb-7739(184.68)-ABG652(186.01)	185.11	184.3-185.92	bPb-4129(185.06)-bp5141(185.26)			
MQTL7.10	7H	bPb-7739(184.68)-ABG652(186.01)	185.11	184.3-185.92	bPb-4129(185.06)-bp5141(185.26)			
