# Supplementary material for: Mega Meta-QTLs: A Strategy for the Production of Golden Barley (Hordeum vulgare L.) Tolerant to Abiotic Stresses
Source: Genes (Basel). 2022 Nov 10;13(11):2087. doi: 10.3390/genes13112087 (PMC9690463; doi:10.3390/genes13112087)
Supplement: Supplementary file 1 [file genes-13-02087-s001.zip › Table S4.pdf]

Table S4. Gene name and their Descriptions as well as rice gene ontology

Meta-QTL	Gene	Interpro Description	Rice Genes ID	Rice Genes Description
MQTL1.1	HORVU.MOREX.r3.1HG0026470	Leucine-rich repeat	Os01t0170300	Serine/threonine protein kinase-related domain containing protein.
	HORVU.MOREX.r3.1HG0026830	Leucine-rich repeat	Os05t0170300	Leucine-rich repeat domain containing protein.
	HORVU.MOREX.r3.1HG0027000	PLAT/LH2 domain superfamily	Os05t0171200	Embryo-specific 3 family protein.
	HORVU.MOREX.r3.1HG0027030	Mitochondrial substrate/solute carrier	Os05t0171300	Similar to Plastidial ADP-glucose transporter.
	HORVU.MOREX.r3.1HG0027090	Glyoxalase/fosfomycin resistance/dioxygenase domain	Os05t0171900	Glyoxalase/bleomycin resistance protein/dioxygenase domain containing protein.
	HORVU.MOREX.r3.1HG0027120	Glyoxalase/fosfomycin resistance/dioxygenase domain	Os05t0171900	Glyoxalase/bleomycin resistance protein/dioxygenase domain containing protein.
	HORVU.MOREX.r3.1HG0027280	NPH3 domain	Os05t0178000	NPH3 domain containing protein.
	HORVU.MOREX.r3.1HG0027330	UDP-glucuronosyl/UDP-glucosyltransferase	Os10t0178500	UDP-glucuronosyl/UDP-glucosyltransferase family protein.
	HORVU.MOREX.r3.1HG0027400	Bicarbonate transporter, eukaryotic	Os05t0176800	Boron efflux transporter, Pollen germination and/or tube elongation
	HORVU.MOREX.r3.1HG0027490	-	Os01t0175100	Kv1.4 voltage-gated K+ channel family protein.
	HORVU.MOREX.r3.1HG0027640	-	Os01t0175100	Kv1.4 voltage-gated K+ channel family protein.
	HORVU.MOREX.r3.1HG0027930	DVL	Os05t0179100	DVL family protein.
MQTL1.4	HORVU.MOREX.r3.1HG0037960	Alpha crystallin/Hsp20 domain	Os10t0437900	Heat shock protein Hsp20 domain containing protein.
	HORVU.MOREX.r3.1HG0038100	GDSL lipase/esterase	Os10t0438600	Similar to Family II lipase EXL3.
	HORVU.MOREX.r3.1HG0038160	Zinc finger, RING-type	Os10t0438800	Similar to RING-H2 finger protein ATL2E.
MQTL1.5	HORVU.MOREX.r3.1HG0041230		Os10t0466800	Conserved hypothetical protein.
	HORVU.MOREX.r3.1HG0041280	Cellulose synthase	Os10t0467800	Secondary wall-specific cellulose synthase, Secondary cell wall formation
	HORVU.MOREX.r3.1HG0042110	PPPDE peptidase domain	Os10t0472400	Similar to Thioredoxin.
MQTL1.6	HORVU.MOREX.r3.1HG0048590		Os01t0960600	Hypothetical conserved gene.
	HORVU.MOREX.r3.1HG0048840	Bifunctional inhibitor/plant lipid transfer protein/seed storage helical domain	Os10t0505700	Similar to Nonspecific lipid-transfer protein 2 (nsLTP2) (7 kDa lipid transfer protein).
	HORVU.MOREX.r3.1HG0048880	Bifunctional inhibitor/plant lipid transfer protein/seed storage helical domain	Os10t0505700	Similar to Nonspecific lipid-transfer protein 2 (nsLTP2) (7 kDa lipid transfer protein).
	HORVU.MOREX.r3.1HG0049040	Low-temperature-induced 78kDa/65kDa	Os10t0505900	Conserved hypothetical protein.
	HORVU.MOREX.r3.1HG0049190	Multicopper oxidase, type 1	Os06t0104300	Similar to Pectinesterase-like protein.

Continued Table S4.

Meta-QTL	Gene	Interpro Description	Rice Genes ID	Rice Genes Description
MQTL1.6	HORVU.MOREX.r3.1HG0049480	Protein kinase domain	Os10t0516200	Serine/threonine protein kinase domain containing protein.
MQTL2.1	HORVU.MOREX.r3.2HG0112190	Ovate protein family, C-terminal	Os07t0679100	Conserved hypothetical protein.
	HORVU.MOREX.r3.2HG0112520	WEB family	Os07t0677900	Protein of unknown function DUF827, plant family protein.
	HORVU.MOREX.r3.2HG0112580	Plant peroxidase	Os05t0171200	Embryo-specific 3 family protein.
	HORVU.MOREX.r3.2HG0112640	Plant peroxidase	Os07t0677500	Similar to Peroxidase precursor (EC 1.11.1.7).
	HORVU.MOREX.r3.2HG0112650	Secretory peroxidase	Os07t0677500	Similar to Peroxidase precursor (EC 1.11.1.7).
	HORVU.MOREX.r3.2HG0112660	Plant peroxidase	Os07t0677500	Similar to Peroxidase precursor (EC 1.11.1.7).
	HORVU.MOREX.r3.2HG0112670	Plant peroxidase	Os07t0677100	Peroxidase.
	HORVU.MOREX.r3.2HG0112690	Plant peroxidase	Os07t0676900	Similar to Peroxidase (EC 1.11.1.7).
	HORVU.MOREX.r3.2HG0112840		Os07t0675200	Conserved hypothetical protein.
	HORVU.MOREX.r3.2HG0113010	Phylloplanin-like	Os07t0674400	Pollen Ole e 1 allergen/extensin domain containing protein.
	HORVU.MOREX.r3.2HG0113020	Phylloplanin-like	Os07t0674400	Pollen Ole e 1 allergen/extensin domain containing protein.
	HORVU.MOREX.r3.2HG0113290		Os07t0673801	Conserved hypothetical protein.
	HORVU.MOREX.r3.2HG0113490	Universal stress protein A family	Os07t0673400	Homologue of the bacterial universal stress protein family, Adaptation to submergence stress, Ethylenemediated stress adaptation
	HORVU.MOREX.r3.2HG0113520	Universal stress protein A family	Os07t0673400	Homologue of the bacterial universal stress protein family, Adaptation to submergence stress, Ethylenemediated stress adaptation
	HORVU.MOREX.r3.2HG0113610	UDP-glucuronosyl/UDP-glucosyltransferase	Os07t0672700	UDP-glucuronosyl/UDP-glucosyltransferase family protein.
MQTL2.2	HORVU.MOREX.r3.2HG0119090	Protein DMP	Os07t0645300	Protein of unknown function DUF679 family protein.
	HORVU.MOREX.r3.2HG0119270	Lipase, GDXG, putative histidine active site	Os06t0306600	Alpha/beta hydrolase fold-3 domain containing protein.
	HORVU.MOREX.r3.2HG0119510	Glutathione S-transferase, N-terminal	Os10t0530400	Similar to Glutathione-S-transferase Cla47.
	HORVU.MOREX.r3.2HG0119560	Cold-shock protein, DNA-binding	Os02t0121100	Zinc finger, CCHC-type domain containing protein.
	HORVU.MOREX.r3.2HG0119580	Cold-shock protein, DNA-binding	Os02t0121100	Zinc finger, CCHC-type domain containing protein.
	HORVU.MOREX.r3.2HG0119630	Plant peroxidase	Os07t0638600	Similar to Class III peroxidase 44.
	HORVU.MOREX.r3.2HG0119650	Plant peroxidase	Os07t0638600	Similar to Class III peroxidase 44.

Continued Table S4.

Meta-QTL	Gene	Interpro Description	Rice Genes ID	Rice Genes Description
MQTL2.2	HORVU.MOREX.r3.2HG0119860	GroES-like superfamily	Os07t0641700	Similar to 10 kDa chaperonin (Protein CPN10) (Protein groES).
	HORVU.MOREX.r3.2HG0120120	SAM dependent carboxyl methyltransferase	Os06t0242000	Similar to benzoate carboxyl methyltransferase.
	HORVU.MOREX.r3.2HG0120320	Plant peroxidase	Os03t0368000	Similar to Peroxidase 1.
	HORVU.MOREX.r3.2HG0120350	Plant peroxidase	Os07t0639000	Similar to Class III peroxidase 46.
	HORVU.MOREX.r3.2HG0120360	Plant peroxidase	Os07t0639000	Similar to Class III peroxidase 46.
	HORVU.MOREX.r3.2HG0120470	Plant peroxidase	Os07t0638600	Similar to Class III peroxidase 44.
	HORVU.MOREX.r3.2HG0120490	Plant peroxidase	Os07t0638600	Similar to Class III peroxidase 44.
	HORVU.MOREX.r3.2HG0120560	Plant peroxidase	Os07t0638600	Similar to Class III peroxidase 44.
	HORVU.MOREX.r3.2HG0120650	Plant peroxidase	Os07t0638600	Similar to Class III peroxidase 44.
MQTL2.3	HORVU.MOREX.r3.2HG0123830	Alpha/beta hydrolase fold-1	Os07t0622700	Alpha/beta hydrolase fold-1 domain containing protein.
	HORVU.MOREX.r3.2HG0124540	EF-hand domain	Os07t0619200	EF hand domain containing protein.
	HORVU.MOREX.r3.2HG0124770		No Hit Found	
	HORVU.MOREX.r3.2HG0124850	Dirigent protein	Os07t0617500	Plant disease resistance response protein family protein.
	HORVU.MOREX.r3.2HG0125630	von Willebrand factor, type A	No Hit Found	
	HORVU.MOREX.r3.2HG0125650	Six-bladed beta-propeller, TolB-like	Os07t0614000	Six-bladed beta-propeller, TolB-like domain containing protein.
MQTL2.4	HORVU.MOREX.r3.2HG0131100	Sugar/inositol transporter	Os07t0582500	Similar to Proton myo-inositol cotransporter.
	HORVU.MOREX.r3.2HG0131150	Sugar/inositol transporter	Os07t0582400	Similar to Proton myo-inositol cotransporter.
	HORVU.MOREX.r3.2HG0131170	Sugar/inositol transporter	Os07t0582400	Similar to Proton myo-inositol cotransporter.
	HORVU.MOREX.r3.2HG0131310	Domain of unknown function DUF4091	Os07t0581300	Conserved hypothetical protein.
	HORVU.MOREX.r3.2HG0131510	Cysteine peptidase, cysteine active site	Os07t0480900	Similar to Cysteine protease (Fragment).
MQTL2.5	HORVU.MOREX.r3.2HG0134940	Heat shock factor (HSF)-type, DNA-binding	Os02t0527300	Similar to Heat shock transcription factor 31 (Fragment).
	HORVU.MOREX.r3.2HG0135060	Major facilitator, sugar transporter-like	Os07t0562400	Major facilitator superfamily protein.
	HORVU.MOREX.r3.2HG0135120	F-box domain	Os07t0561300	Cyclin-like F-box domain containing protein.

Continued Table S4.

Meta-QTL	Gene	Interpro Description	Rice Genes ID	Rice Genes Description
MQTL2.5	HORVU.MOREX.r3.2HG0135210	Late embryogenesis abundant protein, LEA_2 subgroup	Os02t0538700	Harpin-induced 1 domain containing protein.
	HORVU.MOREX.r3.2HG0135240	Armadillo	Os07t0560300	Armadillo-like helical domain containing protein.
MQTL2.6	HORVU.MOREX.r3.2HG0137660		No Hit Found	
MQTL2.7	HORVU.MOREX.r3.2HG0145360	Plant peroxidase	Os07t0499500	Similar to Peroxidase 7 precursor (EC 1.11.1.7) (Atperox P7) (ATP30).
MQTL2.8	HORVU.MOREX.r3.2HG0148110		No Hit Found	
MQTL2.9	HORVU.MOREX.r3.2HG0194310		Os04t0597900	Similar to OSJNba0093F12.13 protein.
	HORVU.MOREX.r3.2HG0194580	High mobility group box domain	Os02t0258200	Similar to high mobility group family.
	HORVU.MOREX.r3.2HG0194630	Tyrosinase copper-binding domain	Os04t0624500	Polyphenol oxidase, Formation of black hull, Responsible for the phenol reaction (PHR) phenotype, (Nipponbare:PHR-negative)
	HORVU.MOREX.r3.2HG0194770	Pectinesterase inhibitor domain	Os02t0537000	Pectinesterase inhibitor domain containing protein.
	HORVU.MOREX.r3.2HG0194810		Os08t0192300	Conserved hypothetical protein.
	HORVU.MOREX.r3.2HG0195490		Os04t0618400	Similar to H0313F03.13 protein.
	HORVU.MOREX.r3.2HG0195500		Os04t0618400	Similar to H0313F03.13 protein.
	HORVU.MOREX.r3.2HG0195550	Glycosyl transferase, family 43	Os01t0675500	Similar to Glycoprotein-specific UDP-glucuronyltransferase-like protein.
MQTL3.3	HORVU.MOREX.r3.3HG0279730	Heavy metal-associated domain, HMA	Os01t0678800	Heavy metal transport/detoxification protein domain containing protein.
	HORVU.MOREX.r3.3HG0280060	Purine permease, plant	Os01t0680200	Purine permease, "Regulation of grain size, grain number and leaf elongation", Modulation of cytokinin transport
	HORVU.MOREX.r3.3HG0280250	O-acyltransferase, WSD1, N-terminal	Os01t0681000	Uncharacterised protein family UPF0089 domain containing protein.
	HORVU.MOREX.r3.3HG0280400	Glutamate synthase, alpha subunit, C-terminal	Os01t0681900	Glutamate synthetase, Primary ammonium ions assimilation in seedling roots, Development of tillers, Tolerance to nitrogen-limitation
	HORVU.MOREX.r3.3HG0280530	Histone H3/CENP-A	Os04t0419600	Histone H3.
	HORVU.MOREX.r3.3HG0280680	Microtubule-associated protein, MAP65/Ase1/PRC1	Os01t0685900	Similar to 65kD microtubule associated protein.
	HORVU.MOREX.r3.3HG0280910	Protein of unknown function DUF4228, plant	Os01t0686000	Similar to cDNA, clone: J100026I16, full insert sequence.
	HORVU.MOREX.r3.3HG0280920	UDP-glucuronosyl/UDP-glucosyltransferase	Os01t0686300	UDP-glucuronosyl/UDP-glucosyltransferase family protein.
	HORVU.MOREX.r3.3HG0280960	PCH1	Os01t0687300	Conserved hypothetical protein.
	HORVU.MOREX.r3.3HG0281000	Wall-associated receptor kinase, galacturonan-binding domain	Os01t0690800	Protein kinase, core domain containing protein.

Continued Table S4.

Meta-QTL	Gene	Interpro Description	Rice Genes ID	Rice Genes Description
MQTL3.3	HORVU.MOREX.r3.3HG0281310	Reticulon	Os01t0691400	Similar to seed maturation protein.
	HORVU.MOREX.r3.3HG0281390	RNA recognition motif domain	Os05t0364600	Similar to pre-mRNA-splicing factor SF2.
	HORVU.MOREX.r3.3HG0281700	Oil body-associated protein-like	Os01t0728700	Protein of unknown function DUF1264 family protein.
MQTL3.4	HORVU.MOREX.r3.3HG0286390	NTF2-like domain superfamily	Os01t0731100	Similar to Pathogen-related protein.
	HORVU.MOREX.r3.3HG0286770	Heat shock factor (HSF)-type, DNA-binding	Os01t0733200	Heat shock factor, Transcription factor, ABA-mediated salt stress tolerance, Response to osmotic stress
	HORVU.MOREX.r3.3HG0286890	UDP-glucuronosyl/UDP-glucosyltransferase	Os01t0734600	UDP-glucuronosyl/UDP-glucosyltransferase family protein.
	HORVU.MOREX.r3.3HG0287070	Late embryogenesis abundant protein, LEA_2 subgroup	Os01t0736500	Similar to harpin-induced protein.
	HORVU.MOREX.r3.3HG0287240	Nucleobase cation symporter 2 family	Os01t0759900	Similar to Permease 1.
MQTL3.5	HORVU.MOREX.r3.3HG0290220	Dynein light chain, type 1/2	Os02t0269200	Dynein light chain, type 1 family protein.
	HORVU.MOREX.r3.3HG0290240	Serpin family	Os01t0765400	Protease inhibitor I4, serpin, plant domain containing protein.
	HORVU.MOREX.r3.3HG0291370		Os12t0187800	Conserved hypothetical protein.
	HORVU.MOREX.r3.3HG0291420		Os01t0767600	Conserved hypothetical protein.
	HORVU.MOREX.r3.3HG0291590	Amino acid transporter, transmembrane domain	Os05t0424000	Amino acid permease, Transport of amino acids
MQTL3.6	HORVU.MOREX.r3.3HG0307240	Amino acid transporter, transmembrane domain	Os05t0424000	Amino acid permease, Transport of amino acids
	HORVU.MOREX.r3.3HG0307250	Oxoglutarate/iron-dependent dioxygenase	Os03t0856000	2OG-Fe(II) oxygenase domain containing protein.
	HORVU.MOREX.r3.3HG0307390	SWEET sugar transporter	Os01t0881300	MtN3 and saliva related transmembrane protein family protein.
	HORVU.MOREX.r3.3HG0307420	Zinc finger, RING-type	Os07t0479100	Zinc finger, RING/FYVE/PHD-type domain containing protein.
	HORVU.MOREX.r3.3HG0307740	Oxoglutarate/iron-dependent dioxygenase	Os07t0169600	2OG-Fe(II) oxygenase domain containing protein.
	HORVU.MOREX.r3.3HG0307760	Zinc finger, TAZ-type	Os01t0908200	Member of the Bric-a-Brac/Tramtrack/Broad (BTB) family, BT1/BT2 ortholog, Negative regulation of nitrate uptake and nitrogen use efficiency
	HORVU.MOREX.r3.3HG0308180	Zinc finger, TAZ-type	Os01t0908200	Member of the Bric-a-Brac/Tramtrack/Broad (BTB) family, BT1/BT2 ortholog, Negative regulation of nitrate uptake and nitrogen use efficiency
	HORVU.MOREX.r3.3HG0308190	Amino acid transporter, transmembrane domain	Os01t0908600	Proline (Pro) and γ -aminobutyric acid (GABA) transporter, Stress tolerance, Cadmium stress tolerance
	HORVU.MOREX.r3.3HG0308270		No Hit Found	
	HORVU.MOREX.r3.3HG0308560	Modifying wall lignin-1/2	Os07t0462200	Protein of unknown function DUF1218 family protein.

Continued Table S4.

Meta-QTL	Gene	Interpro Description	Rice Genes ID	Rice Genes Description
MQTL3.6	HORVU.MOREX.r3.3HG0308570	Protein of unknown function DUF868, plant	Os01t0909400	Protein of unknown function DUF868, plant family protein.
	HORVU.MOREX.r3.3HG0308630	Aldehyde dehydrogenase domain	Os01t0591000	Aldehyde/histidinol dehydrogenase domain containing protein.
	HORVU.MOREX.r3.3HG0308840	Glyceraldehyde 3-phosphate dehydrogenase, NAD(P) binding domain	Os08t0126300	Similar to Glyceraldehyde-3-phosphate dehydrogenase (Fragment).
	HORVU.MOREX.r3.3HG0309170	B3 DNA binding domain	Os06t0194400	Transcriptional factor B3 family protein.
	HORVU.MOREX.r3.3HG0309240	Cystatin domain	Os01t0915200	Similar to cysteine proteinase inhibitor B.
	HORVU.MOREX.r3.3HG0309490		Os05t0479700	NB-ARC domain containing protein.
	HORVU.MOREX.r3.3HG0309510		No Hit Found	
	HORVU.MOREX.r3.3HG0309870		Os01t0918400	Conserved hypothetical protein.
	HORVU.MOREX.r3.3HG0309880	At3g27210-like	Os01t0920100	Conserved hypothetical protein.
	HORVU.MOREX.r3.3HG0310380	Nucleotide-diphospho-sugar transferase	Os01t0921100	Hypothetical conserved gene.
	HORVU.MOREX.r3.3HG0310460	Sulfotransferase domain	Os08t0515000	Sulfotransferase domain domain containing protein.
	HORVU.MOREX.r3.3HG0310600	Sulfotransferase domain	Os08t0515000	Sulfotransferase domain domain containing protein.
	HORVU.MOREX.r3.3HG0310640	Transcription factor, TCP	Os01t0924400	Similar to Auxin-induced basic helix-loop-helix transcription factor.
	HORVU.MOREX.r3.3HG0311460	Small auxin-up RNA	Os01t0924966	Auxin responsive SAUR protein family protein.
	HORVU.MOREX.r3.3HG0311560	Exostosin-like	Os01t0926400	Similar to Pectin-glucuronyltransferase.
	HORVU.MOREX.r3.3HG0312020	Heavy metal-associated domain, HMA	Os01t0927300	Heavy metal transport/detoxification protein domain containing protein.
	HORVU.MOREX.r3.3HG0312250		Os11t0552500	Conserved hypothetical protein.
	HORVU.MOREX.r3.3HG0312410		Os07t0646300	Conserved hypothetical protein.
	HORVU.MOREX.r3.3HG0312510	Peptidase S10, serine carboxypeptidase	Os12t0257000	Serine carboxypeptidase I precursor (EC 3.4.16.5) (Carboxypeptidase C).
	HORVU.MOREX.r3.3HG0312580	F-box domain	Os08t0203800	Cyclin-like F-box domain containing protein.
	HORVU.MOREX.r3.3HG0312750	Glycoside hydrolase family 1	Os01t0930800	Glycoside hydrolase, family 1 protein.
	HORVU.MOREX.r3.3HG0312760	Alcohol dehydrogenase, zinc-type, conserved site	Os10t0194200	Similar to Mannitol dehydrogenase.
	HORVU.MOREX.r3.3HG0313010	Heparan-alpha-glucosaminide N-acetyltransferase, catalytic domain	Os01t0931100	Protein of unknown function DUF1624 domain containing protein.

Continued Table S4.

Meta-QTL	Gene	Interpro Description	Rice Genes ID	Rice Genes Description
MQTL3.6	HORVU.MOREX.r3.3HG0313060	Armadillo-like helical	Os01t0931200	Armadillo-type fold domain containing protein.
	HORVU.MOREX.r3.3HG0313070	Rapid ALkalinization Factor	Os01t0257100	Rapid ALkalinization Factor family protein.
	HORVU.MOREX.r3.3HG0313490	Glutathione S-transferase, N-terminal	Os01t0933900	Similar to Glutathione transferase III(B) (EC 2.5.1.18).
	HORVU.MOREX.r3.3HG0313600	Glutathione S-transferase, N-terminal	Os01t0933900	Similar to Glutathione transferase III(B) (EC 2.5.1.18).
	HORVU.MOREX.r3.3HG0313640	PsbP, C-terminal	Os01t0934400	Mog1/PsbP, alpha/beta/alpha sandwich domain containing protein.
	HORVU.MOREX.r3.3HG0313890	Mog1/PsbP, alpha/beta/alpha sandwich	Os11t0492800	Similar to Esterase PIR7A.
	HORVU.MOREX.r3.3HG0313900	Alpha/beta hydrolase fold-1	Os11t0492800	Similar to Esterase PIR7A.
	HORVU.MOREX.r3.3HG0313910	Alpha/beta hydrolase fold-1	Os05t0305900	Leucine-rich repeat receptor-like kinase, Regulation of salt stress tolerance
MQTL4.7	HORVU.MOREX.r3.4HG0379400	Protein kinase domain	Os03t0327600	D-type euonymus-related lectin (EUL), "Response to salt and drought stress, hormone treatment", Response to biotic treatment
	HORVU.MOREX.r3.4HG0379640	Ricin B, lectin domain	Os07t0679100	Conserved hypothetical protein.
	HORVU.MOREX.r3.4HG0379660	Ovate protein family, C-terminal	Os07t0684100	Similar to Thioredoxin-like 1.
	HORVU.MOREX.r3.4HG0379720	Thioredoxin domain	Os03t0283100	Similar to In2-1 protein.
MQTL4.8	HORVU.MOREX.r3.4HG0386180	Glutathione S-transferase, N-terminal	Os11t0633800	Cyclin-like F-box domain containing protein.
	HORVU.MOREX.r3.4HG0386520		Os03t0280750	Plant disease resistance response protein family protein.
	HORVU.MOREX.r3.4HG0386840	Dirigent protein	Os01t0347600	Ervatamin B (EC 3.4.22.-) (ERV-B).
MQTL5.1	HORVU.MOREX.r3.5HG0424540	Peptidase C1A, papain C-terminal	Os03t0656500	Node-expressed Cd transporter, Putative cation/calcium (Ca) exchanger, Cd accumulation in the grain
	HORVU.MOREX.r3.5HG0424600	Sodium/calcium exchanger membrane region	Os06t0475400	Glycosyltransferase AER61, uncharacterized domain containing protein.
	HORVU.MOREX.r3.5HG0424650	Glycosyltransferase 61	Os12t0623500	Similar to Cationic amino acid transporter-like protein.
	HORVU.MOREX.r3.5HG0425110	Amino acid/polyamine transporter I	Os11t0249000	NB-ARC domain containing protein.
	HORVU.MOREX.r3.5HG0425370	NB-ARC	Os01t0607900	Serine/threonine protein kinase-related domain containing protein.
	HORVU.MOREX.r3.5HG0425730	Protein kinase domain	Os12t0600100	Tetratricopeptide-like helical domain containing protein.
MQTL5.3	HORVU.MOREX.r3.5HG0446800	Tetratricopeptide repeat 1	Os12t0530000	Similar to Histone H2A.
	HORVU.MOREX.r3.5HG0446810	Histone H2A	Os12t0508200	Conserved hypothetical protein.

Continued Table S4.

Meta-QTL	Gene	Interpro Description	Rice Genes ID	Rice Genes Description
MQTL5.4	HORVU.MOREX.r3.5HG0448480	At5g43822-like	Os04t0464200	Betaine aldehyde dehydrogenase, Rice fragrance, Salt stress
MQTL6.1	HORVU.MOREX.r3.6HG0570900	Succinate semialdehyde dehydrogenase	Os03t0307300	Nicotianamine synthase 1 (EC 2.5.1.43) (S-adenosyl-L-methionine:S- adenosyl-L-methionine:S-adenosyl-methionine 3-amino-3- carboxypropyltransferase 1) (OsNAS1).
	HORVU.MOREX.r3.6HG0571820	Nicotianamine synthase	Os08t0167400	Hypothetical conserved gene.
	HORVU.MOREX.r3.6HG0572030	Protein of unknown function DUF247, plant	Os06t0604600	Conserved hypothetical protein.
MQTL6.2	HORVU.MOREX.r3.6HG0578430		Os02t0201000	Similar to 23.6 kDa heat shock protein, mitochondrial.
	HORVU.MOREX.r3.6HG0578520	Heat shock protein 21-like	Os02t0209400	Conserved hypothetical protein.
	HORVU.MOREX.r3.6HG0578630		Os02t0210900	Conserved hypothetical protein.
	HORVU.MOREX.r3.6HG0578740		Os02t0218800	Allene oxide synthase (CYP74A4), Fatty acid 9-/13-hydroperoxide lyase (CYP74C), Biosynthesis of jasmonic acid (JA), Plant defense
	HORVU.MOREX.r3.6HG0579680	Cytochrome P450	Os02t0219900	Similar to Epstein-Barr nuclear antigen-1 (EBNA-1).
	HORVU.MOREX.r3.6HG0579870	Casparian strip membrane protein	Os02t0223700	Protein of unknown function DUF3511 domain containing protein.
	HORVU.MOREX.r3.6HG0580120	Protein of unknown function DUF3511	Os02t0226200	HAD-superfamily subfamily IB hydrolase, hypothetical 1 protein.
	HORVU.MOREX.r3.6HG0580790	HAD hydrolase, subfamily IA, Pyridoxal phosphate phosphatase-like	No Hit Found	
	HORVU.MOREX.r3.6HG0580960		Os02t0591500	Meiotic processes
MQTL6.4	HORVU.MOREX.r3.6HG0590050	POLYCHOME/GIGAS CELL1	Os08t0502400	FAS1 domain domain containing protein.
MQTL7.1	HORVU.MOREX.r3.7HG0678940	FAS1 domain	No Hit Found	
	HORVU.MOREX.r3.7HG0679060		Os08t0509400	Similar to Amygdalin hydrolase isoform AH I precursor (EC 3.2.1.117).
	HORVU.MOREX.r3.7HG0679960	Glycoside hydrolase family 1	No Hit Found	
MQTL7.2	HORVU.MOREX.r3.7HG0683360		Os08t0529400	F-box domain, cyclin-like domain containing protein.
	HORVU.MOREX.r3.7HG0683600	F-box domain	Os08t0532700	Similar to Peroxidase 55 precursor (EC 1.11.1.7) (Atperox P55) (ATP20a).
	HORVU.MOREX.r3.7HG0684180	Plant peroxidase	Os02t0465900	Vacuolar antiporter-regulating protein, Compartmentation of Na⁺ into vacuole, Salt tolerance

Continued Table S4.

Meta-QTL	Gene	Interpro Description	Rice Genes ID	Rice Genes Description
MQTL7.3	HORVU.MOREX.r3.7HG0686880	Glutathione-specific gamma-glutamylcyclotransferase	Os08t0546100	Similar to protein binding protein.
	HORVU.MOREX.r3.7HG0686900	Ubiquitin-like domain	Os08t0546800	Class B heat shock factor, Negative regulation of drought and salt tolerance
	HORVU.MOREX.r3.7HG0686980	Heat shock factor (HSF)-type, DNA-binding	Os08t0546900	Similar to NC domain-containing protein.
	HORVU.MOREX.r3.7HG0687050	LRAT domain	Os08t0550400	Similar to RING-H2 finger protein ATL5P.
MQTL7.4	HORVU.MOREX.r3.7HG0687790	Zinc finger, RING-type	Os08t0556400	Zinc finger, DHHC-type domain containing protein.
	HORVU.MOREX.r3.7HG0688580	Palmitoyltransferase, DHHC domain	Os08t0556400	Zinc finger, DHHC-type domain containing protein.
	HORVU.MOREX.r3.7HG0689170	Inosine/uridine-preferring nucleoside hydrolase domain	Os08t0557900	Inosine/uridine-preferring nucleoside hydrolase domain containing protein.
MQTL7.5	HORVU.MOREX.r3.7HG0691850	Activator of Hsp90 ATPase homologue 1-like	Os08t0464000	Activator of Hsp90 ATPase homologue 1-like family protein.
MQTL7.9	HORVU.MOREX.r3.7HG0699630	EamA domain	Os06t0105700	Protein of unknown function DUF6, transmembrane domain containing protein.
	HORVU.MOREX.r3.7HG0700030	Proton-dependent oligopeptide transporter family	Os08t0155400	A member of the nitrate transporter 1/peptide transporter family, Regulation of N utilization and flowering
MQTL7.10	HORVU.MOREX.r3.7HG0703260	Protein of unknown function DUF3537	Os08t0128200	Protein of unknown function DUF3537 domain containing protein.
