# Supplementary material for: Mega Meta-QTLs: A Strategy for the Production of Golden Barley (Hordeum vulgare L.) Tolerant to Abiotic Stresses
Source: Genes (Basel). 2022 Nov 10;13(11):2087. doi: 10.3390/genes13112087 (PMC9690463; doi:10.3390/genes13112087)
Supplement: Supplementary file 1 [file genes-13-02087-s001.zip › Table S5.pdf]

Table S5. Expression of genes influencing tolerance and sensitivity to abiotic stresses associated with any metaQTL Genenvestigator database (Genes whose expression is significant are shown in pink)

Chr.	Meta-QTL	Gene	Shoot (35.5)	Root (35.5)	PEG (6h)	PEG (24)	NaCl (6H)	NaCl (24h)	PEG+NaCl (6h)	PEG+NaCl (24)
1H	MQTL1.1	HORVU.MOREX.r3.1HG0026470	0.0107716	-2.001269	-0.2309	-1.692795	0.0806794	0.1348156	-1.212253	-1.9176
		HORVU.MOREX.r3.1HG0026830	-0.510025	-0.169795	-0.494205	-2.022612	-0.36702	-0.211695	-0.632765	-1.882004
		HORVU.MOREX.r3.1HG0027000	-0.103846	-0.122061	1.4320899	4.9029633	1.3074957	2.5768016	3.4960123	4.968103
		HORVU.MOREX.r3.1HG0027030	0.3324387	-0.232054	0.403931	2.366019	0.2480666	1.356833	0.8621411	2.3038287
		HORVU.MOREX.r3.1HG0027090	1.3693189	0.0833632	-0.510672	2.3764756	-0.293058	1.4851829	1.8064765	2.7881085
		HORVU.MOREX.r3.1HG0027120	-0.27154	0.2849544	0.0301875	4.0415542	0.3973301	1.7589472	1.8819814	4.3086515
		HORVU.MOREX.r3.1HG0027280	-0.265457	-1.802187	-0.109433	-2.533258	-0.112881	-0.72089	-1.677431	-2.863792
		HORVU.MOREX.r3.1HG0027330	0.6666573	0.0852451	0.0121662	3.20059	0	0.7984849	0.704078	3.7589098
		HORVU.MOREX.r3.1HG0027400	-0.099856	-0.222184	0.5108861	2.4498743	0.1601428	0.7171822	1.3862823	2.0260019
		HORVU.MOREX.r3.1HG0027490	-1.190359	-0.704664	0.7548586	1.4537684	0.3643691	-0.208885	2.0394266	1.7620302
		HORVU.MOREX.r3.1HG0027640	-1.673702	0.8875932	-0.319085	2.3567147	-0.41623	-0.405675	1.3290127	2.0736368
		HORVU.MOREX.r3.1HG0027930	-0.306284	-1.112373	-1.121523	-3.453773	-1.062721	-1.577781	-2.328912	-3.586883
	MQTL1.4	HORVU.MOREX.r3.1HG0037960	-0.673149	-0.305022	-0.267355	-1.701578	-0.130303	-0.27783	-0.165679	-2.102421
		HORVU.MOREX.r3.1HG0038100	-0.24908	-0.955858	0.3747528	-2.006586	-0.078268	0.0019682	0.156491	-2.436463
		HORVU.MOREX.r3.1HG0038160	0.5733996	1.0187342	0.2710007	-2.343001	-0.032214	-0.226549	-0.549841	-2.692755
	MQTL1.5	HORVU.MOREX.r3.1HG0041230	-0.909524	-1.387621	-0.316548	-2.611753	0.1794406	-1.244678	0.2745888	-2.70787
		HORVU.MOREX.r3.1HG0041280	-0.375218	-1.286158	0.1060384	-2.438323	-0.257672	-0.179691	-0.001651	-2.47034
		HORVU.MOREX.r3.1HG0042110	-0.136572	-1.084658	0.5129515	-2.028972	-0.21688	-0.298125	-0.148528	-2.177475
	MQTL1.6	HORVU.MOREX.r3.1HG0048590	0.7773707	2.311974	0.162205	0.2794048	0.1056112	0.1411189	0.1834027	0.1442834
		HORVU.MOREX.r3.1HG0048840	-0.536992	-0.776143	-0.892786	-3.560402	-0.951453	-1.844164	-1.660347	-4.058494
		HORVU.MOREX.r3.1HG0048880	-0.149272	-0.685969	-0.023313	-1.968182	0.558916	0.499985	-0.005869	-2.319208
		HORVU.MOREX.r3.1HG0049040	0.6646409	2.006653	4.5139273	6.579664	4.4204699	5.3403475	7.111495	6.6270698
		HORVU.MOREX.r3.1HG0049190	-0.612446	-1.61441	-0.519258	-2.493813	-0.453078	-1.142673	-1.203198	-2.649317

Continued Table S5.

Chr.	Meta-QTL	Gene	Shoot (35.5)	Root (35.5)	PEG (6h)	PEG (24)	NaCl (6H)	NaCl (24h)	PEG+NaCl (6h)	PEG+NaCl (24)
		HORVU.MOREX.r3.1HG0049480	0.3374815	0.6358082	0.0541697	-2.229868	0.2411215	-0.33237	-1.086463	-2.767051
2H	MQTL2.1	HORVU.MOREX.r3.2HG0112190	-0.170066	-0.149513	-0.312326	-1.606649	-0.341716	0.0049675	-1.724219	-2.071548
		HORVU.MOREX.r3.2HG0112520	-0.108145	-2.382024	-0.394607	-0.937555	-0.276012	-0.111024	-1.32358	-1.632469
		HORVU.MOREX.r3.2HG0112580	-1.02763	-1.291993	-1.393201	-2.670248	-1.511259	0.1744529	-2.551979	-2.671823
		HORVU.MOREX.r3.2HG0112640	-2.024395	-1.274728	-1.943521	-7.431625	-1.652043	-3.36458	-5.032325	-7.40224
		HORVU.MOREX.r3.2HG0112650	0	-0.849065	-1.504434	-2.756213	-1.08334	-2.161547	-2.750612	-2.825005
		HORVU.MOREX.r3.2HG0112660	0.0357943	-1.6634	-1.620003	-4.983343	-0.833403	-0.604686	-3.305513	-5.15514
		HORVU.MOREX.r3.2HG0112670	0	-2.157216	-1.645558	-3.657161	-0.898027	-0.596157	-4.31374	-4.816743
		HORVU.MOREX.r3.2HG0112690	0	-1.877632	-1.718973	-3.376082	-0.317626	-0.195555	-3.626906	-3.95465
		HORVU.MOREX.r3.2HG0112840	3.9428479	8.1865858	0.9294517	5.4915405	0.2662271	2.6348061	3.7110587	6.2727106
		HORVU.MOREX.r3.2HG0113010	0.1054253	-0.738652	-0.049329	-3.127359	0.149963	-0.495087	-0.543151	-4.173271
		HORVU.MOREX.r3.2HG0113020	-0.440005	-0.838782	-0.244948	-3.586225	0.2614496	-0.742262	-0.527382	-3.20248
		HORVU.MOREX.r3.2HG0113290	0.049518	-1.956362	-0.56296	-2.718155	-0.219123	-1.128407	-1.713796	-3.039127
		HORVU.MOREX.r3.2HG0113490	1.501408	1.2563214	-0.288407	-1.447601	0.1185666	-0.240386	-0.534311	-2.066128
		HORVU.MOREX.r3.2HG0113520	-1.957283	-2.346455	-0.351267	-2.99151	-0.273092	-0.554664	-1.534032	-3.517776
		HORVU.MOREX.r3.2HG0113610	-0.028696	2.2551571	0.4827883	5.6924615	0.1073917	2.3788545	2.7008952	6.4845838
	MQTL2.2	HORVU.MOREX.r3.2HG0119090	-0.175423	-1.119017	-0.325728	-1.631434	0.5577315	0.5135782	-0.60512	-2.078684
		HORVU.MOREX.r3.2HG0119270	-2.27788	-0.236262	1.6737604	1.0696124	1.1994042	1.0283225	2.2699968	1.6728774
		HORVU.MOREX.r3.2HG0119510	0.7496179	4.1801944	0.1575161	-0.182035	0.1240238	0.1855661	0.3763139	-0.211939
		HORVU.MOREX.r3.2HG0119560	0	-0.605254	-1.016747	-2.290721	-0.275891	-0.168299	-3.213583	-4.520467
		HORVU.MOREX.r3.2HG0119580	0	-1.218349	-0.907534	-2.039669	-0.253689	-0.058032	-2.511081	-4.221964
		HORVU.MOREX.r3.2HG0119630	0.0539009	-2.786139	-0.977021	-2.766198	-0.056189	-0.319531	-2.544378	-4.931054
		HORVU.MOREX.r3.2HG0119650	0	-1.92947	-1.171244	-2.792438	-0.212063	-0.300258	-2.610751	-4.946939

Continued Table S5.

Chr.	Meta-QTL	Gene	Shoot (35.5)	Root (35.5)	PEG (6h)	PEG (24)	NaCl (6H)	NaCl (24h)	PEG+NaCl (6h)	PEG+NaCl (24)
		HORVU.MOREX.r3.2HG0119860	2.1242909	1.239427	-0.07367	-0.447987	-0.0821	-0.678865	-0.178873	-0.255474
		HORVU.MOREX.r3.2HG0120120	0	2.3307014	0.0158518	-0.660022	0.2826432	-0.563936	-0.374348	-0.7461
		HORVU.MOREX.r3.2HG0120320	-0.024497	-1.068485	-0.985305	-2.894199	-0.217903	-1.448623	-0.869129	-2.657824
		HORVU.MOREX.r3.2HG0120350	0	-1.821761	-1.158987	-3.190718	-0.25733	-1.380537	-1.504657	-2.711696
		HORVU.MOREX.r3.2HG0120360	0.0209158	-0.706347	-1.631291	-2.91528	-0.173162	-1.133763	-1.033738	-2.342545
		HORVU.MOREX.r3.2HG0120470	0	-0.970433	-0.793378	-1.605588	-0.36252	0.0654282	-2.44767	-4.094555
		HORVU.MOREX.r3.2HG0120490	0	-0.814675	-0.769994	-1.732285	-0.521619	0.2029043	-2.644381	-3.890598
		HORVU.MOREX.r3.2HG0120560	0	-0.79011	-0.642257	-2.132358	-0.568999	-0.108914	-2.28031	-4.216739
		HORVU.MOREX.r3.2HG0120650	0.0393549	-0.496421	-0.586109	-1.933205	0.1010502	0.2886482	-2.00342	-2.974432
	MQTL2.3	HORVU.MOREX.r3.2HG0123830	1.4998723	1.3634025	1.5791101	2.3944924	1.2318829	1.4255634	2.6568454	2.4302705
		HORVU.MOREX.r3.2HG0124540	-0.265513	-0.093833	-0.400934	-2.57086	0.1940885	-1.801925	-0.053022	-3.213282
		HORVU.MOREX.r3.2HG0124770	-0.321337	0.3056809	0.6730268	3.0743252	1.2688875	0.9588297	0.9129968	3.3918876
		HORVU.MOREX.r3.2HG0124850	-0.046199	-1.278803	-0.894002	-3.034538	-0.19616	-0.634673	-2.33186	-3.572488
		HORVU.MOREX.r3.2HG0125630	0.6948663	2.5420182	-0.180945	-0.107985	-0.43813	0.123974	-0.196062	-0.433692
		HORVU.MOREX.r3.2HG0125650	-0.950932	0.0469123	0.2014133	3.8383703	0.0489497	1.1613759	0.7160396	3.6197952
	MQTL2.4	HORVU.MOREX.r3.2HG0131100	-0.210071	-2.015531	-0.546313	-2.806112	-0.212844	-0.603328	-1.312055	-2.652688
		HORVU.MOREX.r3.2HG0131150	-1.381005	-1.547785	-0.057685	-2.212035	-0.15852	-0.444684	-0.888876	-2.550757
		HORVU.MOREX.r3.2HG0131170	-1.500697	-2.984138	-0.465507	-1.027974	-0.443338	0.0280544	-0.457391	-1.116652
		HORVU.MOREX.r3.2HG0131310	-0.040165	-0.290685	0.570606	3.5482839	0.382137	1.5404664	1.9118898	3.4213505
		HORVU.MOREX.r3.2HG0131510	0.0349904	-0.037105	0.280804	3.8213744	0.2237195	2.0555672	1.5020104	3.9462371
	MQTL2.5	HORVU.MOREX.r3.2HG0134940	-0.029821	-0.285074	1.3675485	1.4765448	0.2441306	0.2290423	2.3637976	2.7296064
		HORVU.MOREX.r3.2HG0135060	-2.920035	-0.812915	-5.079266	-5.850824	-5.198596	-5.25682	-5.497001	-5.945907
		HORVU.MOREX.r3.2HG0135120	2.0077784	4.0197708	1.298231	5.1941073	0.7776108	1.6483558	3.8288097	5.3945723

Continued Table S5.

Chr.	Meta-QTL	Gene	Shoot (35.5)	Root (35.5)	PEG (6h)	PEG (24)	NaCl (6H)	NaCl (24h)	PEG+NaCl (6h)	PEG+NaCl (24)
		HORVU.MOREX.r3.2HG0135210	-1.027842	-1.669412	-0.981627	-3.587652	-0.400807	-0.88108	-3.009561	-5.44839
		HORVU.MOREX.r3.2HG0135240	0.0142677	-0.026985	0.2229709	-1.573476	0.0206726	-0.000247	-0.894953	-2.004039
	MQTL2.6	HORVU.MOREX.r3.2HG0137660	-0.613567	-1.779147	-0.688057	-3.623506	-0.683883	-1.350365	-1.860383	-3.401023
	MQTL2.7	HORVU.MOREX.r3.2HG0145360	0.0169337	0.0467121	-0.506429	-1.485076	-0.329799	-0.036062	-1.658948	-2.962893
	MQTL2.8	HORVU.MOREX.r3.2HG0148110	-0.51895	-1.731608	0.0357438	-1.190273	-0.261761	0.1922248	-0.395565	-2.378682
	MQTL2.9	HORVU.MOREX.r3.2HG0194310	-0.511445	-2.606668	-0.172797	-0.046898	-0.321227	0.0624367	-0.453941	-0.413642
		HORVU.MOREX.r3.2HG0194580	-0.472659	-3.127953	-0.232407	-0.81534	0.0422582	0.0949442	-0.740523	-1.249466
		HORVU.MOREX.r3.2HG0194630	-0.219091	0.0679415	-0.663563	-2.702075	-0.519467	-1.605484	-1.972771	-2.906413
		HORVU.MOREX.r3.2HG0194770	2.4179524	0.758176	4.2356126	4.5467548	3.1239747	2.8144254	5.1102867	4.6030441
		HORVU.MOREX.r3.2HG0194810	2.4802156	0	0	0	0	0	0	0
		HORVU.MOREX.r3.2HG0195490	0	0.7721916	1.4532954	3.4058605	0.8963266	3.0579079	1.7678739	3.6826126
		HORVU.MOREX.r3.2HG0195500	-0.119659	-0.473747	0.393186	2.6918251	-0.141476	1.3518105	1.1753514	2.7274731
		HORVU.MOREX.r3.2HG0195550	-0.623817	-1.052773	-0.274196	-3.802308	0.008092	0.3294488	-1.362044	-4.616115
3H	MQTL3.3	HORVU.MOREX.r3.3HG0279730	-0.104266	-1.397471	-0.301436	-1.905291	-0.375181	-0.196015	-1.312818	-2.721187
		HORVU.MOREX.r3.3HG0280060	-0.056361	-2.794266	-0.648174	-3.593031	-0.458558	-0.036743	-1.460672	-3.906558
		HORVU.MOREX.r3.3HG0280250	-0.572644	-2.763887	-0.99298	-2.357988	-0.102813	-0.364959	-1.281621	-2.385235
		HORVU.MOREX.r3.3HG0280400	2.1469166	0.2057816	0.8211341	3.2795298	0.7500509	1.9284603	2.1598497	3.0921472
		HORVU.MOREX.r3.3HG0280530	-0.992462	-2.145126	-0.615901	-1.259886	-0.197946	0.2528375	-0.175555	-1.202656
		HORVU.MOREX.r3.3HG0280680	-0.549116	-3.114218	-0.580787	-1.328001	-0.153248	-0.683182	-1.660384	-2.3387
		HORVU.MOREX.r3.3HG0280910	-0.42376	-2.452892	-0.435703	-0.687775	-0.555154	0.022369	-1.142871	-1.124627
		HORVU.MOREX.r3.3HG0280920	-0.482755	0.1854644	-0.401194	-2.468892	-0.241658	-0.372614	-1.406899	-3.527805
		HORVU.MOREX.r3.3HG0280960	-0.108016	-1.248038	-0.406819	-2.359957	-0.388111	-0.264258	-1.194868	-2.641096
		HORVU.MOREX.r3.3HG0281000	-1.087602	-0.255901	1.5706302	4.4953979	0.8953444	1.9332422	2.8075117	4.3989431

Continued Table S5.

Chr.	Meta-QTL	Gene	Shoot (35.5)	Root (35.5)	PEG (6h)	PEG (24)	NaCl (6H)	NaCl (24h)	PEG+NaCl (6h)	PEG+NaCl (24)
		HORVU.MOREX.r3.3HG0281310	-0.718726	-1.513563	-0.476311	-1.728445	-0.026468	0.4540559	-1.164423	-2.170216
		HORVU.MOREX.r3.3HG0281390	-0.768205	-0.193738	-0.193637	-1.785373	-0.48558	-0.680086	-0.646761	-2.284366
		HORVU.MOREX.r3.3HG0281700	2.0148089	3.4156785	1.1437711	2.9049535	0.8205624	1.6176361	2.3647979	2.9730842
	MQTL3.4	HORVU.MOREX.r3.3HG0286390	-0.250563	-0.01209	2.2918367	6.4424224	1.5199033	4.7796854	5.7697769	6.7310311
		HORVU.MOREX.r3.3HG0286770	-0.465711	-2.148133	0.0676004	-3.394653	0.1260806	-0.548093	-1.014157	-4.261017
		HORVU.MOREX.r3.3HG0286890	-0.263546	0.205004	4.2784965	6.3953443	3.1720415	3.9615535	6.806514	6.8409169
		HORVU.MOREX.r3.3HG0287070	2.1831716	1.1273315	-0.231951	-0.646339	-0.83707	0.1727994	-1.080108	-0.397205
		HORVU.MOREX.r3.3HG0287240	-0.072951	2.8011636	0.2649436	-0.362553	0.6066037	0.1232844	0.461847	-0.139493
	MQTL3.5	HORVU.MOREX.r3.3HG0290220	-0.719236	-2.43999	-0.648315	-1.661311	-0.600312	-0.311766	-1.795938	-2.244664
		HORVU.MOREX.r3.3HG0290240	-0.001821	0.0251239	-0.743207	-2.926625	-0.881231	-0.750271	-2.987525	-3.934644
		HORVU.MOREX.r3.3HG0291370	-0.018535	0.2275079	0.3003204	2.0079667	0.182013	1.1142417	2.064938	2.5133438
		HORVU.MOREX.r3.3HG0291420	0.1512429	-0.559998	4.0441397	6.7493508	1.7988725	4.4158579	7.0161274	7.1033419
		HORVU.MOREX.r3.3HG0291590	2.1862433	2.9349651	-0.108448	-0.616399	-0.033486	-0.052947	0.0298314	-0.715461
	MQTL3.6	HORVU.MOREX.r3.3HG0307240	0.0048986	-0.58828	-0.458951	-1.932479	-0.063078	-0.164038	-0.942972	-2.970798
		HORVU.MOREX.r3.3HG0307250	0.1010774	-1.129442	-0.344254	-1.703857	-0.100311	0.1948037	-0.975838	-2.901629
		HORVU.MOREX.r3.3HG0307390	0.1158778	1.0173465	1.0304622	-0.314966	0.9241844	0.8517218	2.086858	-0.19262
		HORVU.MOREX.r3.3HG0307420	-1.486238	-1.254991	-0.011478	-2.363989	-0.407441	-0/254333	-1.02275	-2.986471
		HORVU.MOREX.r3.3HG0307740	1.4337806	4.058953	0.2672411	-1.142789	0.2618617	0.2357084	-0.773132	-1.601937
		HORVU.MOREX.r3.3HG0307760	-1.058765	-0.723435	-0.46048	-3.07448	-0.307249	-0.581568	-0.661145	-2.458986
		HORVU.MOREX.r3.3HG0308180	-1.358071	-3.266991	0.332331	-0.864401	1.1381094	0.2919019	0.3621579	-0.942034
		HORVU.MOREX.r3.3HG0308190	-1.596413	-2.576809	-0.956831	-0.734286	0.0932526	0.4088717	-1.010452	-1.019683
		HORVU.MOREX.r3.3HG0308270	-0.308675	-0.744626	-0.58227	-2.053579	-0.214405	-0.429349	-1.469277	-2.190086
		HORVU.MOREX.r3.3HG0308560	-0.174693	-1.611327	-0.308768	-1.73068	-0.096148	-0.680416	-1.382952	-2.64709

Continued Table S5.

Chr.	Meta-QTL	Gene	Shoot (35.5)	Root (35.5)	PEG (6h)	PEG (24)	NaCl (6H)	NaCl (24h)	PEG+NaCl (6h)	PEG+NaCl (24)
	MQTL3.6	HORVU.MOREX.r3.3HG0308570	-0.51631	-1.647872	-0.246342	-2.42038	-0.083974	-0.65348	-1.089897	-2.940371
		HORVU.MOREX.r3.3HG0308630	-1.301754	-0.640295	-0.458176	-2.071652	-0.173274	-0.486965	-0.791692	-2.116166
		HORVU.MOREX.r3.3HG0308840	-0.525874	-0.712578	0.0259927	-1.713279	-0.457889	-0.303961	-0.462078	-2.607048
		HORVU.MOREX.r3.3HG0309510	0	-2.873251	-0.536137	-2.396739	-0.482097	-1.843335	-0.50654	-2.377221
		HORVU.MOREX.r3.3HG0309870	1.5243095	5.6963921	0	0.489963	0	0.1345023	0	0.712942
		HORVU.MOREX.r3.3HG0309880	3.1516572	4.905229	0.0442994	1.352247	0.2692748	0.8032326	0.5392566	1.3735826
		HORVU.MOREX.r3.3HG0310380	-0.647678	-1.740696	-0.452695	-2.152799	-0.469941	-0.669178	-0.902605	-2.03663
		HORVU.MOREX.r3.3HG0310460	-0.221928	-1.640618	-0.519881	-2.437782	-0.695265	-0.466454	-1.46739	-2.650032
		HORVU.MOREX.r3.3HG0310600	0.023284	-2.502156	-0.532129	-2.791858	0.0200421	0.1494426	-2.140627	-3.104373
		HORVU.MOREX.r3.3HG0310640	0.0215811	-1.665366	-1.213631	-3.421077	-0.736806	-1.480399	-1.489642	-3.508247
		HORVU.MOREX.r3.3HG0311460	-2.032218	-0.663185	-0.242412	-0.33777	-0.115697	0.0210292	-0.022383	-0.241463
		HORVU.MOREX.r3.3HG0311560	0.0481588	-0.309536	-0.729445	-1.920407	-0.721872	-1.838401	-0.776876	-2.032572
		HORVU.MOREX.r3.3HG0312020	0.1936354	-0.939538	-0.333042	-1.84295	-0.186313	-0.684295	-1.037513	-2.006046
		HORVU.MOREX.r3.3HG0312250	-0.462187	-1.686833	-0.266709	-2.169408	-0.070227	-0.465186	-1.47206	-2.077094
		HORVU.MOREX.r3.3HG0312410	0.2168819	0.4917122	1.7309123	1.8903428	0.9445498	1.3705804	2.4258273	2.3333585
		HORVU.MOREX.r3.3HG0312510	0.3727875	-0.985855	-0.265906	-1.439111	-0.39282	0.0299102	-0.780092	-2.125418
		HORVU.MOREX.r3.3HG0312580	-0.793173	-1.266208	0.1150682	-1.477363	-0.24393	0.1962465	-0.290162	-2.283085
		HORVU.MOREX.r3.3HG0312750	-1.194229	-1.777769	-0.323654	-2.045284	-0.905071	-0.45452	-1.153972	-2.714092
		HORVU.MOREX.r3.3HG0312760	-2.660128	-0.095917	0	0.0425108	0	0.0646671	0	0
		HORVU.MOREX.r3.3HG0313010	0.0172386	-1.456227	-1.197331	-3.459967	-0.942854	-0.399446	-1.596936	-3.237367
		HORVU.MOREX.r3.3HG0313060	0.5614154	-0.119935	0.3205453	-1.320466	0.1435517	0.040232	-0.591515	-2.20596
		HORVU.MOREX.r3.3HG0313070	-0.468364	-2.736718	-0.562413	-2.548763	-0.22864	-0.629943	-1.642288	-2.59085
		HORVU.MOREX.r3.3HG0313490	-0.872736	-1.49298	-0.400341	-3.195943	0.3172712	-1.151113	-1.567782	-2.948388

Continued Table S5.

Chr.	Meta-QTL	Gene	Shoot (35.5)	Root (35.5)	PEG (6h)	PEG (24)	NaCl (6H)	NaCl (24h)	PEG+NaCl (6h)	PEG+NaCl (24)
		HORVU.MOREX.r3.3HG0313600	-0.40655	-0.69362	-0.434649	-2.358992	-0.26619	-0.564414	-1.115621	-2.441213
		HORVU.MOREX.r3.3HG0313640	-0.103225	-1.403869	-0.426675	-2.107606	-0.297055	-0.354427	-1.260035	-2.483265
		HORVU.MOREX.r3.3HG0313890	1.0072494	2.0701252	0.0558239	1.4386126	0.7516242	0.3580244	1.3548195	1.7573627
		HORVU.MOREX.r3.3HG0313900	-0.159204	-1.238673	-0.540791	-2.223214	-0.329208	-0.590407	-1.485825	-2.415544
		HORVU.MOREX.r3.3HG0313910	-0.33873	-0.185135	-0.39188	-2.404127	0.0008371	-1.193078	-0.492293	-2.263913
4H	MQTL4.7	HORVU.MOREX.r3.4HG0379400	-0.484989	-0.95294	-0/28457	-1.995176	-0.363789	-0.133971	-0.897139	-2.67228
		HORVU.MOREX.r3.4HG0379640	0.5621671	0.122054	1.7859797	2.8675101	1.3812609	2.3373614	2.5005387	2.7472501
		HORVU.MOREX.r3.4HG0379660	0.0229229	-0.135562	-0.551162	-1.562592	-0.01925	-0.075301	-1.714944	-2.255815
		HORVU.MOREX.r3.4HG0379720	1.179148	1.2627757	0.9574156	2.6708334	0.6839447	1.4413546	2.3732526	2.5436116
	MQTL4.8	HORVU.MOREX.r3.4HG0386180	1.8094834	2.6430546	0.5841968	2.2000806	0.0053324	1.1861893	1.3345867	1.875481
		HORVU.MOREX.r3.4HG0386520	-0.133729	-1.577192	-0.103882	-1.798689	-0.08507	0.206588	-1.448856	-2.897824
		HORVU.MOREX.r3.4HG0386840	-0.31936	-0.568363	0.1767271	-2.415813	0.5471404	0.1695845	0.4430253	-1.560773
5H	MQTL5.1	HORVU.MOREX.r3.5HG0424540	0	-3.565279	0.0196002	-0.069961	0.0190108	-0.069961	0	-0.069961
		HORVU.MOREX.r3.5HG0424600	-0.592215	1.1269415	0.0046935	2.2331416	-0.310489	0.9027861	1.6308515	2.5393902
		HORVU.MOREX.r3.5HG0424650	0.0299267	-0.394201	1.1455943	3.0216131	0.4410313	0.9791095	1.6010504	3.1913866
		HORVU.MOREX.r3.5HG0425110	-0.586886	-0.461069	-0.3617	-1.608398	-0.507682	0.1735946	-1.283473	-2.202176
		HORVU.MOREX.r3.5HG0425370	0	-1.832952	-0.498626	-1.257503	-0.25864	0.389052	-1.716375	-2.089611
		HORVU.MOREX.r3.5HG0425730	-0.388984	-1.085589	-0.191182	-2.093283	0.0062534	-0.453587	-0.775889	-2.258648
	MQTL5.3	HORVU.MOREX.r3.5HG0446800	0.4257892	-0.254795	1.0367069	2.1684589	0.5908226	0.921928	1.4622004	2.0449502
		HORVU.MOREX.r3.5HG0446810	2.9061534	2.3193289	-0.334842	-0.509159	-0.142535	-0.306887	-0.732369	-0.57823
	MQTL5.4	HORVU.MOREX.r3.5HG0448480	-0.494785	-1.488895	-0.349248	-1.900025	-0.27773	0.0160454	-1.225002	-2.476052
6H	MQTL6.1	HORVU.MOREX.r3.6HG0570900	0.3703207	-0.536074	0.6247702	2.8555296	0.4807792	1.7039016	1.5939977	2.6566421
		HORVU.MOREX.r3.6HG0571820	-0.223265	-2.185357	-0.265917	-1.649339	-0.079122	-1.368949	-0.138764	-1.472048

Continued Table S5.

Chr.	Meta-QTL	Gene	Shoot (35.5)	Root (35.5)	PEG (6h)	PEG (24)	NaCl (6H)	NaCl (24h)	PEG+NaCl (6h)	PEG+NaCl (24)
		HORVU.MOREX.r3.6HG0570900	0.3703207	-0.536074	0.6247702	2.8555296	0.4807792	1.7039016	1.5939977	2.6566421
		HORVU.MOREX.r3.6HG0571820	-0.223265	-2.185357	-0.265917	-1.649339	-0.079122	-1.368949	-0.138764	-1.472048
		HORVU.MOREX.r3.6HG0572030	0.01115	-1.381777	-1.51645	-2.834973	-0.963961	-1.324714	-1.801099	-2.89619
	MQTL6.2	HORVU.MOREX.r3.6HG0578430	0.7610421	-0.426527	1.4054179	1.1603089	1.4590462	1.402982	2.0561819	1.1687113
		HORVU.MOREX.r3.6HG0578520	1.2501917	5.7134819	0	0.1124664	0	0	0.1151863	0.1752109
		HORVU.MOREX.r3.6HG0578630	0.3633733	1.3481738	0.0185721	1.8942226	-0.031211	0.8986599	0.4590713	2.6907793
		HORVU.MOREX.r3.6HG0578740	0.0935871	0.7983094	-0.085223	-2.844799	0.3776388	-0.33945	-0.348657	-2.854085
		HORVU.MOREX.r3.6HG0579680	-0.320511	-2.266744	-0.507477	-2.534099	-0.494457	-0.729193	-1.37446	-2.708397
		HORVU.MOREX.r3.6HG0579870	0.3872752	-0.323319	1.1064247	2.1146922	1.366631	1.6156995	2.4568997	2.380205
		HORVU.MOREX.r3.6HG0580120	-0.759451	-2.501184	0.4675009	0.1777432	0.5244954	0.7563275	1.4454429	0.0932036
		HORVU.MOREX.r3.6HG0580790	-0.032313	-0.580345	-0.149976	-3.064888	-0.219822	-0.608979	-1.950434	-3.383863
		HORVU.MOREX.r3.6HG0580960	0.421533	1.1955592	-0.242682	-2.031917	-0.609025	-0.542708	-0.630862	-1.755475
	MQTL6.4	HORVU.MOREX.r3.6HG0590050	0.0899854	-1.244293	-0.560363	-1.302124	-0.32977	-0.290532	-1.612123	-2.096182
7H	MQTL7.1	HORVU.MOREX.r3.7HG0678940	-0.805933	-1.932333	-0.223026	-2.620229	0.0950535	-0.420782	-1.753151	-3.217196
		HORVU.MOREX.r3.7HG0679060	-0.278679	-0.134251	-0.738514	-1.605268	-0.703093	-0.473553	-0.968931	-2.01917
		HORVU.MOREX.r3.7HG0679960	-0.283712	0.4604681	-0.013982	-2.322286	-0.288761	-1.050089	-1.244292	-3.169151
	MQTL7.2	HORVU.MOREX.r3.7HG0683360	-0.451801	-0.534809	1.1857144	2.9747858	0.6605469	0.6540778	0.5369078	3.2158277
		HORVU.MOREX.r3.7HG0683600	-0.737784	-0.076769	0.9518875	3.8406589	0.4515704	1.6510555	3.2807667	4.2069782
		HORVU.MOREX.r3.7HG0684180	-0.279358	-0.935076	-0.732582	-2.097475	-0.128102	-0.355632	-1.69092	-2.680091
	MQTL7.3	HORVU.MOREX.r3.7HG0686880	0.6647448	0	1.3201815	2.1205031	1.2862849	0.9592199	1.073598	0.6015589
		HORVU.MOREX.r3.7HG0686900	-0.369452	-1.54166	-0.112699	-2.151211	-0.119639	-0.221625	-0.819283	-3.172782
		HORVU.MOREX.r3.7HG0686980	1.1273493	4.2458855	1.0590442	2.8140662	0.9602623	1.7526858	2.2859437	3.0449059
		HORVU.MOREX.r3.7HG0687050	-0.049035	-0.458484	-0.353719	-1.306452	-0.219185	-0.274866	-1.7104	-2.19428

Continued Table S5.

Meta-QTL	Gene	Shoot (35.5)	Root (35.5)	PEG (6h)	PEG (24)	NaCl (6H)	NaCl (24h)	PEG+NaCl (6h)	PEG+NaCl (24)
MQTL7.4	HORVU.MOREX.r3.7HG0687790	0.2325146	0.3130518	0.0645662	2.1050516	0.2982355	0.4960735	1.1475583	2.4125479
	HORVU.MOREX.r3.7HG0688580	-0.244882	-1.302885	-0.750087	-1.942548	-0.64684	-0.208699	-1.208138	-2.174803
	HORVU.MOREX.r3.7HG0689170	-1.402201	-2.199944	-1.030613	-2.201355	-0.966047	-0.637551	-1.60983	-2.382935
MQTL7.5	HORVU.MOREX.r3.7HG0691850	2.3173438	2.8639228	-0.060493	-0.150619	0.051728	-0.100993	-0.06894	-0.109642
MQTL7.9	HORVU.MOREX.r3.7HG0699630	0	-0.17702	0.3626772	2.5277469	0.2622025	0.6711879	2.0868145	3.0306822
	HORVU.MOREX.r3.7HG0700030	-1.15837	-2.129409	-0.154158	-1.136577	-0.357778	-0.355526	0.2999442	-1.178255
MQTL7.10	HORVU.MOREX.r3.7HG0703260	0.0543568	-0.336241	1.0710302	2.414652	0.9361539	1.0557386	2.4536504	2.3600525
