# Supplementary material for: Mega Meta-QTLs: A Strategy for the Production of Golden Barley (Hordeum vulgare L.) Tolerant to Abiotic Stresses
Source: Genes (Basel). 2022 Nov 10;13(11):2087. doi: 10.3390/genes13112087 (PMC9690463; doi:10.3390/genes13112087)
Supplement: Supplementary file 1 [file genes-13-02087-s001.zip › Table S6.pdf]

Table S6. Biological Process, Molecular Function, Cellular Component and their number of genes

Biological Process	Number of Gene	Molecular Function	Number of Gene	Cellular Component	Number of Gene
cellular process	71	binding	68	cellular anatomical entity	73
metabolic process	59	catalytic activity	63	membrane	38
response to stimulus	28	transferase activity	22	intracellular anatomical structure	25
primary metabolic process	28	protein binding	21	organelle	19
organic substance metabolic process	28	organic cyclic compound binding	21	cytoplasm	17
response to stress	23	heterocyclic compound binding	21	extracellular region	16
catabolic process	21	transporter activity	12	cell periphery	10
nitrogen compound metabolic process	21	hydrolase activity	11	endomembrane system	5
localization	18	small molecule binding	10	external encapsulating structure	3
establishment of localization	18	molecular function regulator	4	cytosol	2
response to chemical	16	transcription regulator activity	4		
cellular metabolic process	16	enzyme regulator activity	4		
biosynthetic process	13	DNA-binding transcription factor activity	4		
biological regulation	8	carbohydrate binding	1		
regulation of molecular function	6				
cellular component organization or biogenesis	6				
response to abiotic stimulus	4				
multicellular organismal process	3				
developmental process	3				
response to endogenous stimulus	3				
anatomical structure development	3				
response to biotic stimulus	2				
response to external stimulus	2				
post-embryonic development	2				
reproduction	1				
signaling	1				
regulation of biological quality	1				
cellular response to stimulus	1				
regulation of biological process	1				
cell communication	1				
